# Comparative genomics and evolution of transcriptional regulons in *Proteobacteria*

**DOI:** 10.1099/mgen.0.000061

**Published:** 2016-07-11

**Authors:** Semen A. Leyn, Inna A. Suvorova, Alexey E. Kazakov, Dmitry A. Ravcheev, Vita V. Stepanova, Pavel S. Novichkov, Dmitry A. Rodionov

**Affiliations:** ^1^​ A. A. Kharkevich Institute for Information Transmission Problems, Russian Academy of Sciences, Moscow, Russia; ^2^​ Lawrence Berkeley National Laboratory, Berkeley, CA 94720, USA; ^3^​ Luxembourg Centre for System Biomedicine, Esch-Belval, Luxembourg; ^4^​ Sanford-Burnham-Prebys Medical Discovery Institute, La Jolla, CA 92037, USA

**Keywords:** comparative genomics, transcription factor, amino acid metabolism, *Proteobacteria*

## Abstract

Comparative genomics approaches are broadly used for analysis of transcriptional regulation in bacterial genomes. In this work, we identified binding sites and reconstructed regulons for 33 orthologous groups of transcription factors (TFs) in 196 reference genomes from 21 taxonomic groups of *Proteobacteria*. Overall, we predict over 10 600 TF binding sites and identified more than 15 600 target genes for 1896 TFs constituting the studied orthologous groups of regulators. These include a set of orthologues for 21 metabolism-associated TFs from *Escherichia coli* and/or *Shewanella* that are conserved in five or more taxonomic groups and several additional TFs that represent non-orthologous substitutions of the metabolic regulators in some lineages of *Proteobacteria*. By comparing gene contents of the reconstructed regulons, we identified the core, taxonomy-specific and genome-specific TF regulon members and classified them by their metabolic functions. Detailed analysis of ArgR, TyrR, TrpR, HutC, HypR and other amino-acid-specific regulons demonstrated remarkable differences in regulatory strategies used by various lineages of *Proteobacteria*. The obtained genomic collection of *in silico* reconstructed TF regulons contains a large number of new regulatory interactions that await future experimental validation. The collection provides a framework for future evolutionary studies of transcriptional regulatory networks in *Bacteria*. It can be also used for functional annotation of putative metabolic transporters and enzymes that are abundant in the reconstructed regulons.

## Data Summary

1. Inferred transcription factor binding sites and reconstructed regulons have been deposited in the RegPrecise database (URL – http://regprecise.lbl.gov/RegPrecise/project_proteobacteria.jsp).

## Impact Statement

Bacteria in most ecological niches are constantly exposed to variations in many factors, including nutrient availability. Changes in gene expression using transcription factors (TFs) allow bacteria to adapt to these variations. Knowledge of transcriptional regulatory networks is essential for understanding cellular processes. Comparative genomics is the analysis and comparison of genomes from different species. Thousands of sequenced bacterial genomes provide an opportunity to reconstruct transcriptional regulatory networks using comparative genomics. Despite the importance of transcriptional regulation of the central metabolism for systems-level metabolic modelling of *Bacteria*, our understanding of the respective transcription factor regulons is limited for the majority of sequenced bacteria. In this study, we have applied the comparative genomics approach to describe regulatory networks of genes involved in central metabolism in four major classes of *Proteobacteria*. The reconstructed regulatory networks involve 33 groups of orthologous TFs with different DNA recognition motifs. Large-scale phylogenomic analysis of the reconstructed TF regulons reveals and classifies various evolutionary processes that shape regulatory networks in *Bacteria*. The reconstructed regulon contents suggest numerous novel functional associations between both known and uncharacterized genes encoding enzymes and transporters, thus providing testable hypotheses for future experimental studies. This study demonstrates the power of comparative genomics for the reconstruction of TF regulons in bacteria.

## Introduction

Regulation of gene expression is an important mechanism for fast adaptation of prokaryotic metabolism to changing environmental conditions. Transcription factors (TFs) repress or activate gene transcription via specific binding to TF binding sites (TFBSs) in regulatory gene regions. The binding ability of many bacterial TFs depends on the presence or absence of an effector, such as intracellular metabolites, inorganic chemicals or physical stimuli (Browning & Busby, 2004). A set of genes directly controlled by a single TF is called a regulon. Global TF regulons in bacteria contain large sets of genes (operons) that share similar TFBSs in their promoter regions, while local TFs control one or several operons that are often co-localized with a TF gene (Rodionov, 2007).

Experimental studies have built a foundation for understanding the mechanisms behind transcription regulation (Minchin & Busby, 2009). However, even with high-throughput technologies as ChIP-Seq or RNA-Seq, these approaches still consume a lot of time and resources and therefore are restricted to a few model organisms (Grainger *et al.*, 2009). At this point, comparative genomics studies of a growing number of sequenced bacterial genomes provide a successful approach to extend our knowledge of known TF regulons to a wide range of bacterial lineages, as well as to perform *ab initio* prediction of novel TF regulons (Rodionov, 2007). Comparative genomics-based regulon reconstruction combines identification of conserved *cis*-acting TFBSs, and their genomic and metabolic context analysis in a set of closely related genomes. Finally, it results in determination of a regulog, i.e. a set of genes/operons co-regulated by orthologous TFs in closely related organisms. Implementation of this approach in the RegPredict web tool (Novichkov *et al.*, 2013) produced numerous computational reconstructions of TF regulogs across a wide range of bacterial taxa (Ravcheev *et al.*, 2011, 2013; Rodionov *et al.*, 2011, 2013; Leyn *et al.*, 2013). The substantial amount of data on regulon reconstructions captured in the RegPrecise database (Novichkov *et al.*, 2013) provides the basis for description of most common types of events associated with the evolution of TF regulons in bacteria, such as duplications and losses of TFs and their TFBSs that result in expansions, shrinkages, mergers and split-ups of regulons (Gelfand, 2006; Rodionov *et al.*, 2006; Ravcheev *et al.*, 2014). New non-orthologous TFs could be introduced to control equivalent pathways or, vice versa, orthologous TFs could control distinct pathways in related taxonomic groups of bacteria (Yang *et al.*, 2006; Rodionov *et al.*, 2008; Kazakov *et al.*, 2009; Leyn *et al.*, 2014).

We recently conducted a comprehensive comparative genomics analysis of regulatory systems for methionine metabolism in nearly 200 representative genomes from 22 taxonomic groups from the phylum *Proteobacteria* (Leyn *et al.*, 2014). In he *Gammaproteobacteria*, two TFs, MetJ and MetR, are implicated in the control of methionine metabolism, whereas this function is taken by other TFs (SahR and SamR) or RNA regulatory systems (e.g. SAH and SAM riboswitches) in other lineages of *Proteobacteria*. The core of MetJ regulons includes a large number of genes that are highly conserved in most lineages of gammaproteobacteria. In contrast, the core of MetR regulons includes only two genes, *metE* and *metR*, whereas regulatory interactions between MetR and other target genes are mostly lineage-specific. Regulatory system replacement and lineage-specific regulon expansions in *Proteobacteria* were also observed in the comparative genomics analyses of TF regulons involved in fatty acid degradation (FadR, PsrA, FadP), branched-chain amino acid utilization (LiuR, LiuQ), *N*-acetylglucosamine utilization (NagC, NagR, NagQ) (Yang *et al.*, 2006; Kazakov *et al.*, 2009), biotin biosynthesis (BirA, BioR) (Rodionov & Gelfand, 2006) and central carbohydrate metabolism (HexR) (Leyn *et al.*, 2011).

Here, we extended these observations toward large-scale regulon reconstructions for 21 known TFs that have orthologues in a wide phylogenetic range of *Proteobacteria*. By comparing the metabolic context of the reconstructed TF regulons, we identified the core, taxonomy-specific and genome-specific members of regulons, and proposed evolutionary scenarios for the regulation of several pathways involved in the metabolism of amino and fatty acids, nucleotides and co-factors in *Proteobacteria*. Additionally, we predicted novel regulators of aromatic amino acid metabolism replacing the TyrR/PhrR and HmgR regulons in *Alteromonadales* and *Pseudomonadales* (named HmgS and HmgQ), and a novel regulator of NAD metabolism in betaproteobacteria and alphaproteobacteria, named NadQ. The obtained regulatory reconstructions for both known and new TF regulons across 196 reference genomes of *Proteobacteria* will be useful for development of theoretical models for the evolution of microbial regulatory networks.

## Methods

For regulon reconstruction, we selected 196 reference genomes of alpha-, beta-, gamma and deltaproteobacteria and subdivided them into 21 sets of evolutionarily related genomes (Table S1, available in the online Supplementary Material). Closely related strains and species were excluded from the analysis because they skew the TFBS training set and thus decrease the sensitivity of the TFBS recognition rule. Genomes and the phylogenetic species tree were downloaded from MicrobesOnline database (Dehal *et al.*, 2010). Each taxonomic group includes 4–16 genomes of bacteria. Orthologues of TFs in the selected genomes were identified as bidirectional best hits using protein blast searches (Altschul *et al.*, 1997) and were additionally confirmed via phylogenetic trees using precomputed protein trees in MicrobesOnline. The genomes of epsilonproteobacteria were not analysed because of the absence of orthologues for target TFs. Zetaproteobacteria, which is represented by a single genome in the MicrobesOnline database, was not suitable for the comparative genomic analysis.

Genes in the reconstructed regulons were considered orthologues if they were classified as specific tree-based orthologues in MicrobesOnline. Conservancy of the genomic context through related genomes was considered as an additional support for gene orthology. Comparative analysis of conserved gene neighborhoods was conducted in MicrobesOnline. Biological functions of genes were predicted by blast searches against the SwissProt/Uniprot database (UniProt C, 2014) , by domain architecture analysis in the Pfam database (Finn *et al.*, 2014) and by using gene function assignments in the PubSEED database (Overbeek *et al.*, 2005). Known metabolic pathways were taken from KEGG (Kanehisa & Goto, 2000) and EcoCyc (Karp *et al.*, 2014). Sequence logos for TF binding sites were drawn using the WebLogo package (Crooks *et al.*, 2004).

For regulon reconstruction we used an established comparative genomics approach implemented in the RegPredict interactive tool (Novichkov *et al.*, 2013). This approach is based on construction of positional weight matrices (PWMs) for TFBS motifs, and further genomic searches for additional regulon members on the basis of predicted TFBSs in upstream gene regions (Rodionov, 2007). The bioinformatics workflow used for regulon reconstruction is described in Fig. S1. Two main workflows were applied for regulon reconstructions: (i) propagation and expansion of known TF regulons that were previously experimentally studied in model organisms (Table S2) and/or computationally reconstructed in *Shewanella* species (Rodionov *et al.*, 2011); and (ii) *ab initio* prediction of novel TF regulons for sets of potential target genes involved in the same metabolic pathway. To find conserved TFBS motifs for the known TFs in each taxonomic group where their orthologues are present, we used initial training sets of genes that are orthologous to previously established regulon members in model species, and then updated each set by potential regulon members confirmed by the comparative genomics checks. For novel TF regulons, the original training sets included genes from the respective metabolic pathways and/or conservative chromosomal gene neighbourhoods around analysed TFs.

A simple iterative procedure implemented in the Discover Profile tool in RegPredict was used for identification of conserved palindromic DNA motifs and construction of PWMs. For most of the analysed TFs, their DNA motifs have palindromic structure and length between 15 and 25 nt, whereas the TFBS motifs of NagQ and BirA represent tandem and inverted repeats, respectively. The obtained PMWs (both known and *ab initio* predicted) were further used for identification of additional candidate sites in upstream gene regions as previously described (Leyn *et al.*, 2014; Ravcheev *et al.*, 2014). Each predicted regulatory interaction was analysed for conservation within the analysed groups of genomes using the Clusters of co-Regulated Orthologous operoNs (CRONs) approach in RegPredict. Further analysis of functional and genomic context and curation of each CRON resulted in the final TF regulon model. All reconstructed TF regulons including TFBS motifs and sets of TF-regulated genes/operons with their functional annotations are accessible in the latest release of the RegPrecise database (Novichkov *et al.*, 2013) (Data Citation 1). Each TF regulon in RegPrecise belong to two types of regulon collections classified by either taxonomy of studied bacteria, or by the name of TFs.

## Results and Discussion

### Statistics of reconstructed regulons and regulogs

A set of 196 representative genomes of gamma-, beta-, alpha- and deltaproteobacteria selected from the MicrobesOnline database was classified into 21 taxonomic groups by analysing the phylogenetic species tree (Table S1). For the analysis of evolution of transcriptional regulation, we selected a set of 21 transcriptional regulators of central metabolism that are present either in *E. coli* and/or *Shewanella* species and that are conserved in five or more taxonomic groups of *Proteobacteria* (Table 1; Fig. S1). The selected TFs include the previously known regulators that control biosynthesis/utilization of amino acids (ArgR, HutC, HypR, LiuR, MetJ, MetR, TrpR, TyrR), fatty acids (FabR, FadR, PsrA), nucleotides (NrdR, RutR) and vitamins (BirA, NrtR), as well as nitrogen and carbon metabolism (HexR, GlcC, LldR, NagC, NtrC, PdhR). Sixteen of these TFs are present in *E. coli*, of which 12 regulators are also shared by *Shewanella* species, whereas the remaining five TFs (LiuR, HutC, HypR, NrtR, PsrA) are unique for *Shewanella* species. We also studied 12 additional TFs that appear to substitute for some of the above TFs in the control of specific metabolic pathways, and thus can be assumed to benon-orthologous TF replacements. These include known and predicted regulators that control metabolism of amino acids (HmgQ, HmgR, HmgS, LiuQ, SahR, SamR) and fatty acids (FadP), vitamin biosynthesis (BioR, NadR, NadQ) and *N*-acetyl-glucosamine metabolism (NagQ, NagR) (marked with an asterisk in Table 1).

**Table 1.** Statistics for the studied TF regulons in *Proteobacteria* Initially, we studied 21 TFs that are present in *E. coli* and/or *Shewanella* species and that are conserved in five or more taxonomic groups of *Proteobacteria*. Additionally, we studied TFs that represent non-orthologous replacements of the initial set of TFs in some taxonomic groups (marked by an asterisk).

TF†	Protein family	Metabolic pathways controlled by a TF	Genomes (taxa)‡	TFBSs, total§	Genes, total§	Genes, average||
ArgR	ArgR	Arginine metabolism	62 (6)	1079	1223	19.7
BioR*	GntR	Biotin biosynthesis	13 (2)	34	59	4.5
BirA	BirA	Biotin biosynthesis	94 (11)	185	495	5.3
FabR	TetR	Fatty acid biosynthesis	74 (10)	361	392	5.3
FadP*	TetR	Fatty acid degradation	25 (3)	194	448	17.9
FadR	GntR	Fatty acid degradation	61 (6)	374	423	6.9
GlcC	GntR	Glycolate utilization	23 (7)	83	133	5.8
HexR	RpiR	Central carbohydrate metabolism	87 (11)	897	1178	13.5
HmgQ*	LysR	Tyrosine degradation	17 (2)	35	50	2.9
HmgR*	IclR	Tyrosine degradation	5 (1)	12	24	4.8
HmgS*	MarR	Tyrosine degradation	3 (1)	6	9	3.0
HutC	GntR	Histidine utilization	113 (18)	386	857	7.6
HypR	GntR	Proline/4-hydrohyproline utilization	44 (12)	190	265	6.0
LiuQ*	TetR	Branched-chain amino acid utilization	14 (3)	54	73	5.2
LiuR	MerR	Branched-chain amino acid utilization	103 (15)	671	1411	13.7
LldR	GntR	Lactate utilization	55 (13)	146	241	4.4
MetJ	MetJ	Methionine metabolism	62 (6)	1026	857	13.8
MetR	LysR	Methionine metabolism	117 (14)	570	480	4.1
NadR*	NadR	NAD biosynthesis	11 (1)	27	35	3.2
NadQ*	NadQ	NAD biosynthesis	30 (7)	67	109	3.6
NagC	ROK	*N*-cetylglucosamine utilization	31 (5)	304	431	13.9
NagQ*	GntR	*N*-cetylglucosamine utilization	31 (10)	81	225	7.3
NagR*	LacI	*N*-cetylglucosamine utilization	25 (4)	168	288	11.5
NrdR	NrdR	Deoxyribonucleotide biosynthesis	186 (20)	638	591	3.2
NrtR	NrtR	NAD biosynthesis	28 (11)	75	96	3.4
NtrC	Fis	Nitrogen assimilation	169 (19)	921	1804	10.7
PdhR	GntR	Pyruvate metabolism	55 (6)	200	399	7.3
PsrA	TetR	Fatty acid degradation	76 (12)	673	845	11.1
RutR	TetR	Pyrimidine catabolism	68 (13)	273	743	10.9
SahR*	ArsR	Methionine metabolism	62 (9)	156	266	4.3
SamR*	ArsR	Methionine metabolism	4 (1)	17	30	7.5
TrpR	TrpR	Aromatic amino acid metabolism	53 (8)	142	314	5.9
TyrR	TyrR	Aromatic amino acid metabolism	67 (7)	618	896	13.4

‡Number of genomes and taxonomic groups (in parentheses) of *Proteobacteria* that contain the studied TF regulon. The detailed distribution of regulons and regulogs is provided in Fig. S2.

§Total number of candidate TFBSs and TF-regulated genes (target genes) in all studied genomes.

||Average number of candidate TFBSs per genome.

Application of the comparative genomics procedure to 33 analysed groups of orthologous TFs resulted in reconstruction of 283 regulogs containing 1896 regulons that are unevenly distributed across 21 taxonomic groups of *Proteobacteria* (Fig. S2). Each regulon includes a set of target genes/operons that are co-regulated by the same TF in a particular genome. A regulog represents a set of regulons under control of orthologous TFs in a specific taxonomic group of *Proteobacteria*. The most widespread orthologous groups of analysed TFs are NrdR (186 regulons, 20 regulogs), NtrC (169 regulons, 19 regulogs), MetR (117 regulons, 14 regulogs), HutC (113 regulons, 18 regulogs), LiuR (104 regulons, 16 regulogs), HexR (95 regulons, 13 regulogs) and BirA (94 regulons, 11 regulogs).

**Fig. 1.**
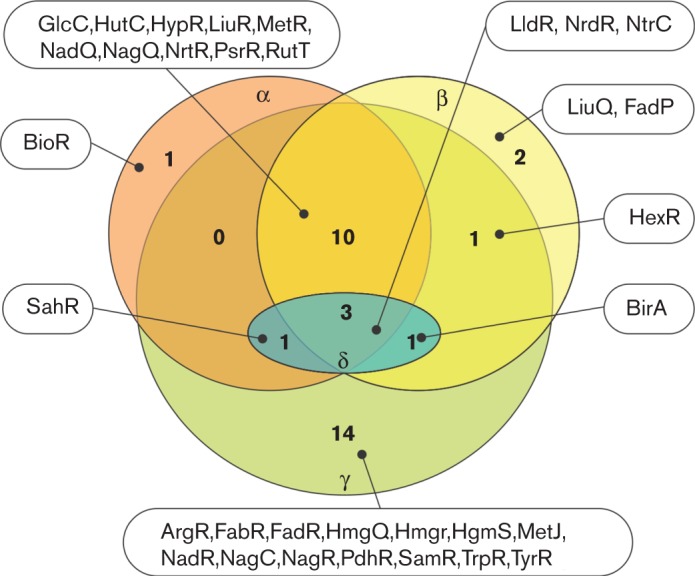
Taxonomic distribution of 33 studied TF regulons in four major classes of *Proteobacteria*. Circles include the number of TFs that are either taxonomic class-specific or shared between several classes.

The taxonomic distribution of analysed TF regulogs across four subdivisions from the phylum *Proteobacteria* is summarized in Fig. 1. Overall, 30 out of 33 analysed TFs are present in gammaproteobacteria, and 14 of these regulators do not have orthologues in other classes of *Proteobacteria*. Alphaproteobacteria possess, in total, 15 studied TFs, including one regulator (BioR), which is unique for this class. Among 17 studied TFs in betaproteobacteria, two regulators (LiuQ, FadP) are unique for this class. Deltaproteobacteria, which represent the most taxonomically diverse subdivision of *Proteobacteria,* have orthologues for only five studied TFs. Several TFs (such as LldR, GlcC, RutR) that are present in several classes of *Proteobacteria* show a mosaic distribution across the analysed genomes and taxa, while other TFs (such as ArgR, FabR, FadR, MetJ, TrpR and TyrR) are highly conserved in many taxonomic groups of gammaproteobacteria but are absent in other classes. The diverse distribution of TFs suggests different evolutionary pathways for the studied metabolic regulons.

The detailed descriptions of reconstructed regulons and regulogs are captured in the RegPrecise database (Data Citation 1), whereas the complete list of regulatory interactions between the studied TFs and their target genes is provided in Table S3. Overall, the obtained regulons included 10 663 candidate TFBSs and 15 690 target genes (Table 1). The largest average number of target genes per genome (more than 10 genes per genome) was observed for regulators of amino acid metabolism (ArgR, LiuR, MetJ, TyrR), the fatty acid degradation regulators FadP and PsrA, the carbohydrate metabolism regulators HexR, NagC and NagR, and the pyrimidine utilization regulator RutR.

**Fig. 2.**
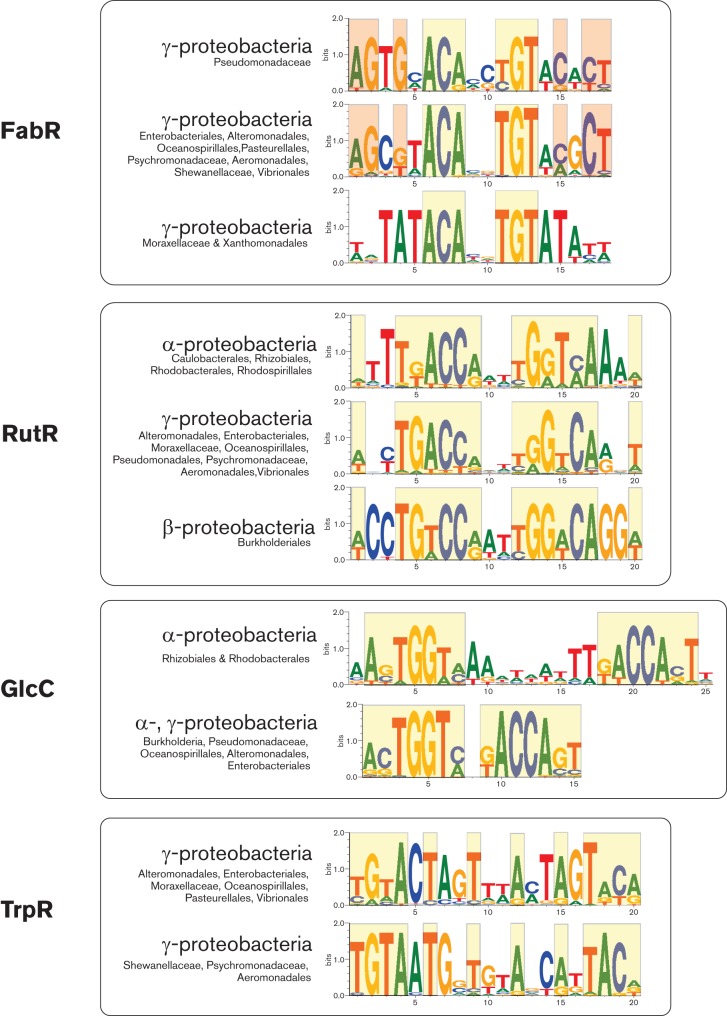
Examples of motif changes in four orthologous groups of studied TFs. Conservative positions between motifs within groups are boxed with the same color.

For most of the studied TFs, their cognate DNA binding motifs are generally conserved across the analysed taxonomic groups (see the RegPrecise database for detailed lists of taxonomy-specific TFBS motifs, Data Citation 1). However, for several TFs including FabR, HypR, NrtR, RutR, SahR and TrpR, we observed taxon-specific substitutions in their cognate DNA motifs, whereas the GlcC-binding DNA motifs in the alpha- and beta-/gamma-subdivisions of *Proteobacteria* are characterized by different length of the spacer between the conserved palindromic half-sites (Fig. 2). Finally, the HexR and NagQ motifs in several taxonomic groups of gammaproteobacteria have different consensus sequences and structures (Yang *et al.*, 2006; Leyn *et al.*, 2011).

### Conservation of reconstructed regulons

To analyse conservation of regulatory interactions in the reconstructed regulogs, we calculated the conservation score as the number of gene occurrences in a regulog divided by the number of regulons in a regulog. The mean of these taxonomy-specific conservation scores was calculated for all orthologous groups of target genes across analysed lineages of *Proteobacteria*. For each group of orthologous TFs, we plotted the average conservation score of a target gene against the number of taxonomic groups, in which this gene is regulated. The obtained plots visualize average conservation of regulatory interactions and thus help to determine the core, taxonomy-specific and genome-specific target genes within the reconstructed TF regulons (Fig. S3).

**Fig. 3.**
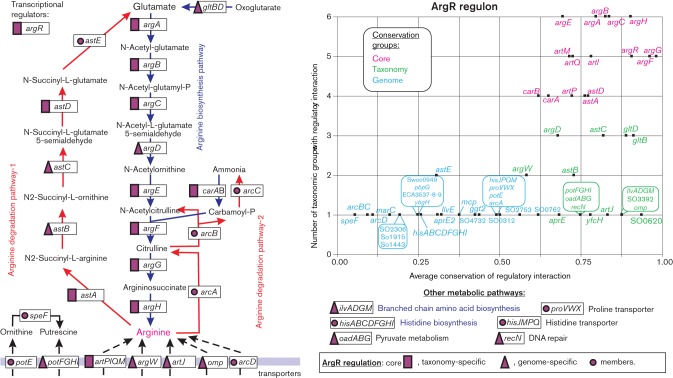
Arginine metabolism and its regulation by ArgR in *Proteobacteria*.

The core regulon members determined by this approach represent regulatory interactions with high average conservation scores that are conserved in more than half of reconstructed TF regulogs. The core members of most of the analysed regulons are consistent with major biological functions and molecular effectors of their cognate TFs (Table S4). For instance, the arginine repressor regulon ArgR in gammaproteobacteria has a conserved core that includes genes involved in arginine biosynthesis (*argABCEFGH*, *carAB*), transport (*artPIQM*) and degradation (*astAD*), as well as the *argR* gene itself (Fig. 3). The cores of most other reconstructed TF regulons include their cognate TF genes. Exceptions to this observation include the FabR, FadR and NadQ regulons that include their cognate TF genes only in some taxonomic groups of *Proteobacteria*; the biotin repressor BirA, which is autoregulated only in *Desulfovibrionales*; and the deoxyribonucleotide reductase regulator NrdR, which was never found under autoregulation. Negative autoregulation of a TF gene is a common feature of bacterial regulatory networks. Here, we demonstrate that this type of regulatory interaction is highly conserved in the evolution of regulatory networks of *Proteobacteria*.

The remaining members of reconstructed regulons were classified into the taxon- and genome-specific groups depending on their average conservation scores. The taxonomy-specific regulon members are characterized by strong conservation of regulatory interactions restricted to 50 % or less of taxonomic groups containing an orthologous TF. Thus, taxon-specific regulon members were defined as genes that are regulated in more than 65 % of genomes in at least one taxonomic group. In contrast, the genome-specific regulon members are characterized by low conservation of regulatory interactions, when in each taxonomic group conservation of a regulatory interaction is less than 65 %. The groups of taxonomy-and genome-specific TF regulon members with assigned metabolic pathways are often involved in the same major biological process as the core regulon members (Table S4). However, in some TF regulons these categories also involve genes that participate in other metabolic pathways or biological processes. For example, the taxon-specific members of ArgR regulons include genes involved in arginine biosynthesis (*argD*) and transport (*artJ, argW, omp*), arginine degradation (*astBC*), as well as genes from glutamate (*gltBD*) and branched-chain amino acid (*ilvMGDA*) biosynthesis, putrescine transport (*potFGHI*) and pyruvate metabolism (*oadABG*) (Fig. 3). The genome-specific ArgR regulon members include arginine degradation genes (*arcABCD, astE*), proline (*proVWX*) and histidine (*hisJMPQ*) transporters, and putrescine metabolism genes (*speF, potE*). Likewise, in our previous analysis of the methionine-specific regulons MetJ, MetR and SahR in *Proteobacteria*, we identified the core-, taxonomy- and genome-specific members of regulons and demonstrated their involvement in different aspects of the methionine metabolism. Other amino-acid-specific TF regulons analysed in the current work are described in more detail in the following sections.

### TrpR, TyrR and other TF regulons for aromatic amino acid metabolism

**Fig. 4.**
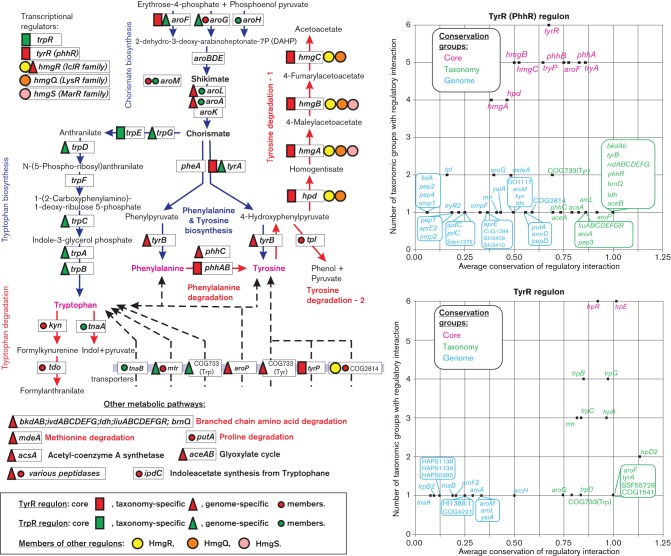
Aromatic amino acid metabolism and its regulation by TyrR, TrpR and other transcription factors in *Proteobacteria*.

The aromatic amino acids tryptophan, tyrosine and phenylalanine are synthesized in *Proteobacteria* by the common pathway leading from erythrose 4-phosphate to 2-dehydro-3-deoxy-d-arabinoheptonate-7-phosphate (DAHP), and shikimate to chorismate (Fig. 4). After chorismate, the pathway divides into the three terminal biosynthetic pathways that are specific for each aromatic amino acid. *E. coli* has three DAHP synthase isoenzymes, AroF, AroG and AroH, which are feedback inhibited by tyrosine, phenylalanine and tryptophan, respectively. The biosynthesis of aromatic amino acids is regulated at both the DNA and the RNA level. The DNA-binding transcription factors TyrR and TrpR jointly control the expression of genes involved in aromatic amino acid metabolism in *E. coli* (Pittard & Yang, 2008). At the RNA level, the *trpEDCBA* operon encoding the tryptophan biosynthesis enzymes and the phenylalanine biosynthesis gene *pheA* are regulated by translational attenuation in *E. coli* and other gammaproteobacteria (Panina *et al.*, 2001). The tryptophan-responsive regulator TrpR in *E. coli* acts as a repressor of the *trpEDCBA* operon, the tryptophan transporter gene *mtr* and the regulatory gene *trpR* (Czernik *et al.*, 1994; Jeeves *et al.*, 1999). In addition, TrpR negatively regulates the expression of the shikimate kinase *aroL* and the DAHP synthase *aroH* that are involved in chorismate biosynthesis. The tyrosine-responsive regulator TyrR in *E. coli* negatively controls the tyrosine biosynthesis genes *tyrB*, *aroF-tyrA* and *aroLM*,the aromatic amino acid transporter *aroP* and the *tyrR* gene itself. In addition, TyrR activates the tyrosine- and tryptophan-specific transporters *tyrP* and *mtr* and the folate biosynthesis gene *folA* in the presence of tyrosine or phenylalanine (Yang *et al.*, 2004; Pittard *et al.*, 2005). The TyrR regulon was also partially studied in two other *Enterobacteria*. In *Citrobacter freundii*, it activates the tyrosine degradation gene *tpl* (Smith & Somerville, 1997). In *Enterobacter cloacae*, TyrR activates the *ipdC* gene involved in the synthesis of indole acetate from tryptophan and represses a divergently transcribed gene, *akr*, encoding a putative aldo-keto reductase (Coulson & Patten, 2015). An orthologue of TyrR in *Pseudomonas putida*, known as PhhR, is responsible for the activation of genes essential for phenylalanine degradation and phenylalanine homeostasis (Herrera *et al.*, 2010). In *Pseudomonas aeruginosa*, PhhR directly controls the *phhABC*, *hpd* and *dhcA* transcriptional units involved in the catabolism of phenylalanine and tyrosine (Palmer *et al.*, 2010).

The comparative genomics approach was applied to analyse regulons controlled by transcription factors homologous to TrpR and TyrR/PhhR and to predict novel regulons for aromatic amino acid metabolism (Table S5). The TrpR-family regulons reconstructed in eight lineages of gammaproteobacteria control genes for tryptophan biosynthesis, uptake and catabolism (Fig. 4). In most of the analysed taxonomic groups, TrpR regulates the *trpR* and *trpE* genes that form the conserved regulon core, whereas other genes from the tryptophan biosynthesis pathway and the *mtr* transporter were classified as taxon-specific members of the regulon. Two other known targets of TrpR in *E. coli*, the chorismate biosynthesis genes *aroLM* and *aroH*, represent regulatory interactions that are conserved in a small number of closely related *Enterobacteriales* genomes but not in other lineages. New predicted members of the TrpR regulons include various aromatic amino acid biosynthesis genes such as *aroG* in *Pasteurellales*, *aroFtyrA* in *Shewanellaceae*, *aroF2* and *aroA* in *Vibrionales*, as well as the tryptophan degradation genes *tnaAB* and a predicted tryptophan transporter from the COG0733 family in *Vibrionales*. Interestingly, the TrpR regulon in *Shewanella* species does not include the tryptophan biosynthesis operon, which is regulated by a translational attenuator at the RNA level (Panina *et al.*, 2001).

The content of reconstructed TyrR (PhhR) regulons is highly variable across the analysed six lineages of gammaproteobacteria (Table S5). The most conserved members of these regulons are the *tyrR* gene itself, the tyrosine biosynthesis and transport genes *aroFtyrA* and *tyrP*, as well as the phenylalanine and tyrosine degradation genes *phhAB, hmgABC* and *hpd* (Fig. 4). The aromatic amino acid transporters *mtr* and *aroP*, as well as the chorismate biosynthesis genes *aroLM*, which were previously known as TyrR-regulated genes in *E. coli*,belong to the TyrR regulons only in *Enterobacteriales*. The *folA* gene represents another previously known member of the TyrR regulon in *E. coli*, although we were unable to find conserved TyrR-binding sites upstream of *folA* orthologues in other *Enterobacteriales*.

A novel predicted tyrosine transporter from the COG2814 family was found under TyrR regulation in seven genomes of *Enterobacteriales*. The reconstructed TyrR regulons in *Vibrionales* are extended to include a predicted novel tyrosine transporter from the COG0733 family and the *aroG* gene. The most significant shifts in the regulon content were identified in *Shewanellaceae*, where TyrR controls the degradation pathways for various amino acids including phenylalanine (*phhAB*), tyrosine (*hmgCB*), tryptophan (*tdo-kyn*), branched chain amino acids (*ldh*, *brnQ*, *liu*, *ivd* and *bkd* operons), proline (*putA*), methionine (*mdeA*) and oligopeptides (various peptidase genes). In addition, the conserved part of the TyrR regulons in *Shewanellaceae* includes the tyrosine/phenylalanine biosynthesis genes *aroA* and *tyrB*, as well as the *aceBA* and *acsA* genes from central carbon metabolism. Finally, the *tpl* gene encoding an alternative pathway of tyrosine degradation belongs to the TyrR regulons in *Citrobacter koseri* and two *Pasteurellales*, whereas the indole acetate synthesis gene *ipdC* is regulated by TyrR in some *Enterobacteriales* and *Shewanellaceae* genomes.

The homogentisate pathway of tyrosine degradation encoded by the *hmgABC* operon in *P. putida* is regulated by the IclR-family repressor HmgR and homogentisate as anti-repressor (Arias-Barrau *et al.*, 2004). Similar HmgR regulons were reconstructed in four other *Pseudomonas* species, whereas the *hmgABC* genes in two other *Pseudomonas* species belong to the TyrR-family PhrR regulons (Table S5). In a closely related bacterium from the family *Pseudomonadaceae*, *Azotobacter vinelandii*, which lacks both PhrR and HmgR regulons, we identified a novel LysR-family regulon for the homogentisate pathway genes, which we termed HmgQ. Orthologous HmgQ regulators in the *Shewanellaceae* family are predicted to control the *hmgA*–*hpd* genes, whereas the *hmgCB* genes of the homogentisate pathway belong to the extended TyrR regulon in *Shewanella* species. Another novel regulator from the MarR family (termed HmgS) was identified in several *Alteromonadales* and *Pseudoalteromonadales* species, where it is predicted to control the *hmgAB* genes.

In conclusion, the transcriptional regulation of aromatic amino acid metabolism is highly variable among major lineages of gammaproteobacteria (Table S5). The TyrR-family regulators control the aromatic amino acid biosynthesis, uptake and/or catabolic pathways in most of the analysed taxa. In contrast, the reconstructed regulons in the family *Shewanellaceae* predict a global regulatory role of TyrR for genes that are involved in catabolism of various amino acids and in central carbon metabolism. The mode of TyrR action on its predicted novel targets in *Shewanella* is to be determined experimentally. Preliminary comparative analysis of positions of the TyrR-binding sites in the promoter gene regions suggest that TyrR probably acts as an activator for most of the amino acid degradation operons in *Shewanella* species (data not shown). The homogentisate pathway in gammaproteobacteria is controlled either by TyrR/PhhR or by non-orthologous local regulators from the IclR, LysR, and MarR protein families. We also observed interchangeability between the TyrR and TrpR regulons: the *aroF*–*tyrA* genes are controlled by TyrR in most of the analysed lineages, whereas in the family *Shewanellaceae* this operon is predicted to be controlled by TrpR. Overall, the major biological role of TyrR in *Enterobacteriales* and *Pasteurellales* is the regulation of aromatic amino acid biosynthesis and transport genes, although in *Pseudomonas* and *Shewanella* it mostly controls the amino acid degradation pathways, whereas in other *Alteromonadales* species, as well as in *Vibrionales* and *Aeromonadales*, it is implicated in the control of both biosynthetic and catabolic pathways.

### HypR regulon for proline and 4-hydrohyproline utilization

**Fig. 5.**
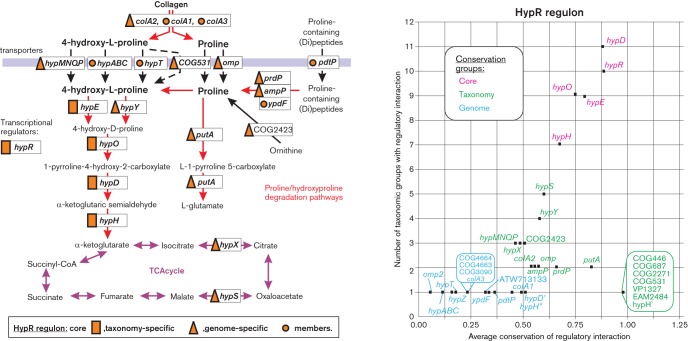
Hydroxyproline and proline utilization pathway and its regulation by HypR in *Proteobacteria*.

l-Proline, the only proteinogenic imino acid, is used by many bacteria as a source of energy and a precursor for the synthesis of other amino acids. 4-Hydroxy-l-proline, which can be synthesized post-translationally from l-proline, is also a rich source of carbon and nitrogen for many micro-organisms. In *Sinorhizobium meliloti*, the hydroxyproline transport and utilization genes (*hyp*) are negatively regulated by the GntR-family regulator HypR, with 4-hydroxy-l-proline as an inducer (White *et al.*, 2012). Orthologues of HypR were identified in 13 taxonomic groups that mostly belong to gammaproteobacteria but also include three alphaproteobacterial and two betaproteobacterial taxa (Fig. S2). The core of reconstructed HypR regulons includes *hypR*, which is autoregulated in 32 out of 46 studied genomes, and *hypE*, *hypO*, *hypD* and *hypH* encoding enzymes involved in the conversion of hydroxyproline to *α*-ketoglutarate (Fig. 5). Operon organization of the *hyp* genes varies among the studied bacteria. Taxonomy-specific regulon members include an alternative 4-hydroxyproline epimerase (*hypY*) and two putative enzymes from the TCA cycle, malate dehydrogenase (*hypS*) and citrate isomerase (*hypX*), that are potentially involved in *α*-ketoglutarate utilization (Fig. 5). PutA, the main enzyme of the proline catabolic pathway that provides proline oxidation to 1-pyrroline-5-carboxylate, is the taxonomy-specific member of the HypR regulons in the *Shewanellaceae* and *Aeromonas* species. Ornithine can be converted to proline via ornithine cyclodeaminase (COG2423), which is predicted to be a part of the HypR regulon in *Vibrio parahaemolyticus*, *Paracoccus denitrificans* and several *Shewanella* species. Thus, HypR function probably expands onto ornithine degradation.

In *S. meliloti*, hydroxyproline is imported into the cell via the ABC-family transporter HypMNPQ, which is predicted to be a part of HypR regulons only in three other genomes and thus was classified as a taxonomy-specific regulon member. Two other predicted hydroxyproline transporters, namely HypT from the MFS family and HypABC from the TRAP family, were identified as genome-specific members of the HypR regulons. Furthermore, HypR-regulated genes encoding a putative TonB-dependent outer membrane transporter in several *Alteromonadales* and a COG531-family permease in *Erwinia amylovora* may also be involved in hydroxyproline/proline transport.

Large amounts of proline and hydroxyproline are found in the abundant protein collagen (Phang *et al.*, 2015). We identified three secreted collagenases (*colA1*, *colA2* and *colA3*) within the reconstructed HypR regulons in the *Alteromonadales* (mostly in *Shewanella* species), suggesting that the HypR regulons evolved in these species include the upstream metabolic steps in the proline/hydroxyproline utilization pathway (Fig. 5). Moreover, the reconstructed HypR regulons in the *Alteromonadales* and *Aeromonas* taxa of gammaproteobacteria include various proline (di)peptidases (PrdP, AmpP and YpdF) and proline dipeptide/tripeptide permease (PdtP), suggesting these species utilize another upstream source of proline for the catabolic pathway (Fig. 5).

### HutC regulon for histidine utilization

**Fig. 6.**
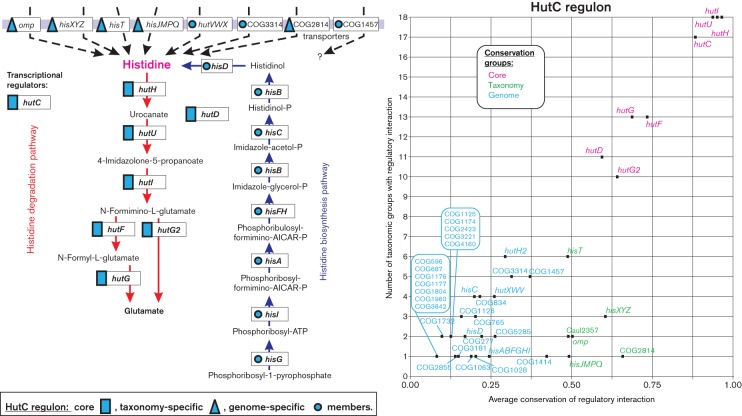
Histidine metabolism and its regulation by HutC in *Proteobacteria*.

Histidine is a well-known source of carbon, nitrogen and energy for many bacteria. The histidine degradation pathway was studied in *Klebsiella aerogenes* and *Salmonella typhimurium* and involves four reactions catalysed by HutH, HutU, HutI and HutG (re-named HutG2 in this work, belonging to the COG0010 family), whereas in *Pseudomonas* species the pathway involves an alternative HutG enzyme from the COG3741 family, as well as an additional reaction catalysed by HutF (Fig. 6) (Goldberg & Magasanik, 1975; Zhang & Rainey, 2007). The histidine utilization genes are regulated by orthologous HutC repressors in the above three gammaproteobacteria. Orthologues of HutC were identified in 113 genomes from all studied taxa of alpha-, beta- and gammaproteobacteria except the *Pasteurellales* (Fig. S2). The core of reconstructed HutC regulons includes all known histidine catabolic enzymes, including both alternative HutG enzymes and the HutD protein, which has an as yet unknown function in the pathway. Thus, the HutC regulons in the *Pseudomonadaceae*, *Enterobacteriales*, *Burkholderiales*, *Rhizobiales* and *Vibrionales* often include multiple paralogues of the histidine ammonia-lyase HutH. In addition to the *hut* genes, HutC was predicted to co-regulate the histidine biosynthesis operon in *Colwellia psychrerythraea* and *Idiomarina loihiensis* (Fig. 6).

The taxonomy- and genome-specific members of the reconstructed HutC regulons are represented by several known or putative transporters involved in histidine uptake (Fig. 6). These include three known histidine uptake systems: the HisT permease in the *Burkholderiales*, *Moraxellaceae* and *Pseudomonadaceae*, and two distinct ABC-family transporters, HisJMPQ in *Burkholderia* and HutXYZ in *Pseudomonadas* and some *Rhizobiales*. A novel histidine transporter from the ABC family (named HisXYZ) was predicted in the *Comamonadaceae*, *Ralstonia* and *Rhizobiales*. Moreover, the reconstructed regulons include a novel TonB-dependent outer membrane transporter in the *Caulobacterales* and *Sphingomonadales*, a COG2814-family permease in *Ralstonia* species and a COG3314-family transmembrane protein in *Aeromonas*, *Psychromonas* and *Marinomonas* species that are potentially involved in histidine uptake. Additional putative transporter from the COG1457 family was identified in the HutC regulons of *Burkholderia*, *Pseudomonas*, *Klebsiella* and *Acinetobacter* species, although all these species already possess the HisT permease. Therefore, there is not enough information to support assignment of histidine specificity to these novel COG1457-family transporters, which are homologous to purine and allantoine transporters.

### Taxonomy-specific regulon expansion/shrinking

In addition to the above-described five amino-acid-specific regulons and also the previously described methionine regulons (Leyn *et al.*, 2014), we observed many variations in reconstructed regulons for orthologous TFs in different lineages of *Proteobacteria,* including regulon expansion and contraction and many cases of recruiting non-orthologous TFs to control equivalent pathways. The most interesting and novel examples of the observed evolutionary changes in the reconstructed TF regulons are briefly described below.

The FabR repressor, which was previously known to control the fatty acid biosynthesis genes *fabAB* in *E. coli* and other *Enterobacteriales*, co-regulates the unsaturated fatty acid biosynthesis genes *desABC* in several lineages of gammaproteobacteria. In addition, the FabR regulon in six *Shewanella* species is expanded by the *pfaRABCD* operon encoding polyunsaturated fatty acid synthase. The fatty acid degradation pathway in *Shewanellaceae* and other gammaproteobacteria is regulated by PsrA, whereas in *Enterobacteriales* the analogous pathway is regulated by FadR. The FadR regulon in *Shewanellaceae* is contracted and retains only two operons shared with the orthologous regulon in *Enterobacteriales* and *Vibrionales* (*fadIJ* and *fadL*). The PsrA regulon in *Shewanellaceae* is expanded by several operons involved in the TCA cycle (*aceBA, sdh*, *gltA*). The biological role of PsrA regulons in two lineages of betaproteobacteria, *Ralstonia* and *Burkholderia*, is shifted to control the fatty acid biosynthesis genes, whereas the fatty acid degradation genes are predicted to be co-regulated by a novel TetR-family regulator, termed FadP, in the above two lineages, as well as in *Comamonadaceae*, which lack PsrA or FadR regulons (Kazakov *et al.*, 2009).

HexR in *Enterobacteriales* is a local regulator of glucose 6-phosphate dehydrogenase (*zwf*), whereas in other lineages of gamma proteobacteria it co-regulates *zwf*–*pgl* with genes from the Entner-Doudoroff pathway (*edd*, *eda*), glucokinase (*glk*) and pyruvate kinase (*pykA*). The HexR regulons in *Shewanellaceae* and *Vibrionales* are significantly expanded to include various other genes from the central glycolytic and fermentation pathways, glucose transport, mannitol utilization, nucleoside metabolism, glutamate biosynthesis and the glycine cleavage system (Leyn *et al.*, 2011). The pyruvate-responsive regulator PdhR, which solely controls the pyruvate dehydrogenase operon *aceEF*–*lpdA* in *Enterobacteriales*, *Vibrionales* and several other lineages, undergoes radical expansion to include genes involved in the TCA cycle (*sdhCDAB*, *gltA*, *aceAB*, *oadGAB*) and fermentation (*pflBA*, *focA*, *lldP*–*dld*) in *Shewanellaceae*.

NtrC protein is a regulator of nitrogen assimilation described in *E. coli* and *Rhodobacter capsulatus* (Reitzer, 2003; Masepohl & Hallenbeck, 2010). Orthologues of the NtrC protein were found in 19 analysed taxa. A core part of the regulon contains genes for glutamine synthetase (*glnA*), ammonium transporter (*amtB*) and nitrogen assimilation regulatory genes (*glnBK* and *ntrB*, *ntrC*). On the other hand, the NtrC regulon demonstrates taxon-specific expansions to other nitrogen metabolism genes. NtrC-dependent regulation of glutamate dehydrogenase (*gdhA*) is a specific feature of the *Moraxellaceae, Rhodobacterales* and deltaproteobacteria. Genes encoding glutamate synthase (*gltBD*) are regulated by NtrC in the *Moraxellaceae* and *Shewanellaceae*, as well as in some beta-, alpha- and deltaproteobacteria. In a number of taxa, the NtrC regulon is expanded to the genes encoding hydrogenases that act on carbon–nitrogen bonds. Thus, allophanate hydrolase (*atzF*) and agmatinase (*speB*) are regulated in the *Rhizobiales* and betaproteobacteria, respectively, whereas urease (*ureABC*) and urea ABC transporters (*uctABC* and *urtABCDEF*) are regulated in the *Alteromonadales*, *Oceanospirillales*, *Rhizobiales* and *Rhodobacterales*. In alpha- and betaproteobacteria, the NtrC regulon is expanded to genes involved in nitrogen oxide uptake and reduction, such as assimilatory reductases of nitrate (*nasAB*) and nitrite (*nasDE* and *nirA*), nitrate–nitrite antiporter (*narK*) and nitrate ABC transporter (*nrtABC*). Together, the core of the NtrC regulon includes genes necessary for inclusion of ammonia into organic compounds through glutamine synthesis, whereas the taxon-specific regulon members are necessary for generation of ammonia via metabolism of various nitrogen compounds.

### Non-orthologous TFs for *N*-acetylglucosamine utilization pathway

Three different TFs were previously found in *Proteobacteria* to control the *N*-acetylglucosamine catabolic pathway, namely NagC, NagQ and NagR (Yang *et al.*, 2006). All three regulons have similar cores consisting of two central enzymes from the *N*-acetylglucosamine pathway (*nagA* and *nagB*) and a PTS-family transporter that is involved in uptake and phosphorylation of *N*-acetylglucosamine. The taxonomic distribution of these three regulatory systems is not uniform: NagC and NagR were found in gammaproteobacteria, whereas NagQ was identified in alpha- and betaproteobacteria, as well as in some lineages of gammaproteobacteria. Interestingly, *Reinekea* sp. MED297 has two distinct regulators, where the NagQ regulon contains genes involved in the sugar catabolic pathway (*nagKAB*) and chitin degradation (*cdxA, chiA*), whereas NagC controls genes encoding an *N*-acetylglucosamine-specific PTS transporter and chemotaxis proteins. Furthermore, in two *Xanthomonas* species, we found both NagQ and NagR, where NagQ regulates the *N*-acetylglucosamine catabolic and transport genes (*nagAB* and *nagP*), while NagR regulons include the *N*-acetylglucosamine kinase and TonB-dependent outer membrane receptors that might be involved in sugar transport across the outer memberane. Overall, all three *N*-acetylglucosamine-specific TF regulons are expanded in many genomes to include various chitin utilization genes. Also, a significant expansion of the NagC regulon was observed in the *Vibrionales*, where it includes the central glycolytic (*gapA*, *gapB*, *fbaA*, *pgk*, *eno*) and glycogen biosynthesis (*glgAC*) genes.

### Two distinct TF regulons for biotin biosynthesis pathway

Two distinct TFs, BirA and BioQ, control the biotin/vitamin B7 biosynthesis pathway in *Proteobacteria*. BirA was previously studied in detail in *E. coli* (Beckett, 2005). It is a protein that functions both as a transcriptional repressor and as a biotin-protein ligase, which covalently links biotin to biotin-dependent enzymes. BirA enzymes are ubiquitous in micro-organisms, while the N-terminal DNA-binding domain can be only found in a subset of BirA proteins from a broad number of *Proteobacteria*, *Firmicutes* and several other lineages of *Bacteria* and *Archaea*. Among the studied *Proteobacteria*, BirA repressors and regulons were found in the gamm- and delta subdivisions. The most conserved part of BirA regulons consists of the biotin biosynthesis genes (*bioABCDF*). The BirA regulons in *Desulfovibrionales* are expanded to include the fatty acid biosynthesis genes (*fabF*, *fabH*, *acpP*), which are involved in the same pathway with the biotin-dependent acetyl-CoA carboxylase. The BirA proteins from beta- and alphaproteobacteria have lost their DNA-binding domains, and apparently the BirA regulons do not exist in these species.

Previous comparative genomics analysis of biotin pathway genes has identified a novel GntR-family TF in alphaproteobacteria from the *Rhizobiales* and *Rhodobacteriales* lineages, termed BioR, which was predicted to control the biotin metabolism genes (Rodionov & Gelfand, 2006). The reconstructed BioR regulons include the biotin biosynthesis genes (*bioABDF*, *bioCG*, *bioZ*), as well as a novel ECF-family transporter for biotin (*bioYMN*) (Hebbeln *et al.*, 2007). The BioR regulon was later experimentally validated in *Brucella melitensis* and *Paracoccus denitrificans* (Feng *et al.*, 2013, Feng *et al.*, 2015).

### Identification of a novel TF regulon involved in NAD biosynthesis

In the *Enterobacteriales,* the NAD cofactor metabolism genes are controlled by the NadR regulator, which is absent in all other lineages of *Proteobacteria*. The most conserved part of the NadR regulon is the *nadA*–*pnuC* operon that encodes a *de novo* NAD biosynthesis enzyme and a ribosyl nicotinamide transporter. In several enterobacterial genomes, NadR controls additional NAD biosynthesis and nicotinate/vitamin B3 salvage genes (*nadB*, *pncB*, *niaP*, *nadR*).

In alphaproteobacteria and several species of beta- and gammaproteobacteria we found a novel TF, termed NadQ, which presumably controls the NAD biosynthesis genes. Proteins from the NadQ orthologous group belong to an as yet undescribed protein family (COG4111) that has a characteristic C-terminal DNA-binding domain with a helix-turn-helix (HTH) motif, which is homologous to another regulator of NAD metabolism, NrtR (Rodionov *et al.*, 2008). However, the N-terminal effector binding domain of NadQ is unique as it is not similar to the ADP ribose-binding domain of NrtR. Palindromic DNA binding motifs for NadQ identified in seven lineages of *Proteobacteria* are characterized by the common consensus sequence ttATRCTCannntGAGYATaa, where R and Y stand for purines or pyrimidines, respectively. The *nadQ* genes are often clustered on the chromosome with the *de novo* NAD biosynthesis genes (*nadABC*). Thus, the core of reconstructed NadQ regulons in proteobacteria includes these central NAD biosynthesis genes. In the *Rhodobacterales* and *Caulobacterales*, the NadQ regulon is expanded to include the lower NAD biosynthesis pathway genes (*nadE* and *nadD*). The effector molecule for the novel NadQ regulator has yet to be determined experimentally but the regulon content suggests that it may be either NAD itself or one of the pathway intermediates.

## Conclusions

We used the comparative genomics approach for reconstruction of regulatory networks for amino acid and other central metabolic pathways that are controlled by specific groups of TFs. The results of this study demonstrate considerable variability of the TF regulons for the central metabolic pathways in Gram-negative bacteria from the phylum *Proteobacteria*. The core members of the characterized TF regulons are involved in arginine biosynthesis (ArgR), biotin biosynthesis (BirA), fatty acid biosynthesis (FabR) and degradation (FadR, FadP, PsrA), glycolate, lactate and pyruvate utilization (GlcC, LldR, PdhR), central carbohydrate metabolism (HexR), histidine and hydroxyproline/proline utilization (HutC, HypR), branched‐chain amino acid degradation (LiuR, LiuQ), methionine metabolism (MetJ, MetR, SahR), nitrogen assimilation (NtrC), deoxyribonucleotide biosynthesis (NrdR), *N*-acetylglucosamine utilization (NagC, NagQ, NagR), pyrimidine degradation (RutR), tyrosine and phenylalanine metabolism (TyrR), and tryptophan biosynthesis (TrpR). Large-scale phylogenomic and comparative genomics analyses of these TFs reveal numerous examples of various evolutionary processes for regulators and their regulons at the levels both of a taxonomic group/class of bacteria and of an individual genome. These predicted evolutionary processes can be classified into three distinct types: (i) 'regulon expansion' in certain taxa compared with other lineages that can range from additions of several regulon members to large-scale shifts in the regulated metabolic pathways (e.g. PdhR, TyrR and TrpR regulons in *Shewanella* species); (ii) 'fuzzy regulons' when a regulon contains a small conserved core and a large periphery of taxon- and genome-specific genes (e.g. ArgR, HexR and NtrC regulons); and (iii) 'regulon loss or acquisition' when an entire regulon (including a TF and all its TFBSs and target genes) is absent or present only in specific genomes within a taxonomic group of bacteria (e.g. GlcC and HypR, which are present in individual species of *Enterobacteria,* or NagR, which was found in all but one *Shewanella* species). The most conserved regulatory interactions were identified within TF regulons that are involved in the control of certain essential biosynthetic pathways (e.g. BirA, NrdR and FabR). Overall, this study provides new insights into the evolutionary mechanisms that shape transcriptional regulatory networks in *Bacteria*.

## Supplementary Data

3162 Supplementary File 1
