# Supplementary material for: Comparative genomics and evolution of transcriptional regulons in Proteobacteria
Source: Microb Genom. 2016 Jul 11;2(7):e000061. doi: 10.1099/mgen.0.000061 (PMC5343134; doi:10.1099/mgen.0.000061)
Supplement: Supplementary file 1 [file mgen-02-61-s001.pdf]

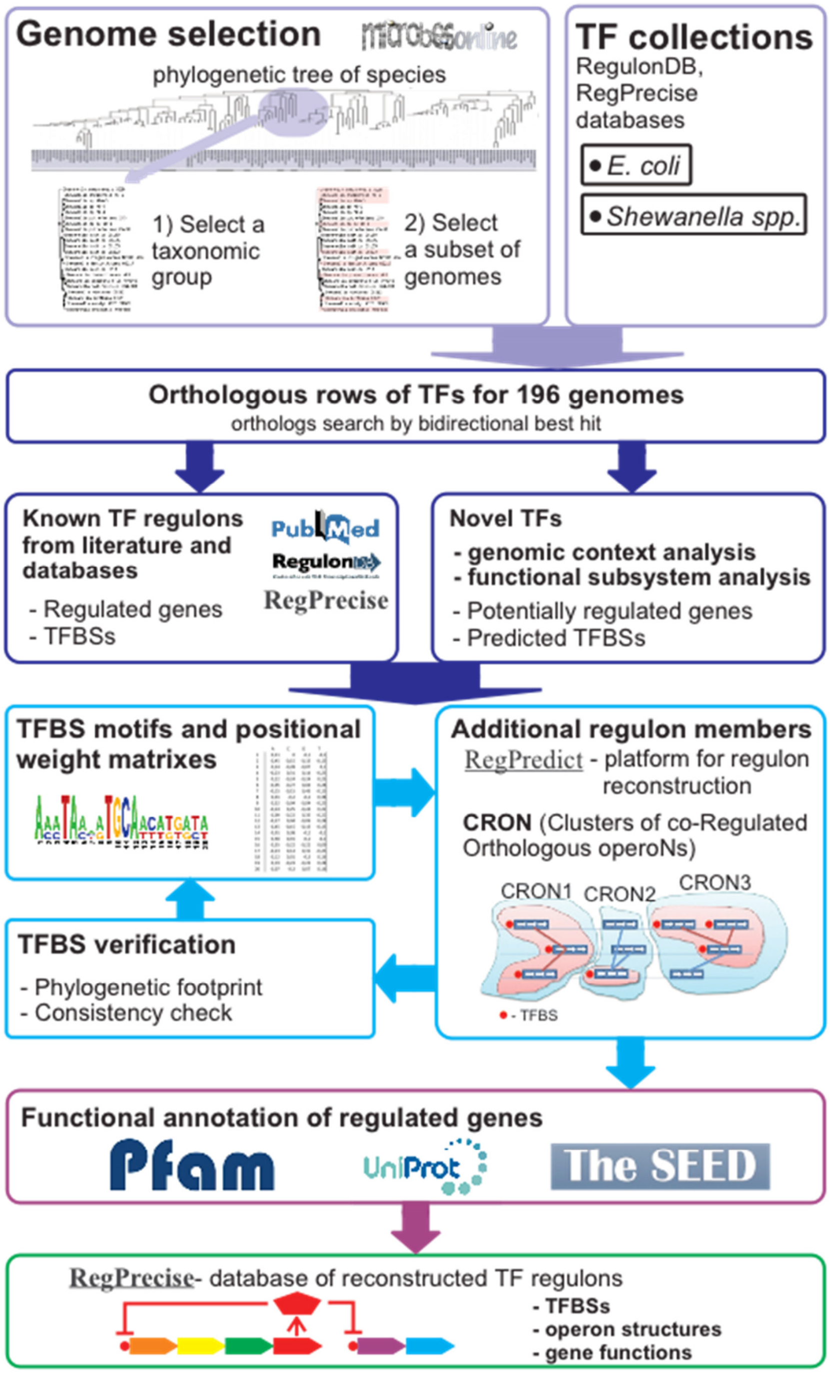

Figure S1. Bioinformatics workflow used for reconstruction of TF regulons.

For regulon reconstruction we started from collections of known TFs in *Escherichia coli* and *Shewanella* species and identification of their orthologs. Collections of TFs were obtained from RegulonDB (Gama-Castro, et al. 2016) for *E. coli* and the RegPrecise (Novichkov et al., 2013) for *Shewanella* spp. For subsequent reconstruction steps we used genomes from MicrobesOnline (Dehal, et al. 2010) database because the RegPredict platform for reconstruction of transcription regulation is linked with genomes in MicrobesOnline. In each taxonomic group, from 4 to 16 representative genomes were selected based on the MicrobesOnline species tree. Closely related strains were eliminated from our analysis to

avoid skews in the consistency check approach and to simplify the simultaneous analysis in the RegPredict web server. At that we preferably selected most well studied genome representative in each set of closely-related genomes. Next we searched for orthologous TFs in the selected genomes using the bidirectional best hits approach and protein BLAST server at NCBI (Altschul, et al. 1997).

For regulon reconstruction in each group of genomes possessing TF orthologs we used standard comparative genomics approach (Rodionov 2007) that consists of the next steps:

1. Obtain training set of potential TFBS;
2. Build positional weight matrix (PWM);
3. Whole-genomic search for additional TFBSs and regulon members;
4. False positive filtering;
5. PWM refinement and continue from step 2.

For collection of training sets we used two strategies. (i) For studied known regulons we collected upstream regions of known to be regulated genes with attention for more precise information about location of TFBSs (as electrophoretic mobility shift assay or DNase footprinting assay). (ii) For novel TF regulons, we used genomic context analysis where we predicted regulation of neighborhood genes by their conservative co-localization in one locus mapped to phylogenetic tree of TF. Another approach is functional analysis based on assumption that genes from one metabolic pathway or one process should be regulated simultaneously. Based on this approach we taken upstreams of genes from one process. Association of TF with regulation was made by conservative co-localization of TF gene with genes from this pathway.

Collected upstream regions were used to identify a common DNA motif using the Discover Profiles tool in the RegPredict platform (Novichkov et al., 2010). We searched for DNA motifs either palindromic or tandem repeat symmetry. Sequences of identified DNA motif sites were used to build PWM. The constructed PWMs were further used to search for additional potential TFBSs across upstreams of all genes in genomes using the RegPredict server. Typically we searched the regions beginning 400 nt upstream to and ending 50 nt downstream to the translational start of each gene. Typical threshold for site search procedure was selected as 10% less of the lowest site score from the training set.

The whole genomic searches in RegPredict result in construction of a set of CRONs (Clusters of co-Regulated Orthologous operoNs). Each CRONs in RegPredict was built by the following algorithm: 1) PWM found potential TFBSs above threshold; 2) operon predicted by taking gene with potential TFBS as the first gene of operon and prolong operon to all genes with the same direction and intergenic distance less than 200 nt; 3) identification of orthologs and paralogs for each gene in this operon based on Orthologous Groups in MicrobesOnline database; 4) steps 2 and 3 repeated until convergence. Automatic construction of CRONs and manual curation of the obtained CRONs in the RegPredict server allowed us to filter out false positive site predictions by utilizing the consistency check approach. The consistency check approach is based on the assumption that true sites are conserved in evolution. It should be noted that the cases of operon gene content rearrangement are also taken into consideration in the course of CRON analysis and curation. On next step, the identified true positive TFBSs were added to refine PWM and further repeat the genomic site searches.

At the final step of the manual regulon annotation, gene functions are assigned using the existing gene annotations in Genbank and SEED databases (Overbeek, et al. 2005), annotations of homologous proteins in SwissProt / UniProt database (UniProt 2015) and analysis of Pfam domains (Finn, et al. 2016). All reconstructed regulons were finally deposited in the latest release of the RegPecise database (http://regprecise.lbl.gov) (Novichkov, et al. 2013).

		γ-proteobacteria										β-proteobacteria				α-proteobacteria					δ-proteob.			
TF	*Escherichia coli*	Enterobacteriales (12)	Vibrionales (10)	Pasteurellales (9)	Shewanellaceae (16)	Other Alteromonadales (9)	Psychromonadaceae/ Aeromonadales (6)	Oceanospirillales/ Alteromonadales (12)	Pseudomonadaceae (8)	Moraxellaceae (4)	Xanthomonadales (4)	Ralstonia (6)	Burkholderia (8)	Comamonadaceae (11)	Other various β-proteobacteria (12)	Rhizobiales (15)	Rhodobacterales (15)	Rhodospirillales (9)	Sphingomonadales (7)	Caulobacterales (4)	Desulfovibrionales (10)	Desulfuromonadales (9)	TOTAL reguloGs	TOTAL reguloNs
ArgR	+	12	10	9	16	9	6	0	0	0	0	0	0	0	0	0	0	0	0	0	0	0	6	62
BioR*	-	0	0	0	0	0	0	0	0	0	0	0	0	0	0	7	6	0	0	0	0	0	2	13
BirA	+	12	10	0	16	9	6	11	8	0	3	0	0	0	8	0	0	0	0	0	3	8	11	94
FabR	+	12	10	8	16	9	4	6	8	2	3	0	0	0	0	0	0	0	0	0	0	0	10	78
FadP*	-	0	0	0	0	0	0	0	0	0	0	6	8	11	0	0	0	0	0	0	0	0	3	25
FadR	+	12	10	9	16	9	5	0	0	0	0	0	0	0	0	0	0	0	0	0	0	0	6	61
GlcC	+	1	0	0	0	0	0	3	6	0	0	0	4	0	2	6	1	0	0	0	0	0	7	23
HexR	+	11	10	0	16	5	6	6	16	0	0	6	8	9	2	0	0	0	0	0	0	0	13	95
HmgQ*	-	0	0	0	16	0	0	0	1	0	0	0	0	0	0	0	0	0	0	0	0	0	2	17
HmgR*	-	0	0	0	0	0	0	0	5	0	0	0	0	0	0	0	0	0	0	0	0	0	1	5
HmgS*	-	0	0	0	0	3	0	0	0	0	0	0	0	0	0	0	0	0	0	0	0	0	1	3
HutC	-	8	10	0	16	8	4	3	6	2	3	6	8	7	1	10	10	5	2	4	0	0	18	113
HypR	-	1	1	0	16	3	3	2	1	0	0	1	3	0	0	6	8	1	0	0	0	0	13	46
LiuQ*	-	0	0	0	0	0	0	0	0	0	0	4	8	2	0	0	0	0	0	0	0	0	3	14
LiuR	-	0	6	0	16	8	2	6	8	0	0	6	2	10	5	9	13	3	6	4	0	0	16	104
LldR	+	5	0	0	0	0	0	4	7	2	1	4	8	3	5	0	2	4	0	0	6	4	13	55
MetJ	+	12	10	9	16	9	6	0	0	0	0	0	0	0	0	0	0	0	0	0	0	0	6	62
MetR	+	12	10	8	16	4	5	12	8	0	3	6	7	5	8	0	13	0	0	0	0	0	14	117
NadQ*	-	0	0	0	0	0	0	0	0	2	0	0	0	2	1	12	2	7	0	4	0	0	7	30
NadR*	+	11	0	0	0	0	0	0	0	0	0	0	0	0	0	0	0	0	0	0	0	0	1	11
NagC	+	12	10	2	0	0	6	1	0	0	0	0	0	0	0	0	0	0	0	0	0	0	5	31
NagQ*	-	0	0	0	0	0	0	3	2	0	2	1	7	0	1	7	4	1	0	3	0	0	10	31
NagR*	-	0	0	0	15	3	0	3	0	0	4	0	0	0	0	0	0	0	0	0	0	0	4	25
NrdR	+	12	10	9	16	9	6	12	8	4	4	6	8	11	12	15	15	9	7	4	0	9	20	186
NrtR	-	1	4	0	3	0	3	2	4	2	0	0	0	4	2	2	1	0	0	0	0	0	11	28
NtrC	+	12	9	0	16	9	6	12	8	4	4	6	8	11	9	14	14	8	6	4	0	9	19	169
PdhR	+	12	10	0	16	9	6	2	0	0	0	0	0	0	0	0	0	0	0	0	0	0	6	55
PsrA	-	0	8	0	16	4	3	8	8	0	3	6	8	0	5	3	0	0	0	4	0	0	12	76
RutR	+	7	2	0	0	5	1	8	20	2	0	4	7	0	0	9	13	1	0	2	0	0	17	81
SahR*	-	0	0	0	0	0	0	9	8	0	0	0	0	0	0	10	2	7	7	4	9	6	9	62
SamR*	-	0	0	0	0	0	0	0	0	0	4	0	0	0	0	0	0	0	0	0	0	0	1	4
TrpR	+	12	10	8	16	0	2	2	0	2	1	0	0	0	0	0	0	0	0	0	0	0	8	53
TyrR	+	12	10	9	16	9	4	0	7	0	0	0	0	0	0	0	0	0	0	0	0	0	8	67
TOTAL regulogs	17	20	19	9	19	21	19	21	22	9	12	13	14	12	13	13	15	10	5	9	3	5	283	
TOTAL regulons	-	189	160	71	290	124	84	115	139	22	35	62	94	75	61	110	104	46	28	33	18	36		1896

Figure S2. Taxonomic distribution of studied TF regulons and regulogs in 21 taxonomic groups of Proteobacteria

21 TFs are present in *E. coli* and/or *Shewanella* spp. and are conserved in five or more taxonomic groups of Proteobacteria.

12 TFs that represent non-orthologous replacements of the initial set of TFs in some taxonomic groups are marked by asterisk (*).

Each cell in represents a TF regulog; each number corresponds to the number of TF regulons per regulog.

Numbers in red font highlight TFs/taxonomic groups that have multiple regulogs represented by paralogous TFs.

Figure S3. Conservation of regulatory interactions in the reconstructed regulons. Core, Taxonomy-specific and Genome-specific groups are highlighted with red, green and blue color respectively. Regulon member names are connected with respective dots by dashes. Functional belonging of regulon members is listed in table S3.

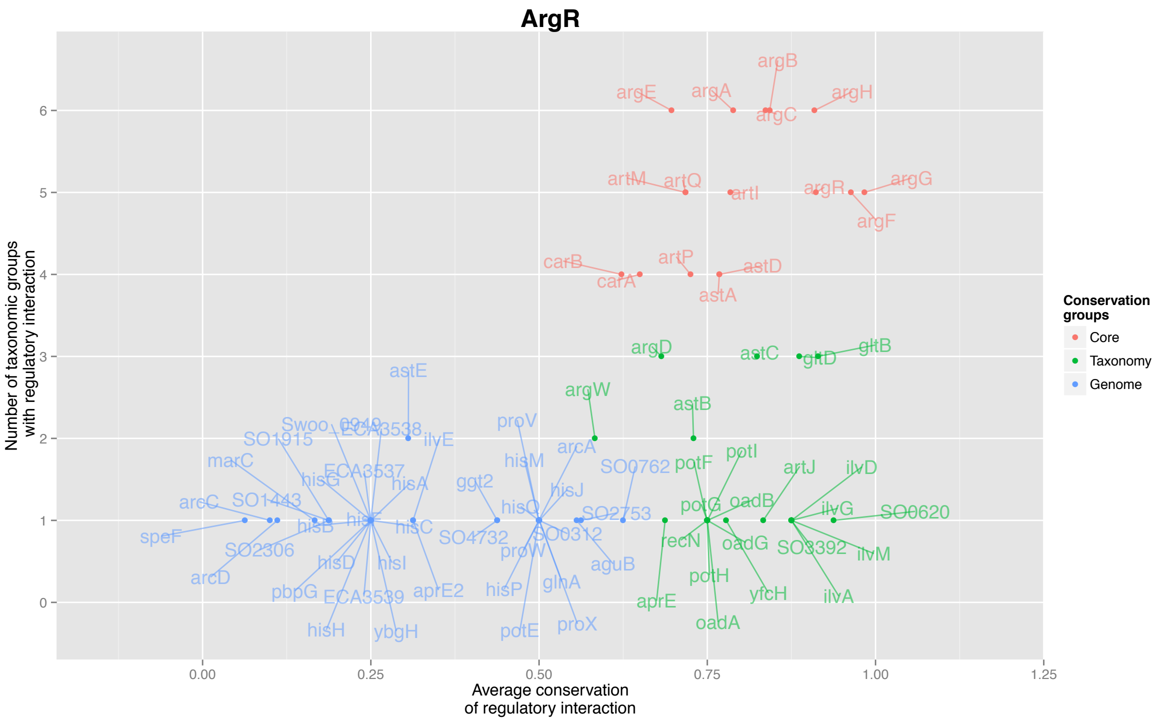
ArgR
Number of taxonomic groups with regulatory interaction
Average conservation of regulatory interaction
Conservation groups
Core
Taxonomy
Genome
argB
argE
argA
argH
argC
artM
artQ
artI
argR
argG
argF
carB
artP
astD
carA
astA
argD
astC
gltB
gltD
astE
argW
astB
proV
Swoo_0940
ECA3538
SO1915
ilvE
arcA
marC
hisM
SO0762
potF
potI
artJ
ilvD
ECA3537
hisG
hisA
ggt2
hisJ
arcC
SO1443
hisQ
potG
oadB
ilvG
SO0620
speF
hisB
hisC
SO4732
SO2753
SO0312
recN
oadG
SO3392
ilvM
SO2306
proW
arcD
hisD
hisI
aguB
potH
pbpG
ECA3539
aprE2
hisP
glnA
yfcH
ilvA
aprE
hisH
ybgH
potE
proX
oadA

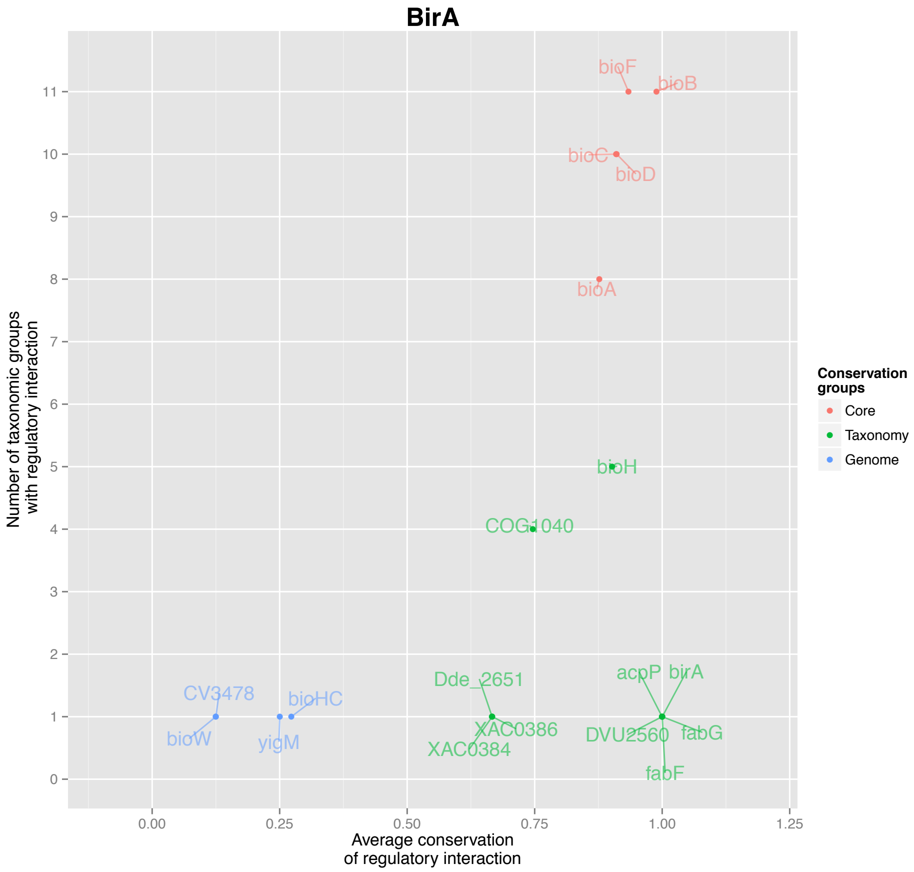
BirA
Number of taxonomic groups with regulatory interaction
Average conservation of regulatory interaction
Conservation groups
Core
Taxonomy
Genome
bioF
bioB
bioC
bioD
bioA
bioH
COG1040
CV3478
bioHC
Dde_2651
acpP
birA
XAC0386
DVU2560
fabG
bioW
yigM
XAC0384
fabF

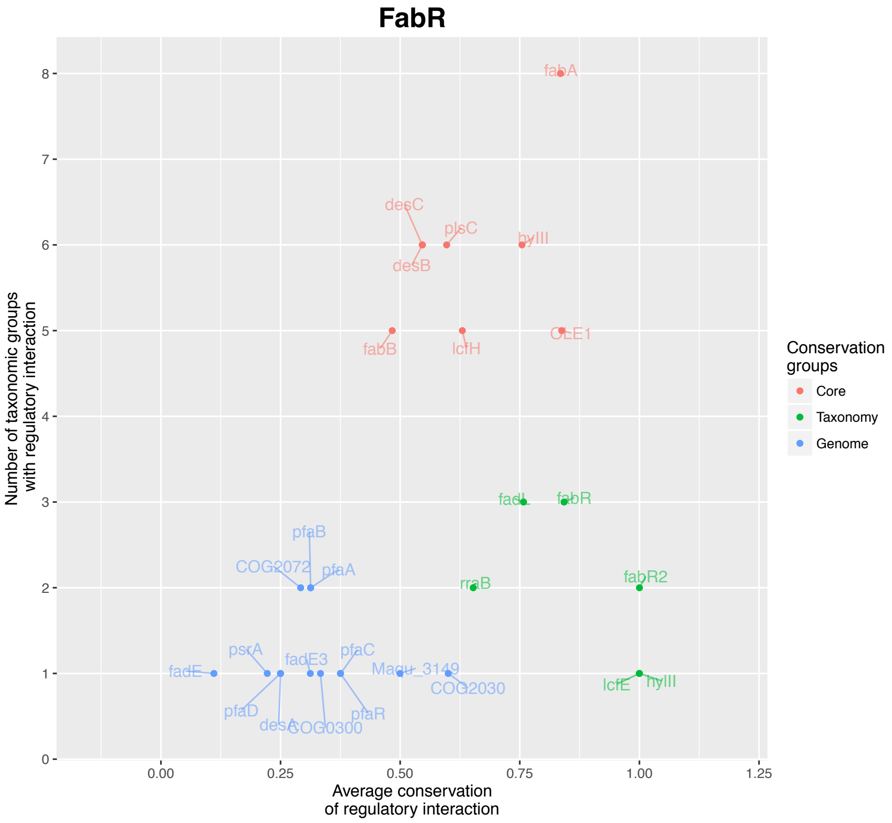
FabR
Number of taxonomic groups
with regulatory interaction
Average conservation
of regulatory interaction
Conservation
groups
Core
Taxonomy
Genome
fabA
desC
plsC
desB
fabB
OLE1
fadL
fabR
pfaB
COG2072
pfaA
rraB
fabR2
psrA
fadE
fadE3
pfaC
Macu_3149
COG2030
lcfE
hyIII
pfaD
COG0300
pfaR
0.00
0.25
0.50
0.75
1.00
1.25

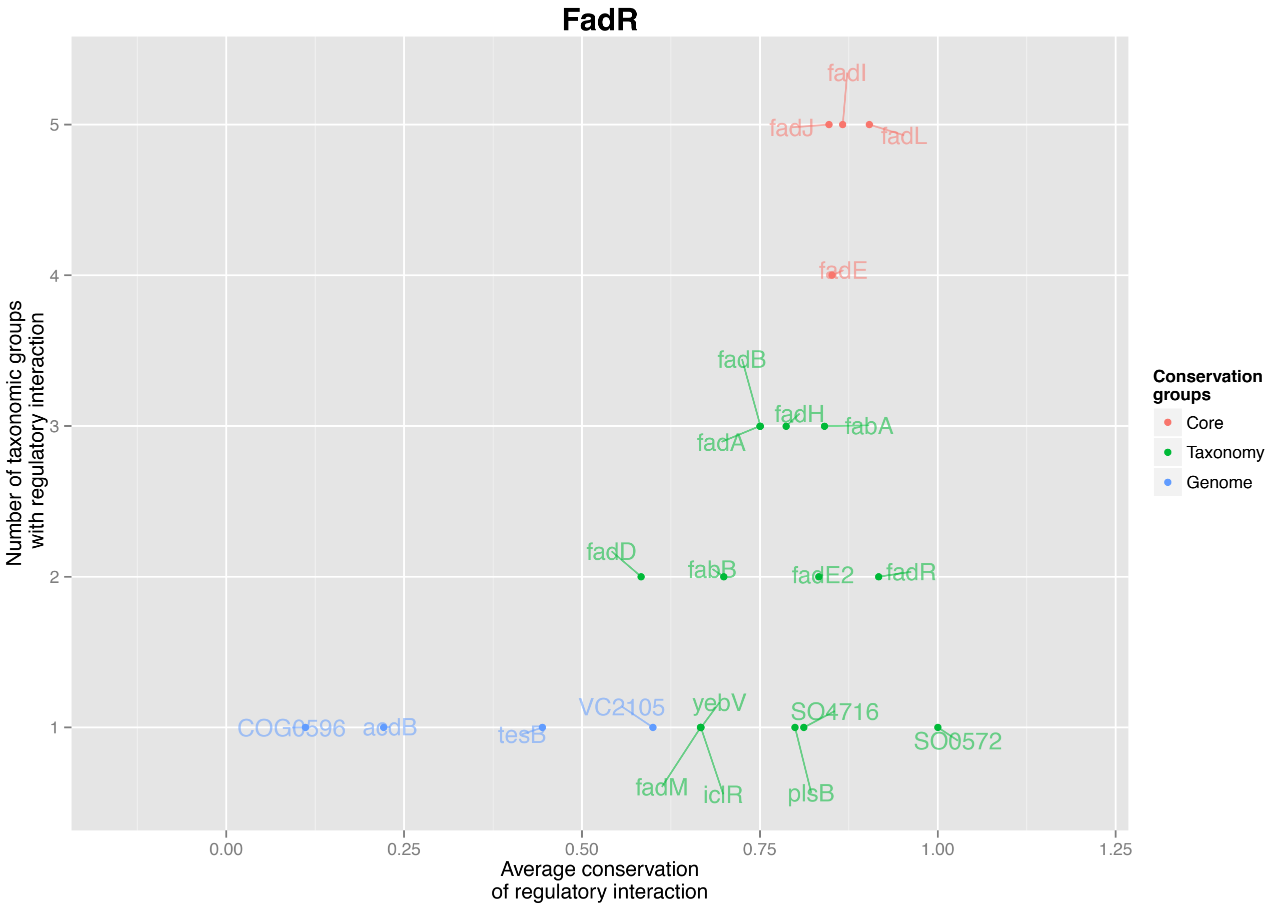
FadR
Number of taxonomic groups
with regulatory interaction
Average conservation
of regulatory interaction
Conservation
groups
Core
Taxonomy
Genome
fadI
fadJ
fadL
fadE
fadB
fadH
fabA
fadA
fadD
fabB
fadE2
fadR
VC2105
yebV
SO4716
tesB
SO0572
fadM
iclR
plsB
0.00
0.25
0.50
0.75
1.00
1.25

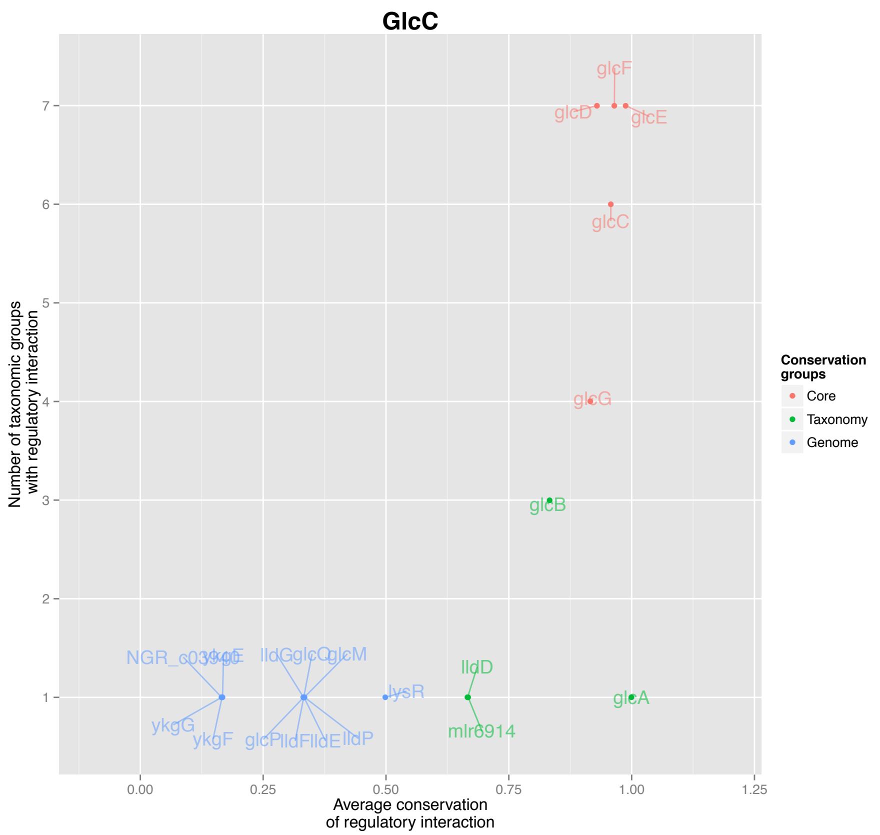
GlcC
Number of taxonomic groups
with regulatory interaction
Average conservation
of regulatory interaction
Conservation groups
Core
Taxonomy
Genome
glcF
glcD
glcE
glcC
glcG
glcB
lldD
glcA
mlr6914
lysR
ykgG
ykgF
glcP
lldF
lldE
lldP
glcM
0.00
0.25
0.50
0.75
1.00
1.25
1
2
3
4
5
6
7

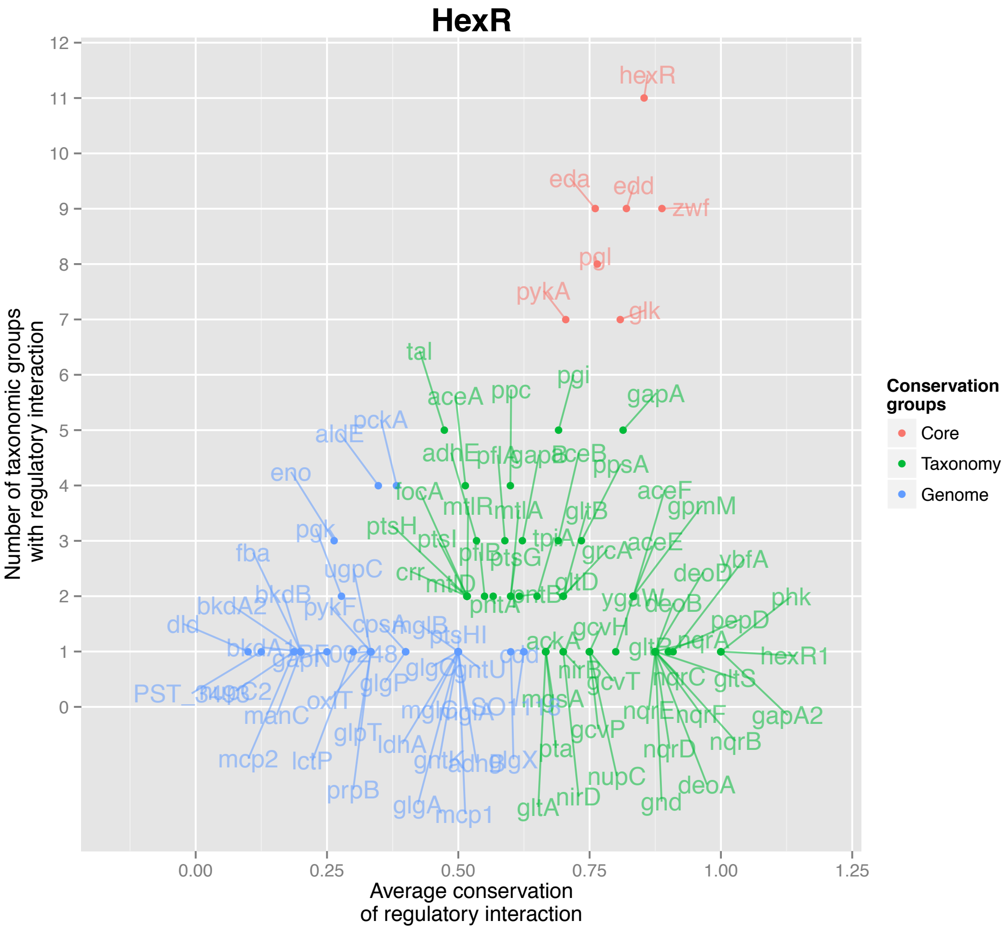
HexR
Number of taxonomic groups
with regulatory interaction
Average conservation
of regulatory interaction
Conservation groups
Core
Taxonomy
Genome
hexR
eda
edd
zwf
pgl
pykA
glk
tal
aceA
ppc
pgi
gapA
pckA
aldE
eno
pgk
fba
ugpC
bkdB
bkdA2
pykF
dld
focA
ptsH
ptsI
ptsG
crr
mtlD
tpiA
gltB
grcA
aceF
gpmM
ybfA
deoD
deoB
phk
pepD
hexR1
gltS
gapA2
nqrB
nqrD
nqrF
deoA
gnd
nupC
gltA
nirD
pta
mgsA
nirB
gcvT
gcvP
gcvH
ackA
glgX
mcp1
glgA
prpB
lctP
mcp2
manC
glpT
ldhA
oxlT
glgP
gntU
0.00
0.25
0.50
0.75
1.00
1.25
0
1
2
3
4
5
6
7
8
9
10
11
12

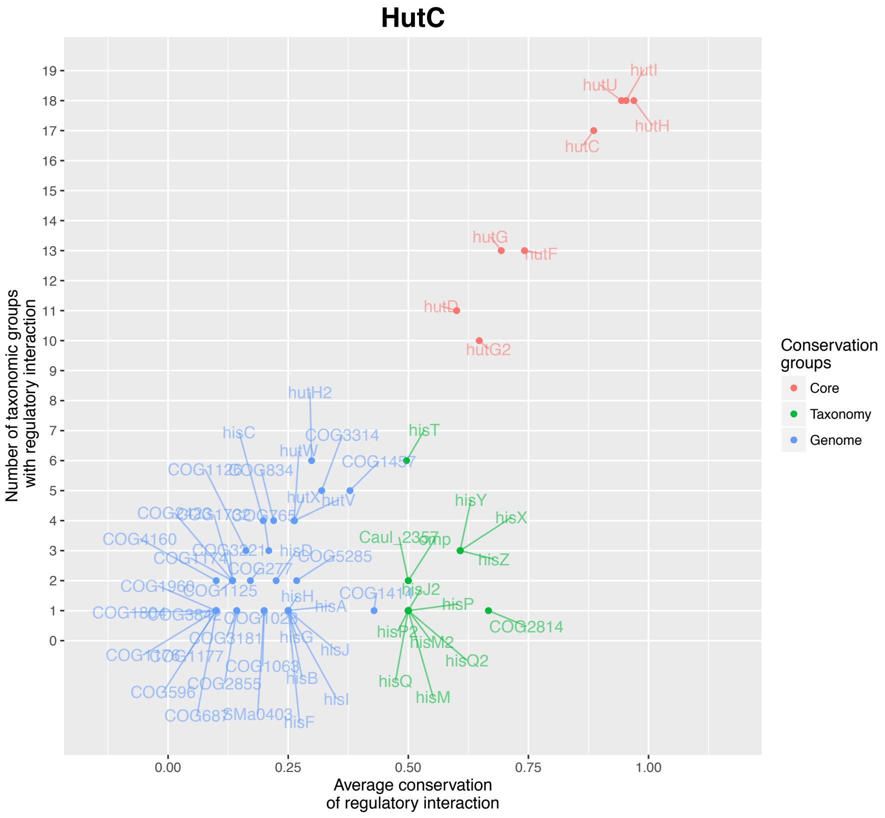
HutC
Number of taxonomic groups
with regulatory interaction
Average conservation
of regulatory interaction
Conservation
groups
Core
Taxonomy
Genome
hutI
hutU
hutH
hutC
hutG
hutF
hutD
hutG2
hutH2
hisC
COG3314
hisT
hutW
COG834
hisY
hisX
hisZ
COG4160
COG5285
COG1125
hisH
hisA
hisJ2
hisP
COG2814
COG3181
hisG
hisJ
COG1177
COG1063
hisB
hisQ2
hisQ
hisM
hisM2
COG596
COG2855
hisI
hisF
COG687
SMa0403
0.00
0.25
0.50
0.75
1.00

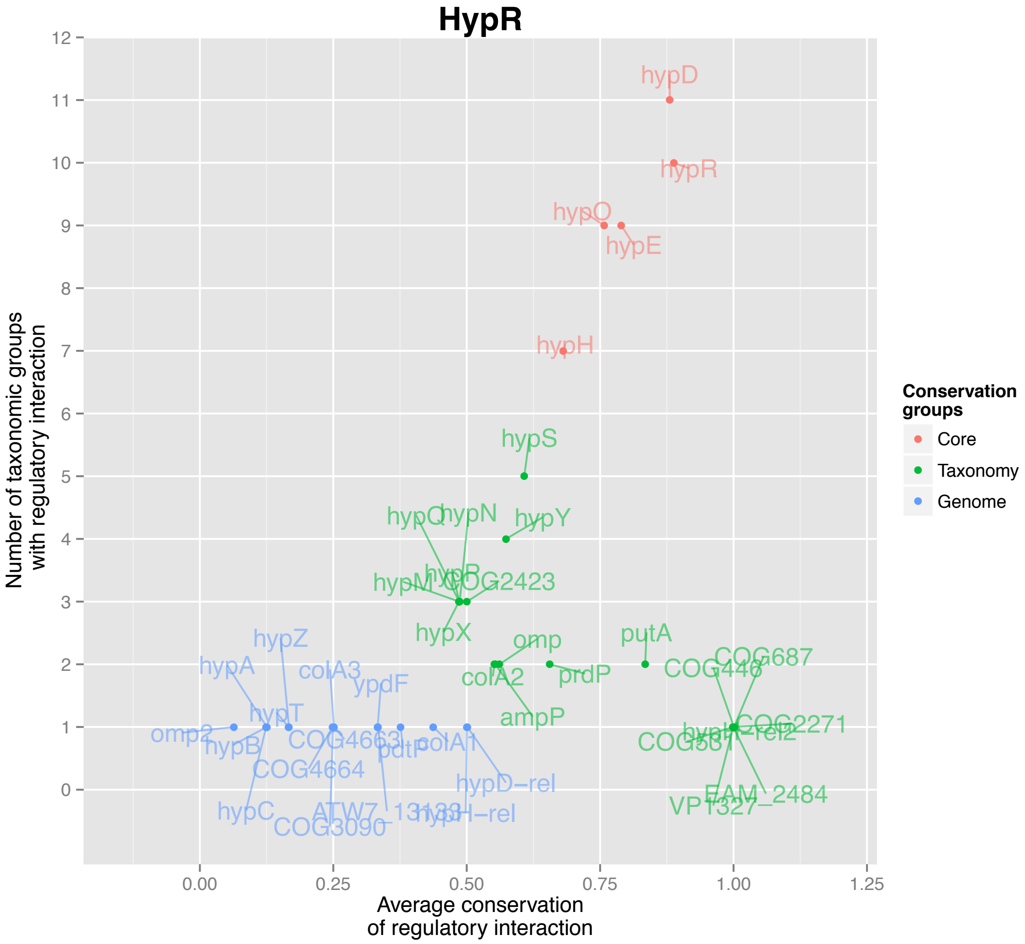
HypR
Number of taxonomic groups
with regulatory interaction
Average conservation
of regulatory interaction
Conservation
groups
Core
Taxonomy
Genome
hypD
hypR
hypO
hypE
hypH
hypS
hypN
hypY
hypM
COG2423
hypX
omp
putA
COG687
COG446
colA2
prdP
ampP
COG2271
hypZ
hypA
colA3
ypdF
hypT
omp2
hypB
COG4663
COG4664
colA1
hypD–rel
hypC
COG3090
hypH–rel
EAM_2484
VP1327
0.00
0.25
0.50
0.75
1.00
1.25

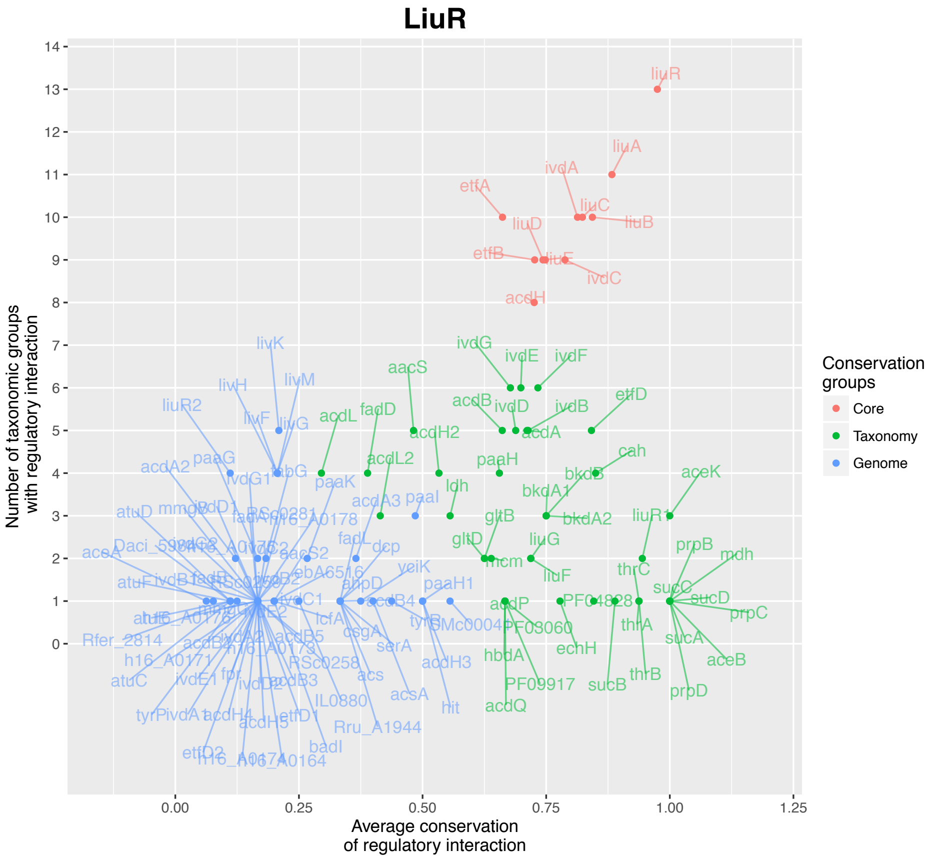
LiuR
Number of taxonomic groups with regulatory interaction
Average conservation of regulatory interaction
Conservation groups
Core
Taxonomy
Genome
liuR
liuA
ivdA
etfA
liuC
liuD
liuB
etfB
ivdC
acdH
ivdG
ivdE
ivdF
aacS
acdB
ivdD
ivdB
etfD
fadD
acdL
acdH2
acdA
cah
acdL2
paaH
bkdB
aceK
ldh
bkdA1
gltB
bkdA2
liuR1
gltD
liuG
propB
mdh
mcm
liuF
thrC
sucC
sucD
prpC
acdP
PF03060
thrA
sucA
aceB
echH
hbdA
thrB
PF09917
sucB
prpD
acdQ
livK
livH
livM
liuR2
livF
livG
acdA2
paaG
paaK
acdA3
paaI
fadL
dcp
yeiK
paaH1
acs
serA
acdH3
acsA
hit
Rru_A1944
badI
aceA
atuD
atuF
atuC
Rfer_2814
h16_A0171
tyrP
etfD2
IL0880
RSc0258
acdH5
acdH4
ivdE1
ivdC1
acdB5
acdB4
acdB3
dsgA
lcfA

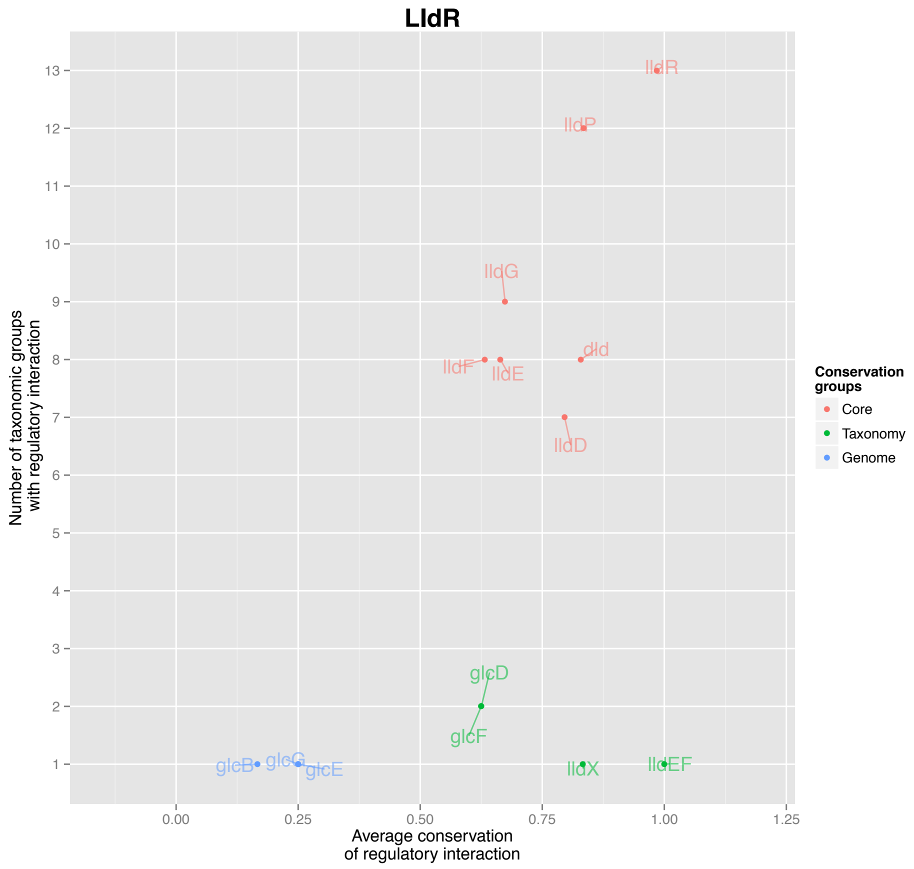
LldR
Number of taxonomic groups with regulatory interaction
Average conservation of regulatory interaction
Conservation groups
Core
Taxonomy
Genome
lldR
lldP
lldG
dld
lldF
lldE
lldD
glcD
glcF
glcB
glcG
glcE
lldX
lldEF

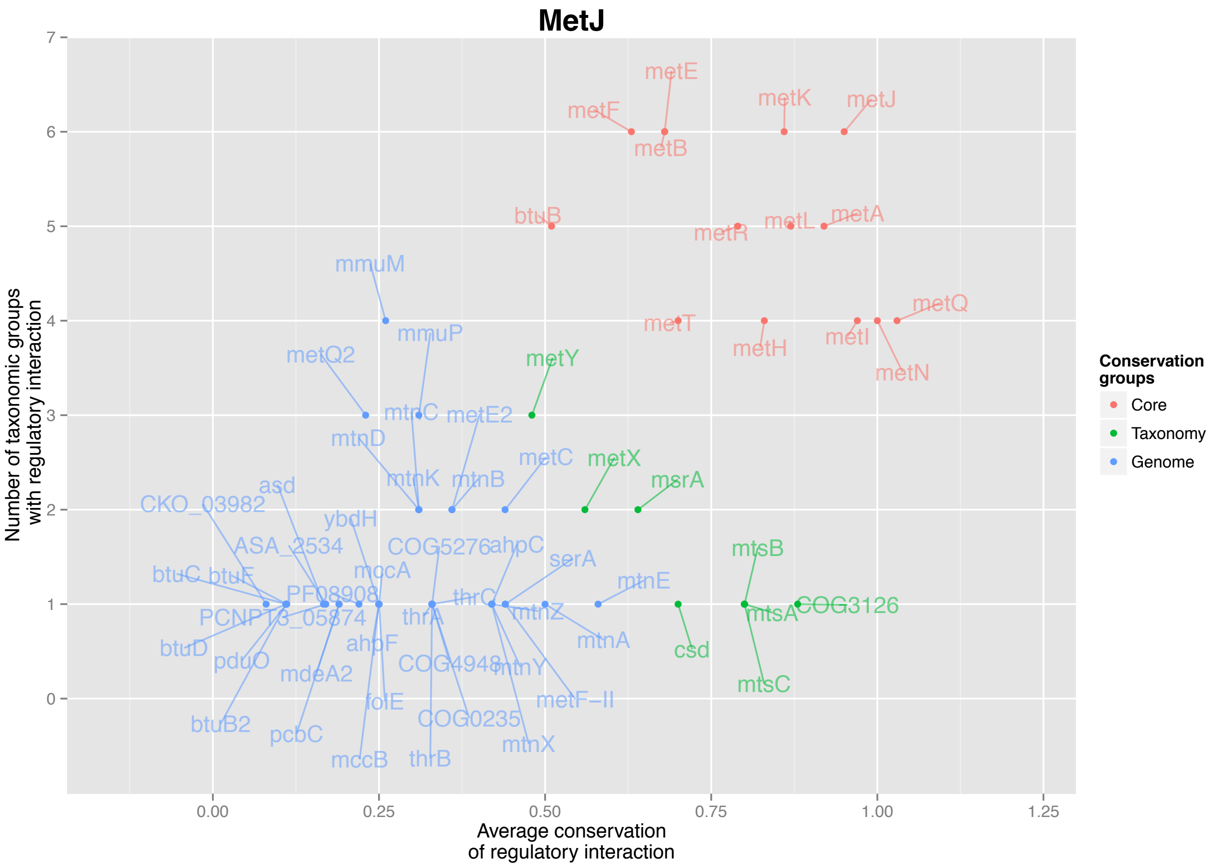
MetJ
Number of taxonomic groups with regulatory interaction
Average conservation of regulatory interaction
Conservation groups
Core
Taxonomy
Genome
metE
metK
metJ
metF
metB
btuB
metL
metA
metR
metQ
metT
metH
metI
metN
mmuM
mmuP
metQ2
metY
mtnC
metE2
mtnD
metC
metX
mtnK
mtnB
msrA
asd
CKO_03982
ybdH
ASA_2534
COG5276
ahpC
mtsB
serA
mocA
btuC
btuF
mtnE
PF08908
thrC
COG3126
PCNPT3_05874
thrA
mtnZ
mtsA
btuD
ahpF
mtnA
csd
pduO
COG4948
mtnY
mdeA2
mtsC
folE
metF–II
btuB2
COG0235
pcbC
mtnX
mccB
thrB

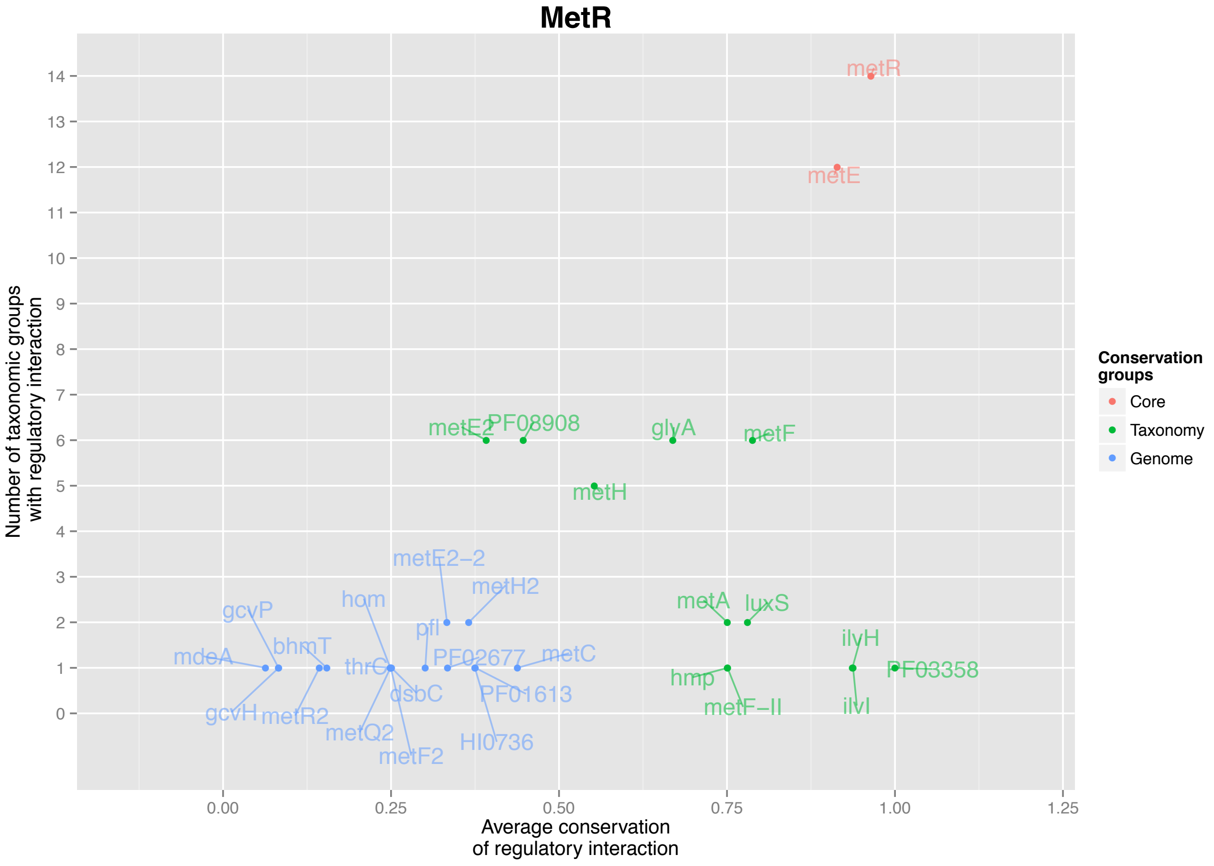
MetR
Number of taxonomic groups with regulatory interaction
Average conservation of regulatory interaction
Conservation groups
Core
Taxonomy
Genome
metR
metE
metE2
PF08908
glyA
metF
metH
metE2–2
metH2
hom
gcvP
metA
luxS
pfl
ilvH
bhmT
mdeA
thrC
PF02677
metC
hmp
PF03358
dsbC
PF01613
gcvH
metR2
metF–II
ilvI
metQ2
HI0736
metF2

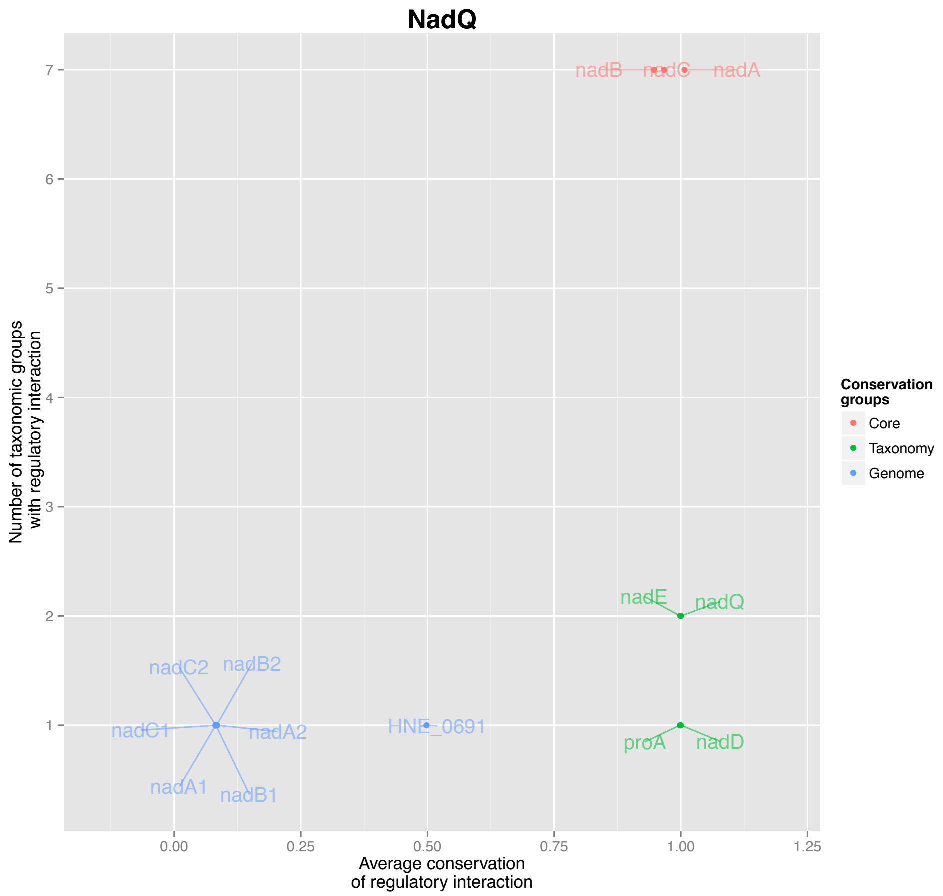
NadQ
nadB
nadC
nadA
nadE
nadQ
nadC2
nadB2
nadC1
nadA2
HNE_0691
proA
nadD
nadA1
nadB1
Number of taxonomic groups
with regulatory interaction
Average conservation
of regulatory interaction
Conservation groups
Core
Taxonomy
Genome

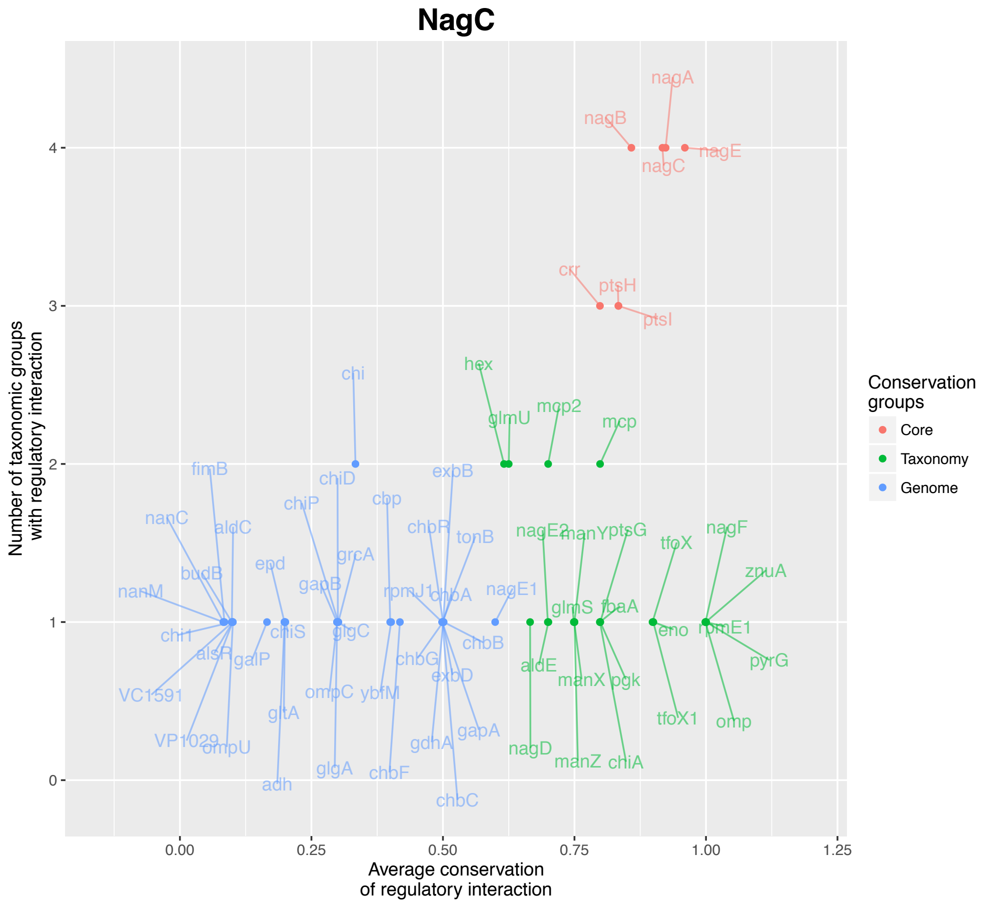
NagC
nagA
nagB
nagE
nagC
crr
ptsH
ptsI
chi
hex
mcp2
glmU
mcp
fimB
chiD
exbB
chiP
cbp
nanC
aldC
chbR
tonB
nagE2
manY
ptsG
tfoX
nagF
grcA
epd
budB
gapB
znuA
nanM
rpmJ1
chbA
nagE1
glmS
fbaA
chi1
chiS
glgC
chbB
eno
rpmE1
alsR
galP
chbG
pyrG
aldE
exbD
manX
pgk
VC1591
ompC
ybfM
gltA
tfoX1
omp
VP1029
gapA
ompU
gdhA
nagD
manZ
chiA
glgA
chbF
adh
chbC
Number of taxonomic groups
with regulatory interaction
Average conservation
of regulatory interaction
Conservation groups
Core
Taxonomy
Genome

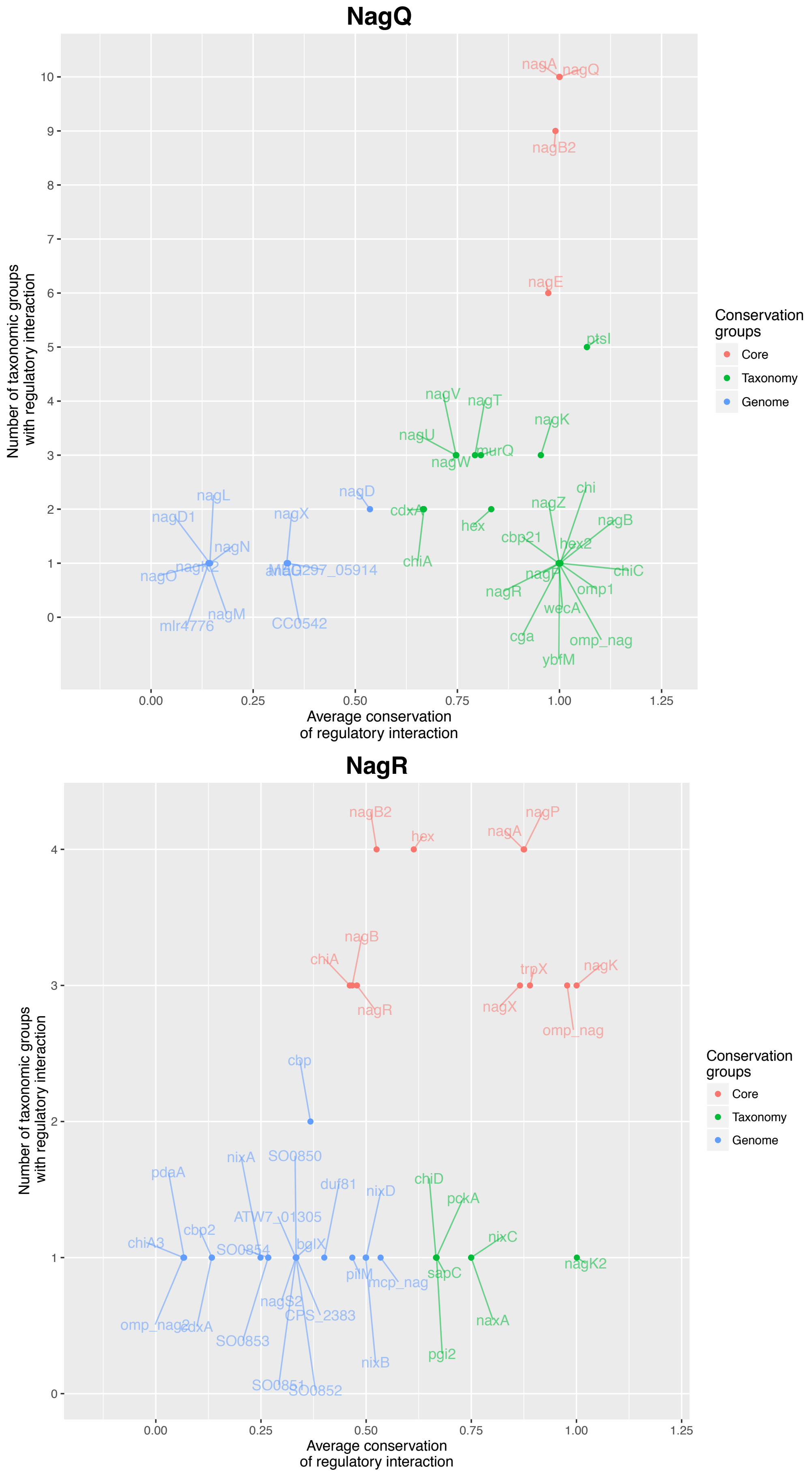
NagQ
Number of taxonomic groups
with regulatory interaction
Average conservation
of regulatory interaction
Conservation
groups
Core
Taxonomy
Genome
nagA
nagQ
nagB2
nagE
ptsI
nagV
nagT
nagK
nagU
murQ
nagW
nagL
nagD
nagD1
nagX
cdxA
chi
nagZ
nagB
hex
cbp21
hex2
nagN
chiA
chiC
nagO
MA0297_05914
nagR
omp1
wecA
nagM
mlr4776
CC0542
cga
omp_nag
ybfM
NagR
nagB2
nagP
hex
nagA
trpX
nagK
nagX
omp_nag
cbp
nixA
SO0850
pdaA
duf81
nixD
chiD
pckA
ATW7_01305
cbp2
nixC
chiA3
SO0854
bglX
pilM
mcp_nag
sapC
nagK2
nagS2
CPS_2383
omp_nag2
cdxA
naxA
SO0853
pgi2
nixB
SO0851
SO0852

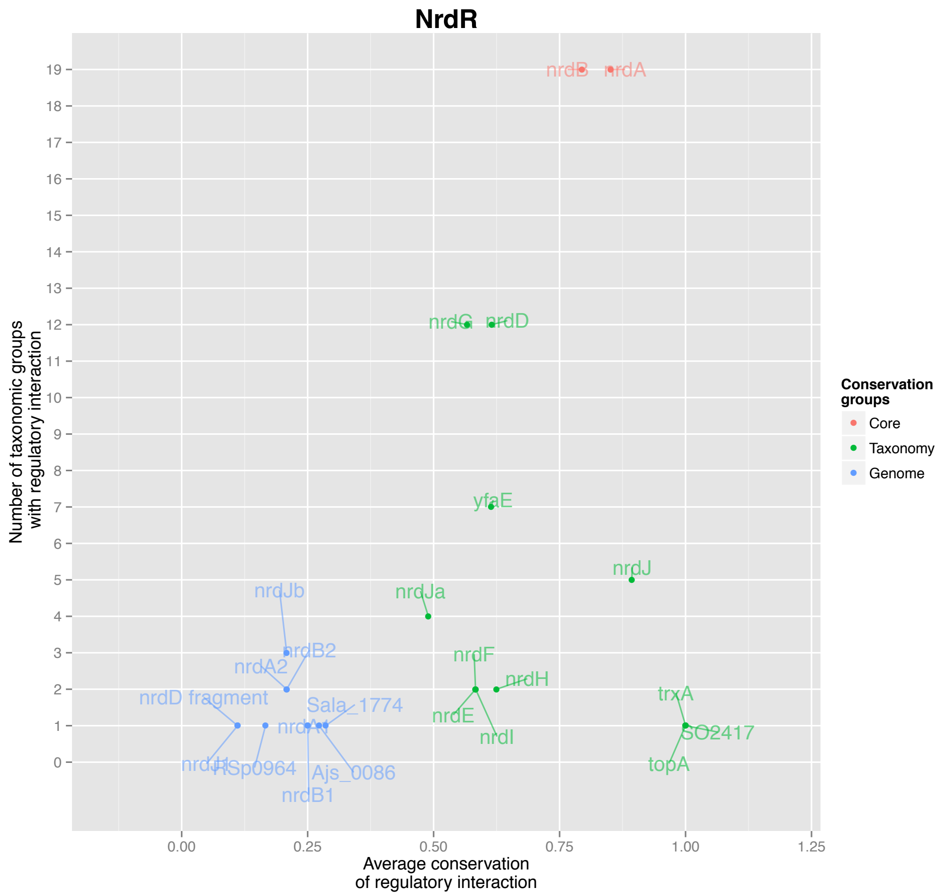
NrdR
Number of taxonomic groups
with regulatory interaction
Average conservation
of regulatory interaction
0.00
0.25
0.50
0.75
1.00
1.25
0
1
2
3
4
5
6
7
8
9
10
11
12
13
14
15
16
17
18
19
nrdB
nrdA
nrdG
nrdD
yfaE
nrdJ
nrdJb
nrdJa
nrdB2
nrdA2
nrdF
nrdH
nrdD fragment
Sala_1774
nrdE
nrdI
trxA
SO2417
topA
HSp0964
Ajs_0086
nrdB1
Conservation
groups
Core
Taxonomy
Genome

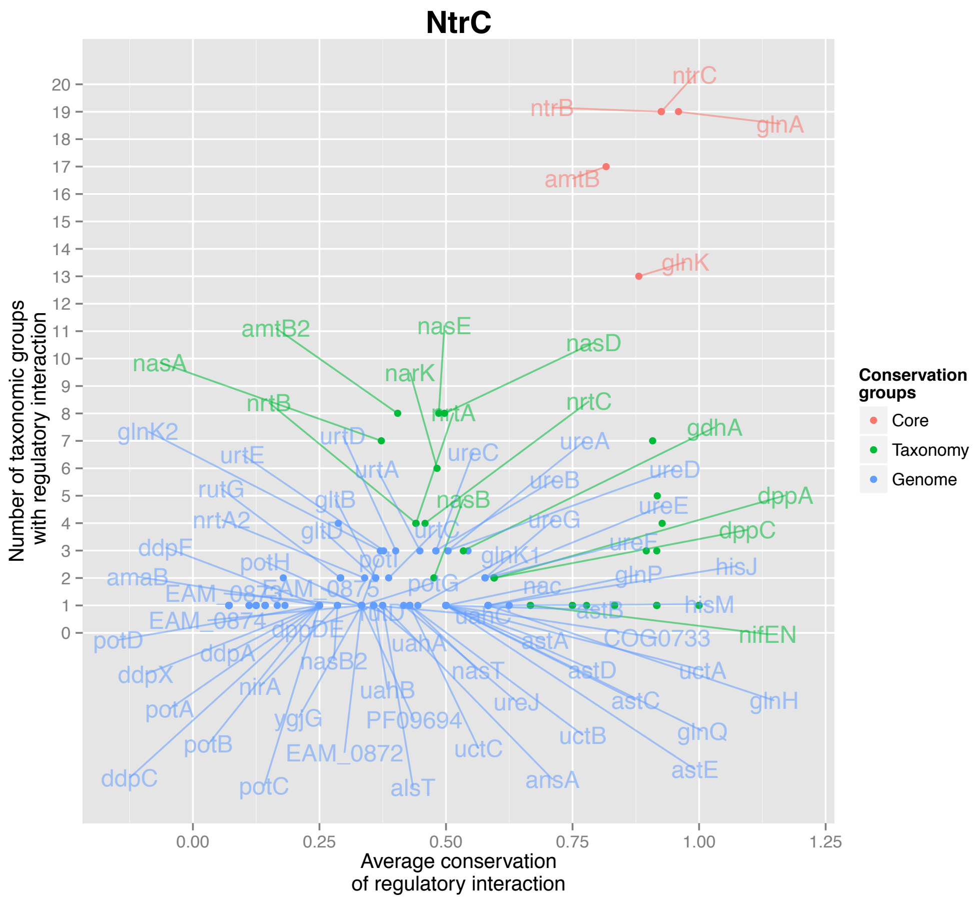
NtrC
Number of taxonomic groups
with regulatory interaction
Average conservation
of regulatory interaction
0.00
0.25
0.50
0.75
1.00
1.25
0
1
2
3
4
5
6
7
8
9
10
11
12
13
14
15
16
17
18
19
20
ntrC
ntrB
glnA
amtB
glnK
amtB2
nasE
nasD
nasA
narK
nrtB
nrtA
nrtC
glnK2
urtD
gdhA
urtE
ureA
urtA
ureC
ureD
rutG
gltB
ureB
dppA
nrtA2
nasB
ureE
gltD
ureG
ddpF
urtC
dppC
potI
ureF
potH
glnK1
hisJ
amaB
glnP
EAM_0873
EAM_0875
potG
nac
hisM
EAM_0874
dppDE
uahC
astB
potD
COG0733
nifEN
ddpA
uahA
nasB2
ddpX
nasT
astD
uctA
nirA
potA
uahB
ureJ
astC
glnH
ygjG
PF09694
glnQ
potB
uctC
uctB
EAM_0872
astE
ddpC
ansA
potC
alsT
Conservation
groups
Core
Taxonomy
Genome

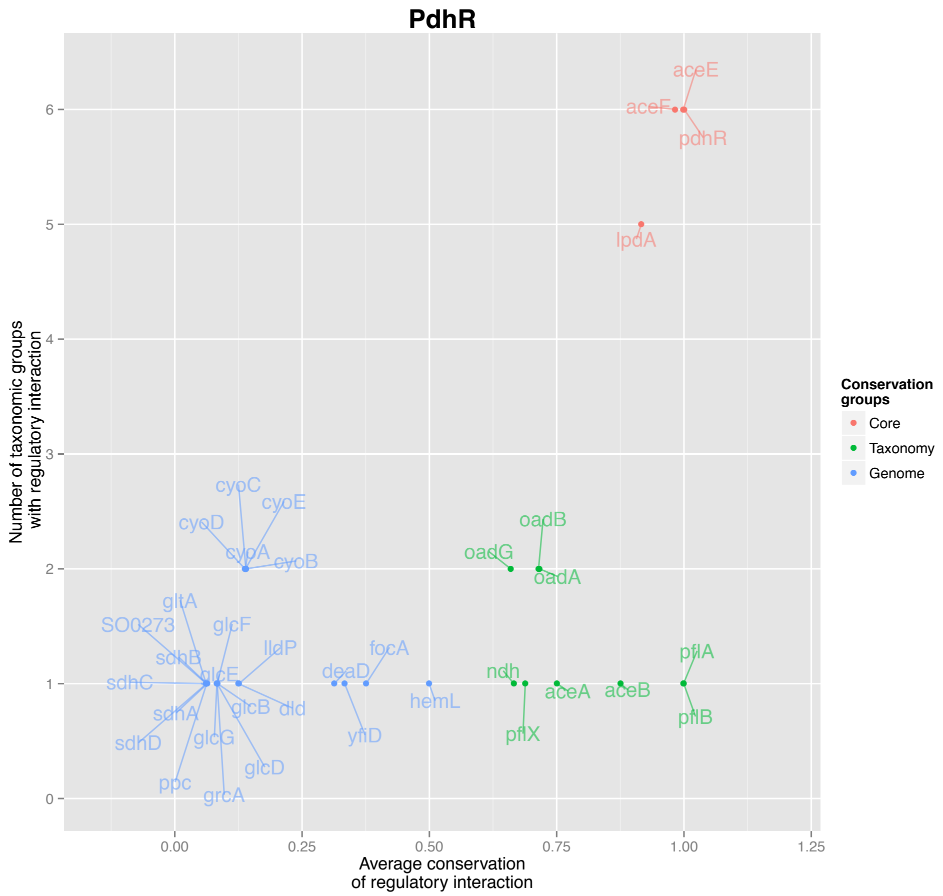
PdhR
Number of taxonomic groups with regulatory interaction
Average conservation of regulatory interaction
Conservation groups
Core
Taxonomy
Genome
aceE
aceF
pdhR
lpdA
cyoC
cyoE
cyoD
cyoA
cyoB
oadB
oadG
oadA
gltA
SO0273
glcF
sdhB
lldP
focA
glcE
deaD
ndh
pflA
sdhC
hemL
aceA
aceB
sdhA
glcB
dld
pflB
sdhD
glcG
yfiD
pflX
glcD
ppc
grcA
0.00
0.25
0.50
0.75
1.00
1.25

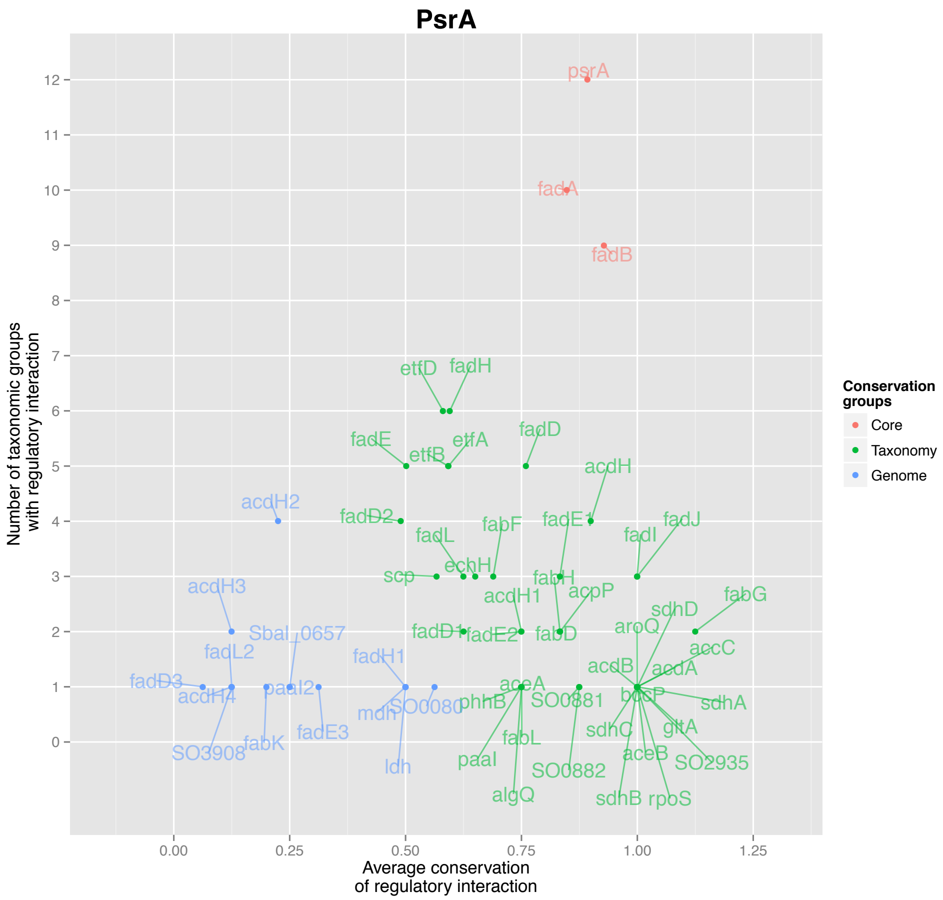
PsrA
Number of taxonomic groups with regulatory interaction
Average conservation of regulatory interaction
Conservation groups
Core
Taxonomy
Genome
psrA
fadA
fadB
etfD
fadH
fadE
etfB
etfA
fadD
acdH
acdH2
fadD2
fabF
fadE1
fadJ
fadL
fadI
scp
echH
fabH
acdH3
acdH1
acpP
fabG
sdhD
Sbal_0657
fadD1
fadE2
fabD
aroQ
fadL2
fadH1
accC
acdB
acdA
fadD3
paaI2
aceA
acdH4
SO0881
bccP
sdhA
mdh
SO0080
phhB
fadE3
sdhC
gltA
fabK
fabL
SO3908
aceB
SO2935
paaI
ldh
SO0882
algQ
sdhB
rpoS
0.00
0.25
0.50
0.75
1.00
1.25

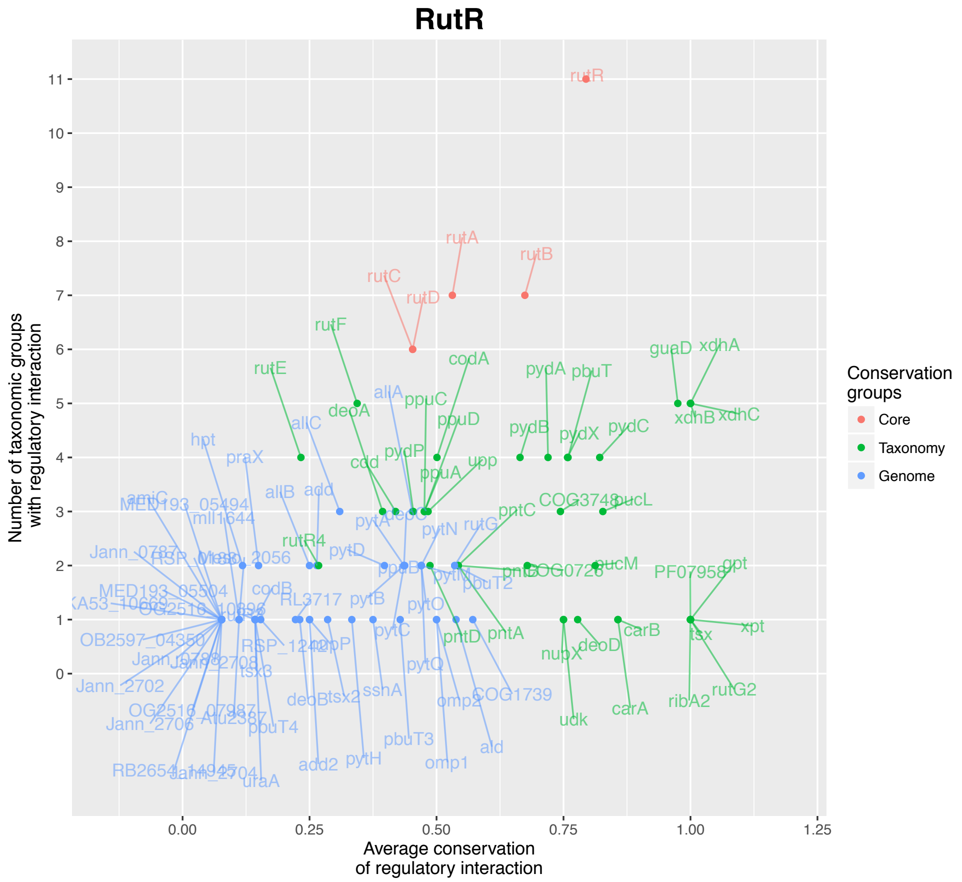
RutR
Number of taxonomic groups
with regulatory interaction
Average conservation
of regulatory interaction
Conservation
groups
Core
Taxonomy
Genome
rutR
rutA
rutB
rutC
rutD
rutF
rutE
codA
pydA
pbuT
guaD
xdhA
xdhB
xdhC
allA
ppuC
deoA
allC
ppuD
pydB
pydX
pydC
hpt
praX
pydP
cdd
upp
ppuA
allB
add
COG3748
pucL
pntC
rutR4
pytD
pytN
rutG
pucM
PF07958
gpt
COG0726
pbuT2
codB
RL3717
pytB
pytO
pytC
pntD
pntA
carB
tsx
xpt
deoD
nupX
Jann_2702
sshA
pytQ
COG1739
omp2
udk
carA
ribA2
rutG2
pbuT4
pbuT3
ald
add2
pytH
omp1
uraA
0.00
0.25
0.50
0.75
1.00
1.25

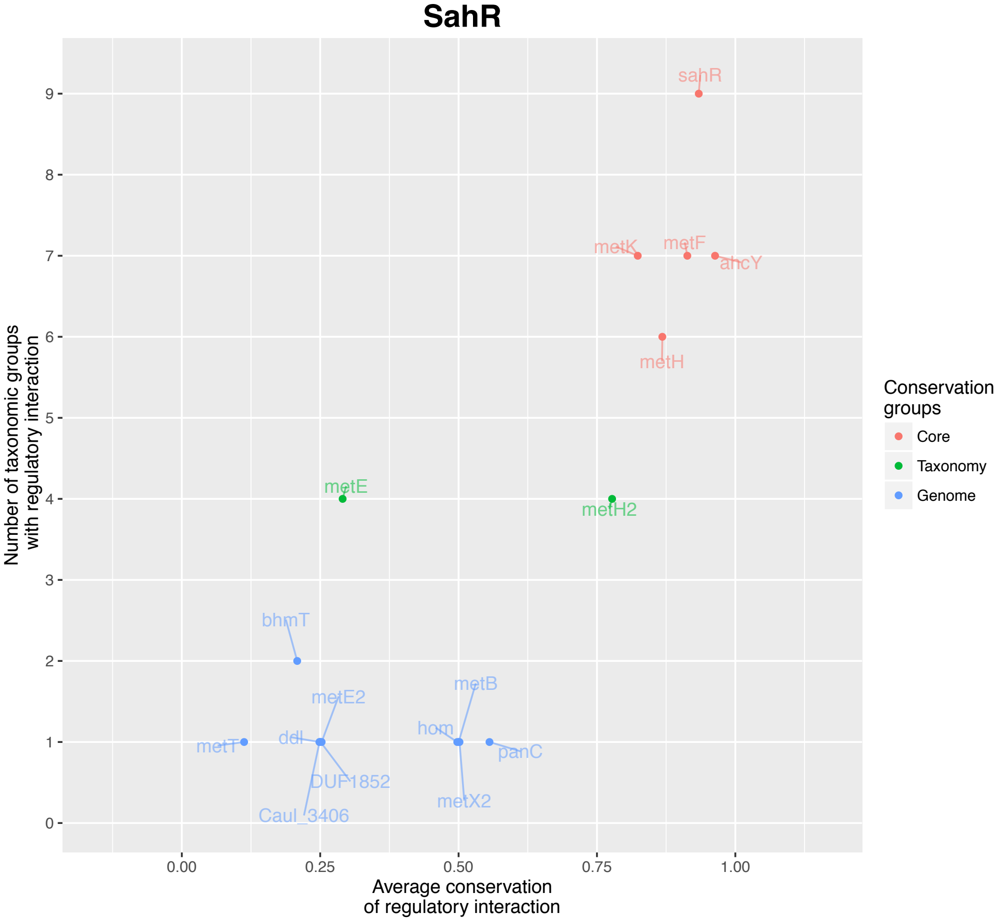
SahR
Number of taxonomic groups
with regulatory interaction
Average conservation
of regulatory interaction
Conservation
groups
Core
Taxonomy
Genome
sahR
metK
metF
ahcY
metH
metE
metH2
bhmT
metE2
metB
hom
metT
ddl
panC
DUF1852
metX2
Caul_3406
0.00
0.25
0.50
0.75
1.00

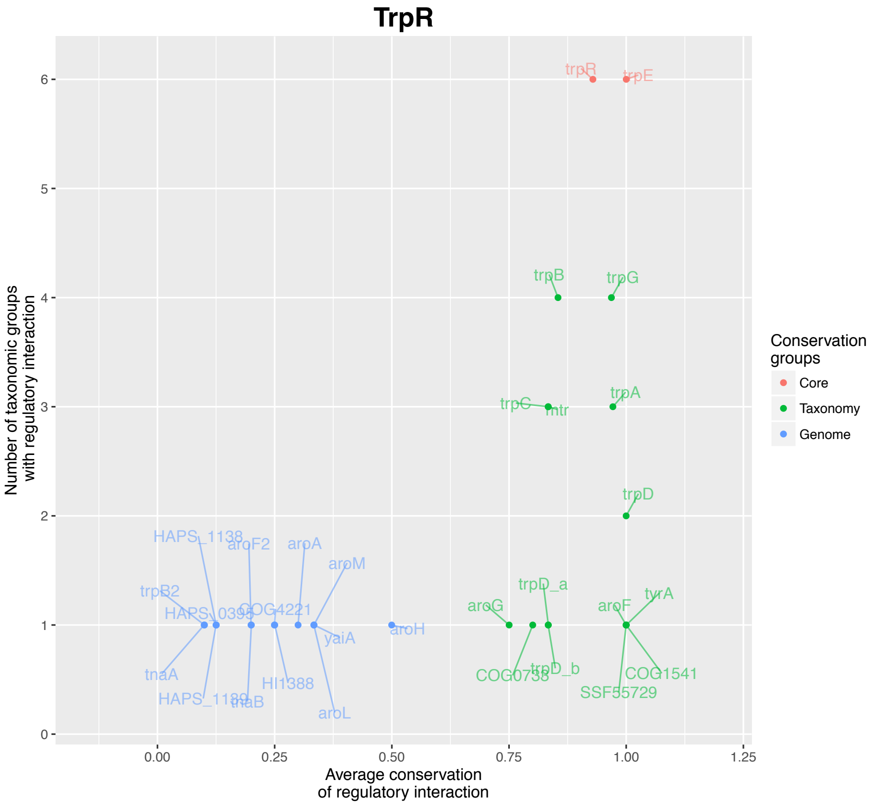
TrpR
Number of taxonomic groups with regulatory interaction
Average conservation of regulatory interaction
0.00
0.25
0.50
0.75
1.00
1.25
0
1
2
3
4
5
6
Conservation groups
Core
Taxonomy
Genome
trpR
trpE
trpB
trpG
trpC
mtr
trpA
trpD
trpD_a
aroG
aroF
tyrA
COG0738
trpD_b
COG1541
SSF55729
HAPS_1138
aroF2
aroA
aroM
trpB2
HAPS_0395
COG4221
yaiA
aroH
tnaA
HAPS_1189
tnaB
HI1388
aroL

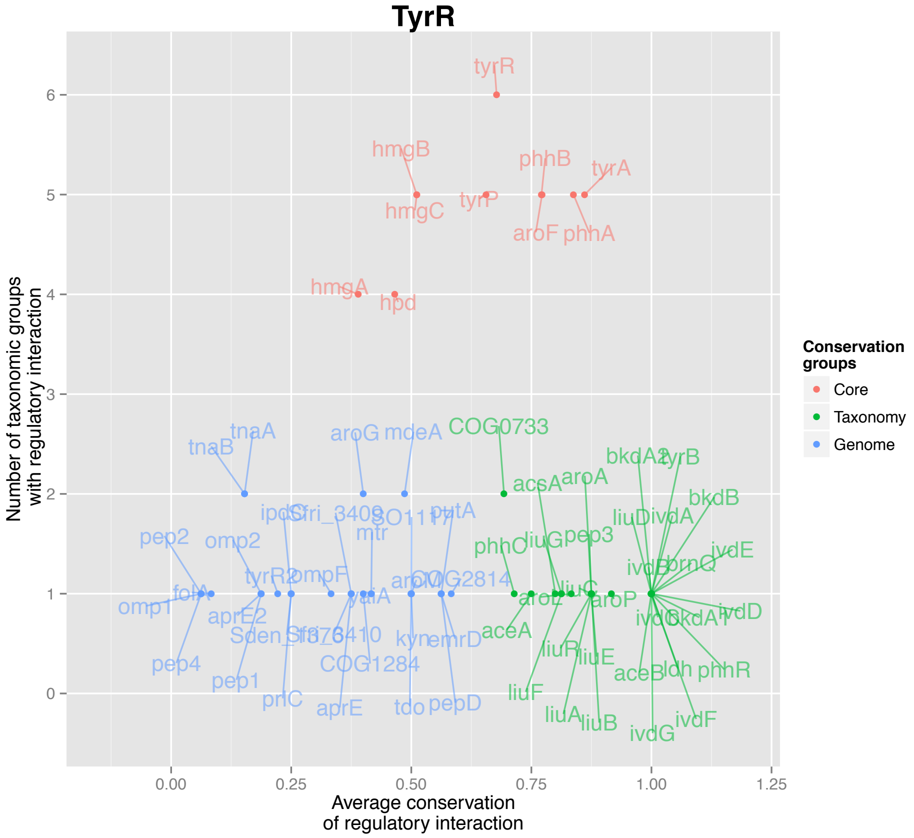
TyrR
Number of taxonomic groups with regulatory interaction
Average conservation of regulatory interaction
0.00
0.25
0.50
0.75
1.00
1.25
0
1
2
3
4
5
6
Conservation groups
Core
Taxonomy
Genome
tyrR
hmgB
phhB
tyrA
tyrP
hmgC
aroF
phhA
hmgA
hpd
tnaA
tnaB
aroG
mdeA
COG0733
bkdA2
tyrB
acsA
aroA
bkdB
liuD
ivdA
putA
ivdE
pep2
omp2
mtr
phhC
liuG
pep3
ompF
ivdB
brnQ
tyrR2
COG2814
liuC
aroP
omp1
aprE2
ivdC
bkdA1
ivdD
aceA
COG1284
liuH
liuE
pep4
aceB
ldh
phhR
pep1
liuF
prlC
aprE
tdo
pepD
liuA
liuB
ivdF
ivdG

Table S1. Studied genomes and taxonomic groups of Proteobacteria.

Tax ID	Phylum / Class / Taxonomic collection [1] / Genome	Number of genomes
**Phylum**	**Proteobacteria**	**196**
**Class**	**Gammaproteobacteria**	**90**
	**Enterobacteriales**	12
511145	Escherichia coli str. K-12 substr. MG1655	
99287	Salmonella typhimurium LT2	
290338	Citrobacter koseri ATCC BAA-895	
272620	Klebsiella pneumoniae subsp. pneumoniae MGH 78578	
399742	Enterobacter sp. 638	
716540	Erwinia amylovora ATCC 49946	
187410	Erwinia amylovora ATCC 49946	
399741	Serratia proteamaculans 568	
218491	Erwinia carotovora subsp. atroseptica SCRI1043	
498217	Edwardsiella tarda EIB202	
529507	Proteus mirabilis HI4320	
243265	Photorhabdus luminescens subsp. laumondii TTO1	
	**Pasteurellales**	9
71421	Haemophilus influenzae Rd KW20	
634176	Aggregatibacter aphrophilus NJ8700	
272843	Pasteurella multocida subsp. multocida str. Pm70	
221988	Mannheimia succiniciproducens MBEL55E	
339671	Actinobacillus succinogenes 130Z	
228400	Haemophilus somnus 2336	
537457	Actinobacillus pleuropneumoniae serovar 7 str. AP76	
233412	Haemophilus ducreyi 35000HP	
557723	Haemophilus parasuis SH0165	
	**Vibrionales**	10
243277	Vibrio cholerae O1 biovar eltor str. N16961	
216895	Vibrio vulnificus CMCP6	
338187	Vibrio harveyi ATCC BAA-1116	
223926	Vibrio parahaemolyticus RIMD 2210633	
391591	Vibrio shilonii AK1	
575788	Vibrio splendidus LGP32	
312309	Vibrio fischeri ES114	
316275	Vibrio salmonicida LFI1238	
314292	Vibrio angustum S14	
298386	Photobacterium profundum SS9	
	**Psychromonadaceae/Aeromonadales**	6
357804	Psychromonas ingrahamii 37	
314282	Psychromonas sp. CNPT3	
58051	Moritella sp. PE36	
380703	Aeromonas hydrophila subsp. hydrophila ATCC 7966	
382245	Aeromonas salmonicida subsp. salmonicida A449	
595494	Tolumonas auensis DSM 9187	
	**Shewanellaceae**	16
211586	Shewanella oneidensis MR-1	
319224	Shewanella putrefaciens CN-32	
351745	Shewanella sp W3-18-1	

94122	Shewanella sp ANA-3	
60480	Shewanella sp MR-4	
60481	Shewanella sp MR-7	
325240	Shewanella baltica OS155	
318161	Shewanella denitrificans OS217	
318167	Shewanella frigidimarina NCIMB 400	
326297	Shewanella amazonensis SB2B	
323850	Shewanella loihica PV-4	
398579	Shewanella pealeana ATCC 700345	
458817	Shewanella halifaxensis HAW-EB4	
225849	Shewanella piezotolerans WP3	
425104	Shewanella sediminis HAW-EB3	
392500	Shewanella woodyi ATCC 51908	
	**Alteromonadales**	9
342610	Pseudoalteromonas atlantica T6c	
314275	Alteromonas macleodii 'Deep ecotype'	
455436	Glaciecola sp. HTCC2999	
167879	Colwellia psychrerythraea 34H	
156578	Alteromonadales bacterium TW-7	
326442	Pseudoalteromonas haloplanktis TAC125	
87626	Pseudoalteromonas tunicata D2	
314276	Idiomarina baltica OS145	
283942	Idiomarina loihiensis L2TR	
	**Oceanospirillales/Alteromonadales**	12
349521	Hahella chejuensis KCTC 2396	
351348	Marinobacter aqueolei	
270374	Marinobacter sp. ELB17	
207949	Oceanobacter sp. RED65	
207954	Oceanospirillum sp. MED92	
400668	Marinomonas sp. MWYL1	
203122	Saccharophagus degradans 2-40	
377629	Teredinibacter turnerae T7901	
498211	Cellvibrio japonicus Ueda107	
290398	Chromohalobacter salexigens DSM 3043	
314283	Reinekea sp. MED297	
393595	Alcanivorax borkumensis SK2	
	**Pseudomonadaceae**	8
208964	Pseudomonas aeruginosa PAO1	
384676	Pseudomonas entomophila L48	
160488	Pseudomonas putida KT2440	
223283	Pseudomonas syringae pv. tomato str. DC3000	
220664	Pseudomonas fluorescens Pf-5	
399739	Pseudomonas mendocina ymp	
379731	Pseudomonas stutzeri A1501	
322710	Azotobacter vinelandii AvOP	
	**Moraxellaceae**	4
62977	Acinetobacter sp. ADP1	
480119	Acinetobacter baumannii AB0057	
259536	Psychrobacter arcticum 273-4	
349106	Psychrobacter sp. PRwf-1	
	**Xanthomonadales**	4

160492	Xylella fastidiosa 9a5c	
190486	Xanthomonas axonopodis pv. citri str. 306	
190485	Xanthomonas campestris pv. campestris str. ATCC 33913	
522373	Stenotrophomonas maltophilia K279a	
**Class**	**Betaproteobacteria**	37
	**Ralstonia**	6
381666	Ralstonia eutropha H16	
164546	Cupriavidus taiwanensis	
266264	Ralstonia metallidurans CH34	
264198	Ralstonia eutropha JMP134	
267608	Ralstonia solanacearum GMI1000	
402626	Ralstonia pickettii 12J	
	**Burkholderia**	8
272560	Burkholderia pseudomallei K96243	
243160	Burkholderia mallei ATCC 23344	
269483	Burkholderia sp. 383	
339670	Burkholderia cepacia AMMD (Burkholderia ambifaria AMMD)	
269482	Burkholderia vietnamiensis G4	
626418	Burkholderia glumae BGR1	
266265	Burkholderia xenovorans LB400	
391038	Burkholderia phymatum STM815	
	**Comamonadaceae**	11
397945	Acidovorax avenae subsp. citrulli AAC00-1	
232721	Acidovorax sp. JS42	
399795	Comamonas testosteroni KF-1	
398578	Delftia acidovorans SPH-1	
365044	Polaromonas naphthalenivorans CJ2	
296591	Polaromonas sp. JS666	
338969	Rhodoferax ferrireducens DSM 15236	
543728	Variovorax paradoxus S110	
391735	Verminephrobacter eiseniae EF01-2	
420662	Methylibium petroleiphilum PM1	
395495	Leptothrix cholodnii SP-6	
	**Various betaproteobacteria**	12
76114	Azoarcus sp. EbN1	
85643	Thauera sp. MZ1T	
159087	Dechloromonas aromatica RCB	
228410	Nitrosomonas europaea ATCC 19718	
323848	Nitrosospira multiformis ATCC 25196	
292415	Thiobacillus denitrificans	
243365	Chromobacterium violaceum ATCC 12472	
122586	Neisseria meningitidis MC58	
557598	Laribacter hongkongensis HLHK9	
265072	Methylobacillus flagellatus KT	
583345	Methylotenera mobilis JLW8	
383631	Methylophilales bacterium HTCC2181	
**Class**	**Alphaproteobacteria**	**50**
	**Rhizobiales**	15
266834	Sinorhizobium meliloti 1021	
394	Rhizobium sp. NGR234	
216596	Rhizobium leguminosarum bv. viciae 3841	

347834	Rhizobium etli CFN 42	
176299	Agrobacterium tumefaciens str. C58 (Cereon)	
266779	Mesorhizobium sp. BNC1	
266835	Mesorhizobium loti MAFF303099	
224914	Brucella melitensis 16M	
283165	Bartonella quintana str. Toulouse	
258594	Rhodopseudomonas palustris CGA009	
224911	Bradyrhizobium japonicum USDA 110	
288000	Bradyrhizobium sp. BTAi1	
323098	Nitrobacter winogradskyi Nb-255	
438753	Azorhizobium caulinodans ORS 571	
78245	Xanthobacter autotrophicus Py2	
	**Rhodobacterales**	15
272943	Rhodobacter sphaeroides 2.4.1	
318586	Paracoccus denitrificans PD1222	
290400	Jannaschia sp. CCS1	
314271	Rhodobacterales bacterium HTCC2654	
314256	Oceanicola granulosus HTCC2516	
314232	Loktanella vestfoldensis SKA53	
252305	Oceanicola batsensis HTCC2597	
89187	Roseovarius nubinhibens ISM	
314264	Roseovarius sp. 217	
52598	Sulfitobacter sp. EE-36	
292414	Silicibacter TM1040	
246200	Silicibacter pomeroyi DSS-3	
314262	Roseobacter sp. MED193	
228405	Hyphomonas neptunium ATCC 15444	
314254	Oceanicaulis alexandrii HTCC2633	
	**Rhodospirillales**	9
269796	Rhodospirillum rubrum ATCC 11170	
342108	Magnetospirillum magneticum AMB-1	
272627	Magnetospirillum magnetotacticum MS-1	
137722	Azospirillum sp. B510	
414684	Rhodospirillum centenum SW	
272568	Gluconacetobacter diazotrophicus PAl 5	
634452	Acetobacter pasteurianus IFO 3283-01	
290633	Gluconobacter oxydans 621H	
391165	Granulibacter bethesdensis CGDNIH1	
	**Sphingomonadales**	7
314225	Erythrobacter litoralis HTCC2594	
237727	Erythrobacter sp. NAP1	
279238	Novosphingobium aromaticivorans DSM 12444	
317655	Sphingopyxis alaskensis RB2256	
452662	Sphingobium japonicum UT26S	
392499	Sphingomonas wittichii RW1	
264203	Zymomonas mobilis subsp. mobilis ZM4	
	**Caulobacterales**	4
190650	Caulobacter crescentus CB15	
509190	Caulobacter segnis ATCC 21756	
366602	Caulobacter sp. K31	
450851	Phenylobacterium zucineum HLK1	

**Class**	**Proteobacteria/Delta**	**19**
	**Desulfovibrionales**	10
882	Desulfovibrio vulgaris Hildenborough	
883	Desulfovibrio vulgaris str. Miyazaki F	
207559	Desulfovibrio desulfuricans G20	
525146	Desulfovibrio desulfuricans subsp. desulfuricans str. ATCC 27774	
411464	Desulfovibrio piger ATCC 29098	
526222	Desulfovibrio salexigens DSM 2638	
573370	Desulfovibrio magneticus RS-1	
363253	Lawsonia intracellularis PHE/MN1-00	
525897	Desulfomicrobium baculatum DSM 4028	
485915	Desulfohalobium retbaense DSM 5692	
	**Desulfuromonadales**	9
269799	Geobacter metallireducens GS-15	
243231	Geobacter sulfurreducens PCA	
351605	Geobacter uraniumreducens Rf4	
316067	Geobacter sp. FRC-32	
443144	Geobacter sp. M21	
398767	Geobacter lovleyi SZ	
338966	Pelobacter propionicus DSM 2379	
338963	Pelobacter carbinolicus str. DSM 2380	
281689	Desulfuromonas acetoxidans DSM 684	

[1] Taxonomic collections are according to the standartized genomic collections in the RegPrecise database.

Table S2. Examples of experimentally studied TFs analyzed in this work.

TF	Genome	Reference
ArgR	*Escherichia coli*	(Tian et al., 1992;Caldara et al., 2007;Paul et al., 2007;Cho et al., 2015)
	Salmonella typhimurium	(Lu and Abdelal, 1999)
BioR	*Brucella melitensis*	(Feng et al., 2013)
	Paracoccus denitrificans	(Feng et al., 2015)
BirA	*Escherichia coli*	(Bower et al., 1995;Xu et al., 1995)
FabR	*Escherichia coli*	(Zhang et al., 2002;Fujita et al., 2007)
FadR	*Escherichia coli*	(DiRusso et al., 1992;Fujita et al., 2007)
GlcC	*Escherichia coli*	(Pellicer et al., 1999)
HexR	*Shewanella oneidensis*	(Leyn et al., 2011)
	Pseudomonas putida	(del Castillo et al., 2008;Daddaoua et al., 2009)
HmgR	*Pseudomonas putida*	(Arias-Barrau et al., 2004)
HutC	*Salmonella typhimurium*	(Hagen et al., 1975)
HypR	*Sinorhizobium meliloti*	(White et al., 2012)
LldR	*Pseudomonas aeruginosa*	(Gao et al., 2012)
	Escherichia coli	(Aguilera et al., 2008)
MetJ	*Escherichia coli*	(Merlin et al., 2002)
MetR	*Escherichia coli*	(Cai et al., 1989)
	Vibrio cholerae	(Bogard et al., 2012)
NadR	*Salmonella typhimurium*	(Foster et al., 1990)
NagC	*Escherichia coli*	(Plumbridge, 1995; 2001)
NagQ	*Xanthomonas campestris*	(Boulanger et al., 2010)
NagR	*Xanthomonas campestris*	(Boulanger et al., 2010)
	Shewanella oneidensis	(Rodionov et al., 2011)
NrdR	*Escherichia coli*	(Torrents et al., 2007)
	Salmonella typhimurium	(Panosa et al., 2010)
	Pseudomonas aeruginosa	(Crespo et al., 2015)
NrtR	*Shewanella oneidensis*	(Rodionov et al., 2008)
NtrC	*Escherichia coli*	(Muse and Bender, 1998;Zimmer et al., 2000)
PdhR	*Escherichia coli*	(Quail and Guest, 1995;Ogasawara et al., 2007)
PsrA	*Pseudomonas putida*	(Kojic et al., 2002;Fonseca et al., 2014)
	Pseudomonas aeruginosa	(Kang et al., 2009)
RutR	*Escherichia coli*	(Shimada et al., 2007;Nguyen Ple et al., 2010;Nguyen Le Minh et al., 2015)
SahR	*Desulfovibrio alaskensis*	(Novichkov et al., 2014)
TrpR	*Escherichia coli*	(Czernik et al., 1994;Jeeves et al., 1999)
TyrR	*Escherichia coli*	(Camakaris and Pittard, 1982;Yang et al., 2002), (Yang et al., 2004;Pittard et al., 2005)
TyrR	*Enterobacter cloacae*	(Coulson and Patten, 2015)
TyrR	*Citrobacter freundii*	(Smith and Somerville, 1997)
PhhR	*Pseudomonas aeruginosa*	(Palmer et al., 2010)
PhhR	*Pseudomonas putida*	(Herrera et al., 2009;Herrera et al., 2010)

References:

Aguilera, L., Campos, E., Gimenez, R., Badia, J., Aguilar, J., and Baldoma, L. (2008). Dual role of LldR in regulation of the lldPRD operon, involved in L-lactate metabolism in Escherichia coli. *J Bacteriol* 190**,** 2997-3005.

Arias-Barrau, E., Olivera, E.R., Luengo, J.M., Fernandez, C., Galan, B., Garcia, J.L., Diaz, E., and Minambres, B. (2004). The homogentisate pathway: a central catabolic pathway involved in the degradation of L-phenylalanine, L-tyrosine, and 3-hydroxyphenylacetate in Pseudomonas putida. *J Bacteriol* 186**,** 5062-5077.

Bogard, R.W., Davies, B.W., and Mekalanos, J.J. (2012). MetR-regulated Vibrio cholerae metabolism is required for virulence. *MBio* 3.

Boulanger, A., Dejean, G., Lautier, M., Glories, M., Zischek, C., Arlat, M., and Lauber, E. (2010). Identification and regulation of the N-acetylglucosamine utilization pathway of the plant pathogenic bacterium Xanthomonas campestris pv. campestris. *J Bacteriol* 192**,** 1487-1497.

Bower, S., Perkins, J., Yocum, R.R., Serror, P., Sorokin, A., Rahaim, P., Howitt, C.L., Prasad, N., Ehrlich, S.D., and Pero, J. (1995). Cloning and characterization of the Bacillus subtilis birA gene encoding a repressor of the biotin operon. *J Bacteriol* 177**,** 2572-2575.

Cai, X.Y., Maxon, M.E., Redfield, B., Glass, R., Brot, N., and Weissbach, H. (1989). Methionine synthesis in Escherichia coli: effect of the MetR protein on metE and metH expression. *Proc Natl Acad Sci U S A* 86**,** 4407-4411.

Caldara, M., Minh, P.N., Bostoen, S., Massant, J., and Charlier, D. (2007). ArgR-dependent repression of arginine and histidine transport genes in Escherichia coli K-12. *J Mol Biol* 373**,** 251-267.

Camakaris, H., and Pittard, J. (1982). Autoregulation of the tyrR gene. *J Bacteriol* 150**,** 70-75.

Cho, S., Cho, Y.B., Kang, T.J., Kim, S.C., Palsson, B., and Cho, B.K. (2015). The architecture of ArgR-DNA complexes at the genome-scale in Escherichia coli. *Nucleic Acids Res* 43**,** 3079-3088.

Coulson, T.J., and Patten, C.L. (2015). The TyrR transcription factor regulates the divergent akr-ipdC operons of Enterobacter cloacae UW5. *PLoS One* 10**,** e0121241.

Crespo, A., Pedraz, L., and Torrents, E. (2015). Function of the Pseudomonas aeruginosa NrdR Transcription Factor: Global Transcriptomic Analysis and Its Role on Ribonucleotide Reductase Gene Expression. *PLoS One* 10**,** e0123571.

Czernik, P.J., Shin, D.S., and Hurlburt, B.K. (1994). Functional selection and characterization of DNA binding sites for trp repressor of Escherichia coli. *J Biol Chem* 269**,** 27869-27875.

Daddaoua, A., Krell, T., and Ramos, J.L. (2009). Regulation of glucose metabolism in Pseudomonas: the phosphorylative branch and entner-doudoroff enzymes are regulated by a repressor containing a sugar isomerase domain. *J Biol Chem* 284**,** 21360-21368.

Del Castillo, T., Duque, E., and Ramos, J.L. (2008). A set of activators and repressors control peripheral glucose pathways in Pseudomonas putida to yield a common central intermediate. *J Bacteriol* 190**,** 2331-2339.

Dirusso, C.C., Heimert, T.L., and Metzger, A.K. (1992). Characterization of FadR, a global transcriptional regulator of fatty acid metabolism in Escherichia coli. Interaction with the fadB promoter is prevented by long chain fatty acyl coenzyme A. *J Biol Chem* 267**,** 8685-8691.

Feng, Y., Kumar, R., Ravcheev, D.A., and Zhang, H. (2015). Paracoccus denitrificans possesses two BioR homologs having a role in regulation of biotin metabolism. *Microbiologyopen* 4**,** 644-659.

Feng, Y., Xu, J., Zhang, H., Chen, Z., and Srinivas, S. (2013). Brucella BioR regulator defines a complex regulatory mechanism for bacterial biotin metabolism. *J Bacteriol* 195**,** 3451-3467.

Fonseca, P., De La Pena, F., and Prieto, M.A. (2014). A role for the regulator PsrA in the polyhydroxyalkanoate metabolism of Pseudomonas putida KT2440. *Int J Biol Macromol* 71**,** 14-20.

Foster, J.W., Park, Y.K., Penfound, T., Fenger, T., and Spector, M.P. (1990). Regulation of NAD metabolism in Salmonella typhimurium: molecular sequence analysis of the bifunctional nadR regulator and the nadA-pnuC operon. *J Bacteriol* 172**,** 4187-4196.

Fujita, Y., Matsuoka, H., and Hirooka, K. (2007). Regulation of fatty acid metabolism in bacteria. *Mol Microbiol* 66**,** 829-839.

Gao, C., Hu, C., Zheng, Z., Ma, C., Jiang, T., Dou, P., Zhang, W., Che, B., Wang, Y., Lv, M., and Xu, P. (2012). Lactate utilization is regulated by the FadR-type regulator LldR in Pseudomonas aeruginosa. *J Bacteriol* 194**,** 2687-2692.

Hagen, D.C., Gerson, S.L., and Magasanik, B. (1975). Isolation of super-repressor mutants in the histidine utilization system of Salmonella typhimurium. *J Bacteriol* 121**,** 583-593.

Herrera, M.C., Duque, E., Rodriguez-Herva, J.J., Fernandez-Escamilla, A.M., and Ramos, J.L. (2010). Identification and characterization of the PhhR regulon in Pseudomonas putida. *Environ Microbiol* 12**,** 1427-1438.

Herrera, M.C., Krell, T., Zhang, X., and Ramos, J.L. (2009). PhhR binds to target sequences at different distances with respect to RNA polymerase in order to activate transcription. *J Mol Biol* 394**,** 576-586.

Jeeves, M., Evans, P.D., Parslow, R.A., Jaseja, M., and Hyde, E.I. (1999). Studies of the Escherichia coli Trp repressor binding to its five operators and to variant operator sequences. *Eur J Biochem* 265**,** 919-928.

Kang, Y., Lunin, V.V., Skarina, T., Savchenko, A., Schurr, M.J., and Hoang, T.T. (2009). The long-chain fatty acid sensor, PsrA, modulates the expression of rpoS and the type III secretion exsCEBA operon in Pseudomonas aeruginosa. *Mol Microbiol* 73**,** 120-136.

Kojic, M., Aguilar, C., and Venturi, V. (2002). TetR family member psrA directly binds the Pseudomonas rpoS and psrA promoters. *J Bacteriol* 184**,** 2324-2330.

Leyn, S.A., Li, X., Zheng, Q., Novichkov, P.S., Reed, S., Romine, M.F., Fredrickson, J.K., Yang, C., Osterman, A.L., and Rodionov, D.A. (2011). Control of proteobacterial central carbon metabolism by the HexR transcriptional regulator: a case study in Shewanella oneidensis. *J Biol Chem* 286**,** 35782-35794.

Lu, C.D., and Abdelal, A.T. (1999). Role of ArgR in activation of the ast operon, encoding enzymes of the arginine succinyltransferase pathway in Salmonella typhimurium. *J Bacteriol* 181**,** 1934-1938.

Merlin, C., Gardiner, G., Durand, S., and Masters, M. (2002). The Escherichia coli metD locus encodes an ABC transporter which includes Abc (MetN), YaeE (MetI), and YaeC (MetQ). *J Bacteriol* 184**,** 5513-5517.

Muse, W.B., and Bender, R.A. (1998). The nac (nitrogen assimilation control) gene from Escherichia coli. *J Bacteriol* 180**,** 1166-1173.

Nguyen Le Minh, P., De Cima, S., Bervoets, I., Maes, D., Rubio, V., and Charlier, D. (2015). Ligand binding specificity of RutR, a member of the TetR family of transcription regulators in Escherichia coli. *FEBS Open Bio* 5**,** 76-84.

Nguyen Ple, M., Bervoets, I., Maes, D., and Charlier, D. (2010). The protein-DNA contacts in RutR*carAB operator complexes. *Nucleic Acids Res* 38**,** 6286-6300.

Novichkov, P.S., Li, X., Kuehl, J.V., Deutschbauer, A.M., Arkin, A.P., Price, M.N., and Rodionov, D.A. (2014). Control of methionine metabolism by the SahR transcriptional regulator in Proteobacteria. *Environ Microbiol* 16**,** 1-8.

Ogasawara, H., Ishida, Y., Yamada, K., Yamamoto, K., and Ishihama, A. (2007). PdhR (pyruvate dehydrogenase complex regulator) controls the respiratory electron transport system in Escherichia coli. *J Bacteriol* 189**,** 5534-5541.

Palmer, G.C., Palmer, K.L., Jorth, P.A., and Whiteley, M. (2010). Characterization of the Pseudomonas aeruginosa transcriptional response to phenylalanine and tyrosine. *J Bacteriol* 192**,** 2722-2728.

Panosa, A., Roca, I., and Gibert, I. (2010). Ribonucleotide reductases of Salmonella typhimurium: transcriptional regulation and differential role in pathogenesis. *PLoS One* 5**,** e11328.

Paul, L., Mishra, P.K., Blumenthal, R.M., and Matthews, R.G. (2007). Integration of regulatory signals through involvement of multiple global regulators: control of the Escherichia coli gltBDF operon by Lrp, IHF, Crp, and ArgR. *BMC Microbiol* 7**,** 2.

Pellicer, M.T., Fernandez, C., Badia, J., Aguilar, J., Lin, E.C., and Baldom, L. (1999). Cross-induction of glc and ace operons of Escherichia coli attributable to pathway intersection. Characterization of the glc promoter. *J Biol Chem* 274**,** 1745-1752.

Pittard, J., Camakaris, H., and Yang, J. (2005). The TyrR regulon. *Mol Microbiol* 55**,** 16-26.
Plumbridge, J. (1995). Co-ordinated regulation of amino sugar biosynthesis and degradation: the NagC repressor acts as both an activator and a repressor for the transcription of the glmUS operon and requires two separated NagC binding sites. *EMBO J* 14**,** 3958-3965.
Plumbridge, J. (2001). DNA binding sites for the Mlc and NagC proteins: regulation of nagE, encoding the N-acetylglucosamine-specific transporter in Escherichia coli. *Nucleic Acids Res* 29**,** 506-514.
Quail, M.A., and Guest, J.R. (1995). Purification, characterization and mode of action of PdhR, the transcriptional repressor of the pdhR-aceEF-lpd operon of Escherichia coli. *Mol Microbiol* 15**,** 519-529.
Rodionov, D.A., De Ingeniis, J., Mancini, C., Cimadamore, F., Zhang, H., Osterman, A.L., and Raffaelli, N. (2008). Transcriptional regulation of NAD metabolism in bacteria: NrtR family of Nudix-related regulators. *Nucleic Acids Res* 36**,** 2047-2059.
Rodionov, D.A., Novichkov, P.S., Stavrovskaya, E.D., Rodionova, I.A., Li, X., Kazanov, M.D., Ravcheev, D.A., Gerasimova, A.V., Kazakov, A.E., Kovaleva, G.Y., Permina, E.A., Laikova, O.N., Overbeek, R., Romine, M.F., Fredrickson, J.K., Arkin, A.P., Dubchak, I., Osterman, A.L., and Gelfand, M.S. (2011). Comparative genomic reconstruction of transcriptional networks controlling central metabolism in the Shewanella genus. *BMC Genomics* 12 Suppl 1**,** S3.
Shimada, T., Hirao, K., Kori, A., Yamamoto, K., and Ishihama, A. (2007). RutR is the uracil/thymine-sensing master regulator of a set of genes for synthesis and degradation of pyrimidines. *Mol Microbiol* 66**,** 744-757.
Smith, H.Q., and Somerville, R.L. (1997). The tpl promoter of Citrobacter freundii is activated by the TyrR protein. *J Bacteriol* 179**,** 5914-5921.
Tian, G., Lim, D., Carey, J., and Maas, W.K. (1992). Binding of the arginine repressor of Escherichia coli K12 to its operator sites. *J Mol Biol* 226**,** 387-397.
Torrents, E., Grinberg, I., Gorovitz-Harris, B., Lundstrom, H., Borovok, I., Aharonowitz, Y., Sjoberg, B.M., and Cohen, G. (2007). NrdR controls differential expression of the Escherichia coli ribonucleotide reductase genes. *J Bacteriol* 189**,** 5012-5021.
White, C.E., Gavina, J.M., Morton, R., Britz-Mckibbin, P., and Finan, T.M. (2012). Control of hydroxyproline catabolism in Sinorhizobium meliloti. *Mol Microbiol* 85**,** 1133-1147.
Xu, Y., Nenortas, E., and Beckett, D. (1995). Evidence for distinct ligand-bound conformational states of the multifunctional Escherichia coli repressor of biotin biosynthesis. *Biochemistry* 34**,** 16624-16631.
Yang, J., Camakaris, H., and Pittard, J. (2002). Molecular analysis of tyrosine-and phenylalanine-mediated repression of the tyrB promoter by the TyrR protein of Escherichia coli. *Mol Microbiol* 45**,** 1407-1419.
Yang, J., Hwang, J.S., Camakaris, H., Irawaty, W., Ishihama, A., and Pittard, J. (2004). Mode of action of the TyrR protein: repression and activation of the tyrP promoter of Escherichia coli. *Mol Microbiol* 52**,** 243-256.
Zhang, Y.M., Marrakchi, H., and Rock, C.O. (2002). The FabR (YijC) transcription factor regulates unsaturated fatty acid biosynthesis in Escherichia coli. *J Biol Chem* 277**,** 15558-15565.
Zimmer, D.P., Soupene, E., Lee, H.L., Wendisch, V.F., Khodursky, A.B., Peter, B.J., Bender, R.A., and Kustu, S. (2000). Nitrogen regulatory protein C-controlled genes of Escherichia coli: scavenging as a defense against nitrogen limitation. *Proc Natl Acad Sci U S A* 97**,** 14674-14679.

Table S3. Reconstructed regulatory interactions and functional annotations for the analyzed TF regulons of Proteobacteria.

Regulon	Target gene	RI [1]	Taxa [2]	Functional role	Metabolic pathway / Biological process
**ArgR**	argH	57	6	Argininosuccinate lyase (EC 4.3.2.1)	Arginine biosynthesis
**ArgR**	argB	54	6	Acetylglutamate kinase (EC 2.7.2.8)	Arginine biosynthesis
**ArgR**	argC	53	6	N-acetyl-gamma-glutamyl-phosphate reductase (EC 1.2.1.38)	Arginine biosynthesis
**ArgR**	argG	52	5	Argininosuccinate synthase (EC 6.3.4.5)	Arginine biosynthesis
**ArgR**	argF	52	5	Ornithine carbamoyltransferase (EC 2.1.3.3)	Arginine biosynthesis
**ArgR**	argA	51	6	N-acetylglutamate synthase (EC 2.3.1.1)	Arginine biosynthesis
**ArgR**	argR	48	5	Arginine biosynthesis transcription regulator ArgR, ArgR family	Arginine biosynthesis
**ArgR**	argE	42	6	Acetylornithine deacetylase (EC 3.5.1.16)	Arginine biosynthesis
**ArgR**	artI	41	5	Arginine ABC transporter, substrate-binding protein	Arginine transport
**ArgR**	artQ	39	5	Arginine ABC transporter, permease protein 1	Arginine transport
**ArgR**	artM	39	5	Arginine ABC transporter, permease protein 2	Arginine transport
**ArgR**	astD	37	4	Succinylglutamic semialdehyde dehydrogenase (EC 1.2.1.71)	Arginine degradation
**ArgR**	astA	37	4	Arginine N-succinyltransferase (EC 2.3.1.109)	Arginine degradation
**ArgR**	astC	31	3	Acetylornithine aminotransferase (EC 2.6.1.11) / N-succinyl-L,L-diaminopimelate aminotransferase (EC 2.6.1.17) / Succinylornithine transaminase (EC 2.6.1.81)	Arginine degradation
**ArgR**	astB	21	2	Succinylarginine dihydrolase (EC 3.5.3.23)	Arginine degradation
**ArgR**	artP	28	4	Arginine ABC transporter, ATP-binding protein	Arginine transport
**ArgR**	carA	25	4	Carbamoyl-phosphate synthase small chain (EC 6.3.5.5)	Arginine and pyrimidine biosynthesis
**ArgR**	carB	24	4	Carbamoyl-phosphate synthase large chain (EC 6.3.5.5)	Arginine and pyrimidine biosynthesis
**ArgR**	argD	22	3	Acetylornithine aminotransferase (EC 2.6.1.11)	Arginine biosynthesis
**ArgR**	gltB	34	3	Glutamate synthase [NADPH] large chain (EC 1.4.1.13)	Glutamate biosynthesis
**ArgR**	gltD	33	3	Glutamate synthase [NADPH] small chain (EC 1.4.1.13)	Glutamate biosynthesis
**ArgR**	argW	17	2	Predicted arginine uptake transporter, COG3314 family	Arginine transport
**ArgR**	SO0620	15	1	Conserved hypothetical protein	
**ArgR**	ilvM	14	1	Acetolactate synthase small subunit (EC 2.2.1.6)	Branched-chain amino acid biosynthesis
**ArgR**	SO3392	14	1	NADH-dependent flavin oxidoreductase, Oye family	
**ArgR**	ilvG	14	1	Acetolactate synthase large subunit (EC 2.2.1.6)	Branched-chain amino acid biosynthesis
**ArgR**	ilvD	14	1	Dihydroxy-acid dehydratase (EC 4.2.1.9)	Branched-chain amino acid biosynthesis
**ArgR**	ilvA	14	1	Threonine dehydratase biosynthetic (EC 4.3.1.19)	Branched-chain amino acid biosynthesis
**ArgR**	artJ	10	1	arginine ABC transporter, substrate-binding protein	Arginine transport
**ArgR**	yfcH	7	1	Conserved hypothetical protein	
**ArgR**	omp	14	1	TonB-dependent outer membrane transporter	Arginine transport ?
**ArgR**	oadA	12	1	Oxaloacetate decarboxylase, alpha chain (EC 4.1.1.3)	Pyruvate metabolism
**ArgR**	oadB	12	1	Oxaloacetate decarboxylase, beta chain (EC 4.1.1.3)	Pyruvate metabolism
**ArgR**	oadG	12	1	Oxaloacetate decarboxylase gamma chain (EC 4.1.1.3)	Pyruvate metabolism
**ArgR**	potF	12	1	Putrescine ABC transporter, substrate-binding protein (TC 3.A.1.11.2)	Putrescine transport
**ArgR**	potG	12	1	Putrescine ABC transporter, ATP-binding protein (TC 3.A.1.11.2)	Putrescine transport
**ArgR**	potH	12	1	Putrescine ABC transporter, permease protein 1 (TC 3.A.1.11.2)	Putrescine transport
**ArgR**	potI	12	1	Putrescine ABC transporter, permease protein 2 (TC 3.A.1.11.2)	Putrescine transport
**ArgR**	recN	12	1	DNA repair protein RecN	DNA repair
**ArgR**	aprE	11	1	Alkaline serine protease	
**ArgR**	SO0762	10	1	Isochorismate hydrolase (EC 3.3.2.1)	
**ArgR**	astE	7	2	succinylglutamate desuccinylase	Arginine degradation
**ArgR**	SO2753	9	1	Prolyl endopeptidase (EC 3.4.21.26)	
**ArgR**	arcA	5	1	Arginine deiminase (EC 3.5.3.6)	Arginine degradation
**ArgR**	arcB	1	1	Ornithine carbamoyltransferase (EC 2.1.3.3), catabolic	Arginine degradation
**ArgR**	arcC	1	1	Carbamate kinase (EC 2.7.2.2)	Arginine degradation

**ArgR**	arcD	1	1	Arginine/ornithine antiporter	Arginine degradation
**ArgR**	hisJ	6	1	histidine ABC transporter, substrate-binding protein	Histidine transport
**ArgR**	hisM	6	1	histidine ABC transporter, inner membrane permease	Histidine transport
**ArgR**	hisP	6	1	histidine ABC transporter, ATP-binding protein	Histidine transport
**ArgR**	hisQ	6	1	histidine ABC transporter, permease protein	Histidine transport
**ArgR**	potE	8	1	Putrescine/ornithine antiporter	Putrescine transport
**ArgR**	proV	3	1	Glycine betaine/L-proline ABC transporter, ATP-binding protein	Proline transport
**ArgR**	proW	3	1	Glycine betaine/L-proline ABC transporter, permease protein	Proline transport
**ArgR**	proX	3	1	Glycine betaine/L-proline ABC transporter, substrate-binding protein	Proline transport
**ArgR**	SO0312	8	1	Predicted outer membrane porin	
**ArgR**	ggt2	7	1	Gamma-glutamyltranspeptidase (EC 2.3.2.2)	Glutathione metabolism
**ArgR**	SO4732	7	1	Conserved hypothetical protein	
**ArgR**	mcp	6	1	Methyl-accepting chemotaxis protein	
**ArgR**	aprE2	5	1	Cold-active alkaline serine protease (EC 3.4.21.62)	
**ArgR**	ilvE	5	1	Branched-chain amino acid aminotransferase (EC 2.6.1.42)	Branched-chain amino acid biosynthesis
**ArgR**	ECA3537	3	1	amino acid-binding protein	
**ArgR**	ECA3538	3	1	polar amino acid ABC transporter, inner membrane subunit	
**ArgR**	ECA3539	3	1	amino acid ABC transporter, ATP-binding protein	
**ArgR**	hisA	4	1	Phosphoribosylformimino-5-aminoimidazole carboxamide ribotide isomerase (EC 5.3.1.16)	Histidine biosynthesis
**ArgR**	hisB	4	1	Histidinol-phosphatase (EC 3.1.3.15) / Imidazoleglycerol-phosphate dehydratase (EC 4.2.1.19)	Histidine biosynthesis
**ArgR**	hisC	4	1	Histidinol-phosphate aminotransferase (EC 2.6.1.9)	Histidine biosynthesis
**ArgR**	hisD	4	1	Histidinol dehydrogenase (EC 1.1.1.23)	Histidine biosynthesis
**ArgR**	hisF	4	1	Imidazole glycerol phosphate synthase cyclase subunit (EC 4.1.3.-)	Histidine biosynthesis
**ArgR**	hisG	4	1	ATP phosphoribosyltransferase (EC 2.4.2.17)	Histidine biosynthesis
**ArgR**	hisH	4	1	Imidazole glycerol phosphate synthase amidotransferase subunit (EC 2.4.2.-)	Histidine biosynthesis
**ArgR**	hisI	4	1	Phosphoribosyl-AMP cyclohydrolase (EC 3.5.4.19) / Phosphoribosyl-ATP pyrophosphatase (EC 3.6.1.31)	Histidine biosynthesis
**ArgR**	pbpG	4	1	D-alanyl-D-alanine endopeptidase	
**ArgR**	Swoo_0949	4	1	Peptidase U32	
**ArgR**	ybgH	3	1	amino acid/peptide transporter	
**ArgR**	SO1443	3	1	Conserved hypothetical protein	
**ArgR**	SO1915	3	1	Serine protease, subtilase family	
**ArgR**	SO2306	3	1	Cell division protein FtsK	
**ArgR**	marC	1	1	Membrane protein, MarC family	
**ArgR**	speF	1	1	Ornithine decarboxylase (EC 4.1.1.17)	Putrescine metabolism
**BioR**	bioB	12	2	Biotin synthase (EC 2.8.1.6)	Biotin biosynthesis
**BioR**	bioR	10	2	Biotin metabolism regulatory protein BioR, GntR family	Transcription regulation
**BioR**	bioY	8	2	Substrate-specific component BioY of biotin ECF transporter	Biotin transport
**BioR**	bioF	7	2	8-amino-7-oxononanoate synthase (EC 2.3.1.47)	Biotin biosynthesis
**BioR**	bioD	7	2	Dethiobiotin synthetase (EC 6.3.3.3)	Biotin biosynthesis
**BioR**	bioA	7	2	Adenosylmethionine-8-amino-7-oxononanoate aminotransferase (EC 2.6.1.62)	Biotin biosynthesis
**BioR**	bioZ	3	1	Biotin synthesis protein bioZ	Biotin biosynthesis
**BioR**	bioM	1	1	ATPase component BioM of energizing module of biotin ECF transporter	Biotin transport
**BioR**	bioG	1	1	Biotin synthesis protein bioG	Biotin biosynthesis
**BioR**	bioN	1	1	Transmembrane component BioN of energizing module of biotin ECF transporter	Biotin transport
**BioR**	bioC	1	1	Biotin synthesis protein bioC	Biotin biosynthesis
**BioR**	panD	1	1	aspartate alpha-decarboxylase	Pantothenate biosynthesis
**BirA**	bioB	93	11	Biotin synthase (EC 2.8.1.6)	Biotin biosynthesis
**BirA**	bioF	86	11	8-amino-7-oxononanoate synthase (EC 2.3.1.47)	Biotin biosynthesis

**BirA**	bioD	83	10	Dethiobiotin synthetase (EC 6.3.3.3)	Biotin biosynthesis
**BirA**	bioC	84	10	Biotin synthesis protein bioC	Biotin biosynthesis
**BirA**	bioA	65	8	Adenosylmethionine-8-amino-7-oxononanoate aminotransferase (EC 2.6.1.62)	Biotin biosynthesis
**BirA**	bioH	33	5	Biotin synthesis protein bioH	Biotin biosynthesis
**BirA**	COG1040	23	4	competence protein F	
**BirA**	fabF	3	1	3-oxoacyl-[acyl-carrier-protein] synthase, KASII (EC 2.3.1.41)	Fatty acid biosynthesis
**BirA**	birA	3	1	Biotin-protein ligase (EC 6.3.4.15) / Biotin operon repressor	Transcription regulation
**BirA**	fabG	3	1	3-oxoacyl-[acyl-carrier protein] reductase (EC 1.1.1.100)	Fatty acid biosynthesis
**BirA**	DVU2560	3	1	conserved domain protein	
**BirA**	acpP	3	1	acyl carrier protein, putative	
**BirA**	Dde_2651	2	1	hypothetical thioesterase domain protein	
**BirA**	XAC0384	2	1	putative short chain dehydrogenase	
**BirA**	XAC0386	2	1	hypothetical protein	
**BirA**	bioHC	3	1	Biotin synthesis protein BioH / Biotin synthesis protein BioC	Biotin biosynthesis
**BirA**	yigM	3	1	Predicted biotin transporter YigM	Biotin transport
**BirA**	CV3478	1	1	Phosphatidylethanolamine N-methyltransferase (EC 2.1.1.17)	Phosphatidylcholine biosynthesis
**BirA**	bioW	1	1	Pimeloyl-CoA synthase (EC 6.2.1.14)	Biotin biosynthesis
**FabR**	fabA	62	8	3-hydroxydecanoyl-[acyl-carrier-protein] dehydratase (EC 4.2.1.60)	Fatty acid biosynthesis
**FabR**	hyIII	30	6	COG1272: Predicted membrane protein hemolysin III homolog	Fatty acid metabolism
**FabR**	OLE1	38	5	Fatty acid desaturase (EC 1.14.19.1)	Unsaturated fatty acid biosynthesis
**FabR**	plsC	30	6	1-acyl-sn-glycerol-3-phosphate acyltransferase (EC 2.3.1.51)	Glycerolipid metabolism
**FabR**	desB	13	6	Acyl-CoA delta-9-desaturase, DesB	Unsaturated fatty acid biosynthesis
**FabR**	desC	13	6	Flavodoxin reductases (ferredoxin-NADPH reductases) family 1	Unsaturated fatty acid biosynthesis
**FabR**	lcfH	25	5	putative long-chain-fatty-acid--CoA ligase (EC 6.2.1.3)	Fatty acid biosynthesis
**FabR**	fabR	26	3	Unsaturated fatty acid biosythesis repressor FabR, TetR family	Transcription regulation
**FabR**	fadL	25	3	Long-chain fatty acid transport protein	Fatty acid biosynthesis
**FabR**	fabR2	5	2	Unsaturated fatty acid biosythesis repressor FabR, TetR family	Transcription regulation
**FabR**	fabB	20	4	3-oxoacyl-[acyl-carrier-protein] synthase, KASI (EC 2.3.1.41)	Fatty acid biosynthesis
**FabR**	rraB	17	2	Ribonuclease E inhibitor RraB	
**FabR**	lcfE	16	1	Long-chain-fatty-acid--CoA ligase (EC 6.2.1.3)	Fatty acid biosynthesis
**FabR**	hyIII	17	2	COG1272: Predicted membrane protein hemolysin III homolog	
**FabR**	Maqu_3149	2	1	AraC family transcriptional regulator	Transcription regulation
**FabR**	pfaA	7	2	omega-3 polyunsaturated fatty acid synthase subunit, PfaA	Unsaturated fatty acid biosynthesis
**FabR**	pfaB	7	2	omega-3 polyunsaturated fatty acid synthase subunit, PfaB	Unsaturated fatty acid biosynthesis
**FabR**	COG2030	6	1	Putative acyl dehydratase, COG2030	
**FabR**	pfaR	6	1	transcriptional regulator for synthesis of eicosapentaenoic acid, PfaR	Transcription regulation
**FabR**	pfaC	6	1	omega-3 polyunsaturated fatty acid synthase subunit, PfaC	Unsaturated fatty acid biosynthesis
**FabR**	desA	1	1	Fatty acid desaturase (EC 1.14.99.-)	Unsaturated fatty acid biosynthesis
**FabR**	fadE3	5	1	Acyl-CoA dehydrogenase, short-chain specific (EC 1.3.99.2)	Fatty acid degradation
**FabR**	pfaD	4	1	Enoyl-[acyl-carrier-protein] reductase [FMN] (EC 1.3.1.9), inferred for PFA pathway	Fatty acid biosynthesis
**FabR**	psrA	2	1	Predicted transcriptional regulator for fatty acid degradation PsrA, TetR family	Unsaturated fatty acid biosynthesis
**FabR**	fadE	1	1	Acyl-CoA dehydrogenase, short-chain specific (EC 1.3.99.2)	Fatty acid degradation
**FabR**	COG2072	3	2	probable flavin-containing monooxygenase, COG2072	
**FabR**	COG0300	2	1	probable short-chain dehydrogenase, COG0300	
**FadP**	fadP	24	3	Predicted transcriptional regulator for fatty acid degradation FadP, TetR family	Transcription regulation
**FadP**	etfA	23	3	Electron transfer flavoprotein alpha subunit	Electron transfer chain for fatty acid degradation
**FadP**	etfB	23	3	Electron transfer flavoprotein, beta subunit	Electron transfer chain for fatty acid degradation
**FadP**	acdH	23	3	Acyl-CoA dehydrogenase (EC 1.3.99.3)	Fatty acid degradation
**FadP**	fadA	22	3	3-ketoacyl-CoA thiolase (EC 2.3.1.16)	Fatty acid degradation

**FadP**	fadB	22	3	Enoyl-CoA hydratase (EC 4.2.1.17) / Delta(3)-cis-delta(2)-trans-enoyl-CoA isomerase (EC 5.3.3.8) / 3-hydroxyacyl-CoA dehydrogenase (EC 1.1.1.35) / 3-hydroxybutyryl-CoA epimerase (EC 5.1.2.3)	Fatty acid degradation
**FadP**	acdB	22	3	Enoyl-CoA hydratase (EC 4.2.1.17) / 3,2-trans-enoyl-CoA isomerase (EC 5.3.3.8) / 3-hydroxyacyl-CoA dehydrogenase (EC 1.1.1.35)	Fatty acid degradation
**FadP**	acdA	22	3	3-ketoacyl-CoA thiolase (EC 2.3.1.16) @ Acetyl-CoA acetyltransferase (EC 2.3.1.9)	Fatty acid degradation
**FadP**	etfD	21	3	Electron transfer flavoprotein-ubiquinone oxidoreductase (EC 1.5.5.1)	Electron transfer chain for fatty acid degradation
**FadP**	acdH2	20	3	Acyl-CoA dehydrogenase (EC 1.3.99.3)	Fatty acid degradation
**FadP**	echH	19	3	Enoyl-CoA hydratase (EC 4.2.1.17)	Fatty acid degradation
**FadP**	acdP	18	3	acyl-CoA dehydrogenase domain protein	Fatty acid degradation
**FadP**	acdQ	18	3	Acyl-CoA dehydrogenase (EC 1.3.99.-)	Fatty acid degradation
**FadP**	fadD1	13	2	Long-chain-fatty-acid--CoA ligase (EC 6.2.1.3)	Fatty acid degradation
**FadP**	maoC	13	2	Putative (R)-specific enoyl-CoA hydratase, MaoC- like	
**FadP**	echI	13	2	MaoC domain protein dehydratase	
**FadP**	pncA	13	2	Nicotinamidase (EC 3.5.1.19)	Nicotinate biosynthesis
**FadP**	fadD2	11	2	Long-chain-fatty-acid--CoA ligase (EC 6.2.1.3)	Fatty acid degradation
**FadP**	nppD	11	2	2-nitropropane dioxygenase NPD	
**FadP**	acdH3	12	3	Acyl-CoA dehydrogenase (EC 1.3.99.3)	Fatty acid degradation
**FadP**	paaG4	7	2	Enoyl-CoA hydratase (EC 4.2.1.17)	Fatty acid degradation
**FadP**	paaH1	7	2	3-hydroxybutyryl-CoA dehydrogenase, phenylacetic acid degradation	Fatty acid degradation
**FadP**	RSc1638	6	1	hypothetical protein	
**FadP**	BPSL1235	6	1	Phosphotransferase enzyme family protein	
**FadP**	bktB	6	1	3-ketoacyl-CoA thiolase (EC 2.3.1.16)	Fatty acid degradation
**FadP**	alkK	6	1	Medium-chain-fatty-acid-CoA ligase	Fatty acid degradation
**FadP**	Rsc1773	6	1	putative 4-hydroxybenzoyl-CoA thioesterase	
**FadP**	RSc1772	6	1	Alpha/beta hydrolase fold-3 domain protein	
**FadP**	RSc2046	6	1	conserved hypothetical protein, DUF1178	
**FadP**	BPSL1236	5	1	Putative phosphoglycerate mutase	Glycolysis
**FadP**	liuR	4	1	Predicted transcriptional regulator LiuR of leucine degradation pathway, MerR family	Transcription regulation
**FadP**	degV	4	1	conserved hypothetical protein, DegV family	
**FadP**	acsA	4	1	Acetyl-coenzyme A synthetase (EC 6.2.1.1)	Acetyl-coenzyme A synthetase
**FadP**	h16_A1555	4	1	conserved hypothetical protein, UPF0065	
**FadP**	Rmet_0145	3	1	TesB-like acyl-CoA thioesterase 2	
**FadP**	paaI	2	1	phenylacetic acid degradation protein PaaI	Fatty acid degradation
**FadP**	Rsc1774	2	1	GCN5-related N-acetyltransferase	
**FadP**	acdH4	1	1	Acyl-CoA dehydrogenase domain protein	Fatty acid degradation
**FadR**	fadL	50	5	Long-chain fatty acid transport protein	Fatty acid degradation
**FadR**	fadI	47	5	FadI component of anaerobic fatty acid oxidation complex	Fatty acid degradation
**FadR**	fadJ	46	5	FadJ component of anaerobic fatty acid oxidation complex	Fatty acid degradation
**FadR**	fadE	38	4	Acyl-CoA dehydrogenase, short-chain specific (EC 1.3.99.2)	Fatty acid degradation
**FadR**	fabA	26	3	3-hydroxydecanoyl-[acyl-carrier-protein] dehydratase (EC 4.2.1.60)	Fatty acid biosynthesis
**FadR**	fadH	25	3	2,4-dienoyl-CoA reductase [NADPH] (EC 1.3.1.34)	Fatty acid degradation
**FadR**	fadB	24	3	fatty oxidation complex, alpha subunit FadB	Fatty acid degradation
**FadR**	fadA	24	3	fatty oxidation complex, beta subunit FadA	Fatty acid degradation
**FadR**	fadR	26	2	Transcriptional regulator for fatty acid degradation FadR, GntR family	Transcription regulation
**FadR**	fadE2	16	2	Acyl-CoA dehydrogenase, short-chain specific (EC 1.3.99.2)	Fatty acid degradation
**FadR**	fabB	14	2	fatty oxidation complex, alpha subunit FadB	Fatty acid biosynthesis
**FadR**	fadD	13	2	Long-chain-fatty-acid--CoA ligase (EC 6.2.1.3)	Fatty acid degradation
**FadR**	SO0572	16	1	Enoyl-CoA hydratase (EC 4.2.1.17)	Fatty acid degradation
**FadR**	SO4716	13	1	Acetyltransferase, GNAT family	

**FadR**	plsB	8	1	Glycerol-3-phosphate acyltransferase (EC 2.3.1.15)	Glycerolipid metabolism
**FadR**	iclR	8	1	Acetate operon transcriptional repressor, IclR family	Transcription regulation
**FadR**	fadM	8	1	Long-chain acyl-CoA thioesterase FadM (EC=3.1.2.-)	Fatty acid degradation
**FadR**	yebV	8	1	hypothetical protein	
**FadR**	VC2105	6	1	Thioesterase/thiol ester dehydrase-isomerase	
**FadR**	tesB	4	1	Acyl-CoA thioesterase	Fatty acid degradation
**FadR**	acdB	2	1	Acyl-CoA dehydrogenase (EC 1.3.99.3)	Fatty acid degradation
**FadR**	COG0596	1	1	Predicted hydrolase/acyltransferase	
**GlcC**	glcE	23	7	Glycolate dehydrogenase (EC 1.1.99.14), FAD-binding subunit GlcE	Glycolate utilization
**GlcC**	glcF	22	7	Glycolate dehydrogenase (EC 1.1.99.14), iron-sulfur subunit GlcF	Glycolate utilization
**GlcC**	glcD	21	7	Glycolate dehydrogenase (EC 1.1.99.14), subunit GlcD	Glycolate utilization
**GlcC**	glcC	22	6	Glycolate utilization operon transcriptional activator GlcC	Transcription regulation
**GlcC**	glcG	13	4	Hypothetical protein GlcG in glycolate utilization operon	Glycolate utilization
**GlcC**	glcB	8	3	Malate synthase G (EC 2.3.3.9)	Tricarboxylic acid cycle
**GlcC**	glcA	1	1	Glycolate permease	Glycolate transport
**GlcC**	lldD	4	1	L-lactate dehydrogenase (EC 1.1.2.3)	Lactate utilization
**GlcC**	mlr6914	4	1	uncharacterized conserved membrane protein	
**GlcC**	lysR	3	1	Transcriptional regulator, LysR family, in glycolate utilization operon	Transcription regulation
**GlcC**	lldG	1	1	L-lactate dehydrogenase, subunit LldG	Lactate utilization
**GlcC**	lldE	1	1	L-lactate dehydrogenase, Fe-S oxidoreductase subunit LldE	Lactate utilization
**GlcC**	glcQ	1	1	Predicted TRAP-type glycolate transport system, small permease component	Glycolate transport
**GlcC**	glcM	1	1	Predicted TRAP-type glycolate transport system, large permease component	Glycolate transport
**GlcC**	lldP	2	1	L-lactate permease	Lactate utilization
**GlcC**	lldF	1	1	L-lactate dehydrogenase, Iron-sulfur cluster-binding subunit LldF	Lactate utilization
**GlcC**	glcP	1	1	Predicted TRAP-type glycolate transport system, periplasmic component	Glycolate transport
**GlcC**	NGR_c03940	1	1	hypothetical protein	
**GlcC**	ykgE	1	1	L-lactate dehydrogenase, Fe-S oxidoreductase subunit YkgE	Lactate utilization
**GlcC**	ykgF	1	1	L-lactate dehydrogenase, Iron-sulfur cluster-binding subunit YkgF	Lactate utilization
**GlcC**	ykgG	1	1	L-lactate dehydrogenase, hypothetical protein subunit YkgG	Lactate utilization
**HexR**	hexR	74	11	Central carbohydrate metabolism transcription regulator HexR, RpiR family	Transcription regulation
**HexR**	zwf	72	9	Glucose-6-phosphate 1-dehydrogenase (EC 1.1.1.49)	Pentose phosphate pathway
**HexR**	edd	55	9	Phosphogluconate dehydratase (EC 4.2.1.12)	Entner-Doudoroff pathway
**HexR**	eda	53	9	2-keto-3-deoxy-D-arabino-heptulosonate-7-phosphate synthase I alpha (EC 2.5.1.54) # AroA I alpha	Entner-Doudoroff pathway
**HexR**	pgl	53	8	6-phosphogluconolactonase (EC 3.1.1.31), eukaryotic type	Pentose phosphate pathway
**HexR**	glk	33	7	Glucokinase (EC 2.7.1.2)	Glycolysis
**HexR**	pykA	40	7	Pyruvate kinase (EC 2.7.1.40)	Glycolysis
**HexR**	gapA	28	5	NAD-dependent glyceraldehyde-3-phosphate dehydrogenase (EC 1.2.1.12)	Glycolysis
**HexR**	pgi	31	5	Glucose-6-phosphate isomerase (EC 5.3.1.9)	Glycolysis
**HexR**	ppc	20	4	Phosphoenolpyruvate carboxylase (EC 4.1.1.31)	Glycolysis
**HexR**	tal	23	5	Transaldolase (EC 2.2.1.2)	Pentose phosphate pathway
**HexR**	ppsA	20	3	Phosphoenolpyruvate synthase (EC 2.7.9.2)	Gluconeogenesis
**HexR**	aceB	25	3	Malate synthase (EC 2.3.3.9)	Tricarboxylic acid cycle
**HexR**	aceA	21	4	Isocitrate lyase (EC 4.1.3.1)	Tricarboxylic acid cycle
**HexR**	gapB	19	3	NADPH-dependent glyceraldehyde-3-phosphate dehydrogenase (EC 1.2.1.13)	Glycolysis
**HexR**	pflA	12	3	Pyruvate formate-lyase activating enzyme (EC 1.97.1.4)	Fermentation
**HexR**	aceE	10	2	Pyruvate dehydrogenase E1 component (EC 1.2.4.1)	Pyruvate metabolism
**HexR**	aceF	10	2	Dihydrolipoamide acetyltransferase component of pyruvate dehydrogenase complex (EC 2.3.1.12)	Pyruvate metabolism
**HexR**	gpmM	12	2	2,3-bisphosphoglycerate-independent phosphoglycerate mutase (EC 5.4.2.1)	Glycolysis
**HexR**	adhE	18	3	Alcohol dehydrogenase (EC 1.1.1.1)	Fermentation

**HexR**	pckA	10	4	Phosphoenolpyruvate carboxykinase [ATP] (EC 4.1.1.49)	Gluconeogenesis
**HexR**	grcA	12	2	Autonomous glycyl radical cofactor	
**HexR**	gltD	12	2	Glutamate synthase [NADPH] small chain (EC 1.4.1.13)	Glutamate biosynthesis
**HexR**	gltB	12	2	Glutamate synthase [NADPH] large chain (EC 1.4.1.13)	Glutamate biosynthesis
**HexR**	aldE	7	4	Aldose 1-epimerase	Glycolysis
**HexR**	tpiA	11	2	Triosephosphate isomerase (EC 5.3.1.1)	Glycolysis
**HexR**	pntB	11	2	NAD(P) transhydrogenase subunit beta (EC 1.6.1.2)	NAD metabolism
**HexR**	ptsG	8	2	PTS system, glucose-specific IIB component (EC 2.7.1.69) / PTS system, glucose-specific IIC component (EC 2.7.1.69)	Glucose transport
**HexR**	mtlA	10	2	PTS system, mannitol-specific IIC component (EC 2.7.1.69) / PTS system, mannitol-specific IIB component (EC 2.7.1.69) / PTS system, mannitol-specific IIA component (EC 2.7.1.69)	Mannitol utilization
**HexR**	pntA	10	2	NAD(P) transhydrogenase alpha subunit (EC 1.6.1.2)	NAD metabolism
**HexR**	pflB	7	2	Pyruvate formate-lyase (EC 2.3.1.54)	Fermentation
**HexR**	mtlR	9	2	Mannitol operon repressor	Transcription regulation
**HexR**	ptsI	7	2	Phosphoenolpyruvate-protein phosphotransferase of PTS system (EC 2.7.3.9)	Glucose transport
**HexR**	focA	7	2	Formate efflux transporter (TC 2.A.44 family)	Fermentation
**HexR**	ptsH	7	2	Phosphocarrier protein of PTS system	Glucose transport
**HexR**	mtlD	9	2	Mannitol-1-phosphate 5-dehydrogenase (EC 1.1.1.17)	Mannitol utilization
**HexR**	crr	7	2	PTS system, glucose-specific IIA component (EC 2.7.1.69)	Glucose transport
**HexR**	phk	16	1	Xylulose-5-phosphate phosphoketolase (EC 4.1.2.9)	Pentose phosphate pathway
**HexR**	hexR1	8	1	Central carbohydrate metabolism transcription regulator HexR, RpiR family	Transcription regulation
**HexR**	gapA2	16	1	glyceraldehyde-3-phosphate dehydrogenase, type I	Glycolysis
**HexR**	ybfA	10	1	Putative exported protein	
**HexR**	pepD	9	1	Aminoacyl-histidine dipeptidase (Peptidase D) (EC 3.4.13.3)	
**HexR**	deoD	14	1	Purine nucleoside phosphorylase (EC 2.4.2.1)	Nucleoside metabolism
**HexR**	gnd	14	1	6-phosphogluconate dehydrogenase, decarboxylating (EC 1.1.1.44)	Pentose phosphate pathway
**HexR**	nqrD	14	1	NADH:ubiquinone oxidoreductase, Na translocating, hydrophobic membrane protein NqrD	Electron transport chain
**HexR**	nqrC	14	1	NADH:ubiquinone oxidoreductase, Na translocating, gamma subunit	Electron transport chain
**HexR**	gltR	7	1	DNA-binding response regulator GltR, controls specific porins for the entry of glucose	Transcription regulation
**HexR**	nqrF	14	1	NADH:ubiquinone oxidoreductase, Na translocating, beta subunit	Electron transport chain
**HexR**	nqrA	14	1	NADH:ubiquinone oxidoreductase, Na translocating, alpha subunit	Electron transport chain
**HexR**	deoA	14	1	Thymidine phosphorylase (EC 2.4.2.4)	Nucleoside metabolism
**HexR**	nqrE	14	1	NADH:ubiquinone oxidoreductase, Na translocating, hydrophobic membrane protein NqrE	Electron transport chain
**HexR**	deoB	14	1	Phosphopentomutase (EC 5.4.2.7)	Nucleoside metabolism
**HexR**	nqrB	14	1	NADH:ubiquinone oxidoreductase, Na translocating, hydrophobic membrane protein NqrB	Electron transport chain
**HexR**	gltS	7	1	Integral membrane sensor signal transduction histidine kinase (EC 2.7.13.3), glucose catabolism cluster	Transcription regulation
**HexR**	ygaW	8	1	Putative inner membrane protein	
**HexR**	eno	4	3	Enolase (EC 4.2.1.11)	Glycolysis
**HexR**	gcvT	12	1	Aminomethyltransferase (glycine cleavage system T protein) (EC 2.1.2.10)	Glycine cleavage system
**HexR**	gcvP	12	1	Glycine dehydrogenase [decarboxylating] (glycine cleavage system P protein) (EC 1.4.4.2)	Glycine cleavage system
**HexR**	nupC	12	1	nucleoside transporter, NupC family	Nucleoside metabolism
**HexR**	gcvH	12	1	Glycine cleavage system H protein	Glycine cleavage system
**HexR**	nirB	7	1	Nitrite reductase [NAD(P)H] large subunit (EC 1.7.1.4)	Nitrogen metabolism
**HexR**	nirD	7	1	Nitrite reductase [NAD(P)H] small subunit (EC 1.7.1.4)	Nitrogen metabolism
**HexR**	pta	4	1	Phosphate acetyltransferase (EC 2.3.1.8)	Fermentation
**HexR**	mgsA	2	1	Methylglyoxal synthase (EC 4.2.3.3)	Methylglyoxal metabolism
**HexR**	gltA	2	1	Citrate synthase (si) (EC 2.3.3.1)	Tricarboxylic acid cycle

**HexR**	ackA	4	1	Acetate kinase (EC 2.7.2.1)	Fermentation
**HexR**	PF00248	2	1	Putative aldo/keto reductase	
**HexR**	SO1118	10	1	hypothetical protein	
**HexR**	cdd	10	1	cytidine deaminase	Nucleoside metabolism
**HexR**	glgX	6	1	Glycogen debranching enzyme (EC 3.2.1.-)	Glycogen utilization
**HexR**	pgk	3	2	Phosphoglycerate kinase (EC 2.7.2.3)	Glycolysis
**HexR**	glgA	5	1	Glycogen synthase, ADP-glucose transglucosylase (EC 2.4.1.21)	Glycogen utilization
**HexR**	adhB	8	1	alcohol dehydrogenase II	Fermentation
**HexR**	ldhA	3	1	D-lactate dehydrogenase (EC 1.1.1.28)	Fermentation
**HexR**	gntU	1	1	Low-affinity gluconate/H+ symporter GntU	Gluconate utilization
**HexR**	glgC	5	1	Glucose-1-phosphate adenylyltransferase (EC 2.7.7.27)	Glycogen utilization
**HexR**	mglB	3	1	Galactose/methyl galactoside ABC transport system, D-galactose-binding periplasmic protein MglB (TC 3.A.1.2.3)	Galactose transport
**HexR**	mcp1	8	1	methyl-accepting chemotaxis protein	
**HexR**	ptsHI	1	1	PTS system, glucose-specific IIA component (EC 2.7.1.69) / Phosphocarrier protein of PTS system / Phosphoenolpyruvate-protein phosphotransferase of PTS system (EC 2.7.3.9)	Glucose transport
**HexR**	gntK	1	1	Gluconokinase (EC 2.7.1.12)	Gluconate utilization
**HexR**	mglA	3	1	Galactose/methyl galactoside ABC transport system, ATP-binding protein MglA (EC 3.6.3.17)	Galactose transport
**HexR**	mglC	3	1	Galactoside transport system permease protein mglC (TC 3.A.1.2.3)	Galactose transport
**HexR**	cpsA	2	1	Capsular polysaccharide synthesis enzyme CpsA, sugar transferase	
**HexR**	glgP	2	1	Glycogen phosphorylase (EC 2.4.1.1)	Glycogen utilization
**HexR**	prpB	1	1	Carboxyphosphonoenolpyruvate phosphonomutase (EC 2.7.8.23)	
**HexR**	oxlT	1	1	Putative oxalate:formate antiporter	Tricarboxylic acid cycle
**HexR**	ugpC	1	1	SN-glycerol-3-phosphate transport ATP-binding protein UgpC (TC 3.A.1.1.3)	Glycerol-3-phosphate transport
**HexR**	glpT	2	1	Glycerol-3-phosphate transporter	Glycerol-3-phosphate transport
**HexR**	pykF	1	1	Pyruvate kinase (EC 2.7.1.40)	Glycolysis
**HexR**	lctP	3	1	L-lactate permease	Lactate utilization
**HexR**	gapN	2	1	NADP-dependent glyceraldehyde-3-phosphate dehydrogenase (EC 1.2.1.9)	Glycolysis
**HexR**	fba	1	1	Fructose-bisphosphate aldolase class II (EC 4.1.2.13)	Glycolysis
**HexR**	manC	1	1	Mannose-1-phosphate guanylyltransferase (GDP) (EC 2.7.7.22)	Mannose utilization
**HexR**	bkdA2	1	1	Branched-chain alpha-keto acid dehydrogenase, E1 component, beta subunit (EC 1.2.4.4)	Branched-chain amino acid degradation
**HexR**	bkdB	1	1	Dihydrolipoamide acyltransferase component of branched-chain alpha-keto acid dehydrogenase complex (EC 2.3.1.168)	Branched-chain amino acid degradation
**HexR**	bkdA1	1	1	Branched-chain alpha-keto acid dehydrogenase, E1 component, alpha subunit (EC 1.2.4.4)	Branched-chain amino acid degradation
**HexR**	nupC2	3	1	putative nucleoside transporter, NupC family	Nucleoside metabolism
**HexR**	mcp2	3	1	methyl-accepting chemotaxis sensory transducer	
**HexR**	PST_3493	1	1	Aldo/keto reductase	
**HexR**	dld	1	1	D-Lactate dehydrogenase (EC 1.1.2.5)	Lactate utilization
**HmgQ**	hpd	16	2	4-hydroxyphenylpyruvate dioxygenase (EC 1.13.11.27)	Tyrosine degradation
**HmgQ**	hmgA	14	1	Homogentisate 1,2-dioxygenase (EC 1.13.11.5)	Tyrosine degradation
**HmgQ**	hmgB	1	1	Maleylacetoacetate isomerase (EC 5.2.1.2)	Tyrosine degradation
**HmgQ**	hmgC	1	1	Fumarylacetoacetase (EC 3.7.1.2)	Tyrosine degradation
**HmgQ**	gloA	1	1	Predicted homogentisate dioxygenase, GloA family	Tyrosine degradation
**HmgQ**	hmgQ	16	1	Tyrosine degradation transcriptional regulator, LysR family	Transcription regulation
**HmgR**	hpd	1	1	4-hydroxyphenylpyruvate dioxygenase (EC 1.13.11.27)	Tyrosine degradation
**HmgR**	hmgA	5	1	Homogentisate 1,2-dioxygenase (EC 1.13.11.5)	Tyrosine degradation
**HmgR**	hmgB	5	1	Maleylacetoacetate isomerase (EC 5.2.1.2)	Tyrosine degradation
**HmgR**	hmgC	5	1	Fumarylacetoacetase (EC 3.7.1.2)	Tyrosine degradation
**HmgR**	COG2814	3	1	Predicted tyrosine transporter, COG2814 family	Tyrosine transport

**HmgR**	hmgR	5	1	Tyrosine degradation transcriptional regulator, IclR family	Transcription regulation
**HmgS**	hmgA	3	1	Homogentisate 1,2-dioxygenase (EC 1.13.11.5)	Tyrosine degradation
**HmgS**	hmgB	3	1	Maleylacetoacetate isomerase (EC 5.2.1.2)	Tyrosine degradation
**HmgS**	hmgS	3	1	Tyrosine degradation transcriptional regulator, MarR family	Transcription regulation
**HutC**	hutU	106	18	Urocanate hydratase (EC 4.2.1.49)	Histidine degradation
**HutC**	hutH	108	18	Histidine ammonia-lyase (EC 4.3.1.3)	Histidine degradation
**HutC**	hutI	108	18	Imidazolonepropionase (EC 3.5.2.7)	Histidine degradation
**HutC**	hutC	103	17	Histidine utilization repressor, GntR family	Transcription regulation
**HutC**	hutF	59	13	Formiminoglutamic iminohydrolase (EC 3.5.3.13)	Histidine degradation
**HutC**	hutG	53	13	N-formylglutamate deformylase (EC 3.5.1.68)	Histidine degradation
**HutC**	hutD	41	11	Conserved hypothetical protein related to histidine degradation	Histidine degradation
**HutC**	hutG2	32	10	Formiminoglutamase (EC 3.5.3.8)	Histidine degradation
**HutC**	hisT	18	6	Histidine transport protein (permease)	Histidine transport
**HutC**	hutH2	14	6	Histidine ammonia-lyase (EC:4.3.1.3)	Histidine degradation
**HutC**	COG3314	6	5	Predicted histidine uptake transporter	Histidine transport
**HutC**	COG1457 (CodB)	11	5	Permease, cytosine/purines, uracil, thiamine, allantoin family protein	Transport
**HutC**	hutX	8	4	Histidine ABC transporter, histidine-binding protein (TC 3.A.1)	Histidine transport
**HutC**	hutW	8	4	Histidine ABC transporter, permease protein (TC 3.A.1)	Histidine transport
**HutC**	hutV	8	4	Histidine ABC transporter, ATP-binding protein (TC 3.A.1)	Histidine transport
**HutC**	COG834 (HisJ)	7	4	ABC amino acid transporter, periplasmic binding protein	Histidine transport
**HutC**	hisC	6	4	Histidinol-phosphate aminotransferase	Histidine biosynthesis
**HutC**	COG1126 (GlnQ)	4	3	ABC amino acid transporter, ATPase component	Histidine transport
**HutC**	COG765 (HisM)	5	3	ABC amino acid transporter, permease component	Histidine transport
**HutC**	hisX	13	3	Putative histidine ABC transporter, substrate binding protein	Histidine transport
**HutC**	hisY	13	3	Putative histidine ABC transporter, permease protein	Histidine transport
**HutC**	hisZ	13	3	Putative histidine ABC transporter, ATPase protein	Histidine transport
**HutC**	Caul_2357	3	2	Optional hypothetical component of omp transporter	Transport
**HutC**	omp	3	2	TonB-dependent outer membrane transporter	Histidine transport?
**HutC**	COG5285	4	2	Phytanoyl-CoA dioxygenase	
**HutC**	COG4160 (ArtM)	2	2	ABC amino acid transporter, permease component	Transport
**HutC**	COG277(GlcD)	2	2	FAD linked oxidase domain protein	
**HutC**	COG1125 (OpuBA)	2	2	ABC proline/glycine/betaine transporter, ATPase component	Transport
**HutC**	COG1174 (OpuBB)	2	2	ABC proline/glycine/betaine transporter, permease component	Transport
**HutC**	COG1732 (OpuBC)	2	2	ABC proline/glycine/betaine transporter, periplasmic binding domain	Transport
**HutC**	COG2423	2	2	Predicted ornithine cyclodeaminase, mu-crystallin homolog (EC 4.3.1.12)	
**HutC**	COG3221 (PhnD)	2	2	ABC phosphate/phosphonate transporter, periplasmic binding component	Transport
**HutC**	hisD	4	2	Histidinol dehydrogenase (EC 1.1.1.23)	Histidine biosynthesis
**HutC**	hisI	2	1	Phosphoribosyl-AMP cyclohydrolase (EC 3.5.4.19) / Phosphoribosyl-ATP pyrophosphatase (EC 3.6.1.31)	Histidine biosynthesis
**HutC**	hisG	2	1	ATP phosphoribosyltransferase (EC 2.4.2.17)	Histidine biosynthesis
**HutC**	hisF	2	1	Imidazole glycerol phosphate synthase cyclase subunit (EC 4.1.3.-)	Histidine biosynthesis
**HutC**	hisA	2	1	Phosphoribosylformimino-5-aminoimidazole carboxamide ribotide isomerase (EC 5.3.1.16)	Histidine biosynthesis
**HutC**	hisH	2	1	Imidazole glycerol phosphate synthase amidotransferase subunit (EC 2.4.2.-)	Histidine biosynthesis
**HutC**	hisB	2	1	Histidinol-phosphatase (EC 3.1.3.15) / Imidazoleglycerol-phosphate dehydratase (EC 4.2.1.19)	Histidine biosynthesis
**HutC**	COG2814 (AraJ)	4	1	Putative histidine permease, major facilitator superfamily	Histidine transport
**HutC**	hisP	4	1	Histidine ABC transporter, ATP-binding protein HisP (TC 3.A.1.3.1)	Histidine transport
**HutC**	hisP2	4	1	Histidine ABC transporter, ATP-binding protein HisP (TC 3.A.1.3.1)	Histidine transport
**HutC**	COG1960 (CaiA)	1	1	Acyl-CoA dehydrogenase, short-chain specific (EC 1.3.99.2)	
**HutC**	COG1804 (CaiB)	1	1	CAIB/BAIF family protein	

**HutC**	hisJ2	4	1	Histidine ABC transporter, histidine-binding periplasmic protein precursor HisJ (TC 3.A.1.3.1)	Histidine transport
**HutC**	hisM	4	1	Histidine ABC transporter, permease protein HisM (TC 3.A.1.3.1)	Histidine transport
**HutC**	hisM2	4	1	Histidine ABC transporter, permease protein HisM (TC 3.A.1.3.1)	Histidine transport
**HutC**	hisQ	4	1	Histidine ABC transporter, permease protein HisQ (TC 3.A.1.3.1)	Histidine transport
**HutC**	hisQ2	4	1	Histidine ABC transporter, permease protein HisQ (TC 3.A.1.3.1)	Histidine transport
**HutC**	COG1414	3	1	Transcriptional regulator, IclR family	
**HutC**	COG2855	1	1	Predicted membrane protein	
**HutC**	COG3181	1	1	Uncharacterized conserved putative exported protein	
**HutC**	hisJ	2	1	Histidine ABC transporter, histidine-binding periplasmic protein precursor HisJ (TC 3.A.1.3.1)	Histidine transport
**HutC**	COG3842 (PotA)	1	1	ABC transporter, ATPase component	Transport
**HutC**	COG1176 (PotB)	1	1	ABC transporter, permease component	Transport
**HutC**	COG1177 (PotC)	1	1	ABC transporter, permease component	Transport
**HutC**	COG687 (PotD)	1	1	ABC transporter, periplasmic binding protein	Transport
**HutC**	COG1028 (FabG)	2	1	Short-chain alcohol dehydrogenase	
**HutC**	COG1063 (Tdh)	2	1	Zn-dependent dehydrogenase	
**HutC**	SMa0403	2	1	hypothetical protein	
**HutC**	COG596 (MhpC)	1	1	Putative hydrolases or acyltransferases (alpha/beta hydrolase superfamily)	
**HypR**	hypD	31	11	1-pyrroline-4-hydroxy-2-carboxylate deaminase (EC 3.5.4.22) # predicted	Hydroxyproline/proline degradation
**HypR**	hypR	32	10	Predicted regulator for proline and hydrohyproline utilization, GntR family	Transcription regulation
**HypR**	hypO	20	9	D-amino acid dehydrogenase (EC 1.4.99.1) family protein in hydroxy-L-proline catabolic cluster	Hydroxyproline/proline degradation
**HypR**	hypE	21	9	Proline racemase /4-hydroxyproline epimerase (EC 5.1.1.8)	Hydroxyproline/proline degradation
**HypR**	hypH	17	7	Ketoglutarate semialdehyde dehydrogenase (EC 1.2.1.26) # hydroxy-L-proline-inducible	Hydroxyproline/proline degradation
**HypR**	hypS	8	5	Putative L-lactate/Malate dehydrogenase	TCA cycle
**HypR**	hypY	9	4	Proline racemase /4-hydroxyproline epimerase (EC 5.1.1.8)	Hydroxyproline/proline degradation
**HypR**	COG2423	8	3	Predicted ornithine cyclodeaminase, mu-crystallin homolog	Proline degradation
**HypR**	hypP	4	3	Predicted hydroxyproline ABC transporter, permease protein	Hydroxyproline transport
**HypR**	hypM	4	3	Predicted hydroxyproline ABC transporter, substrate-binding protein	Hydroxyproline transport
**HypR**	hypN	4	3	Predicted hydroxyproline ABC transporter, permease protein	Hydroxyproline transport
**HypR**	hypQ	4	3	Predicted hydroxyproline ABC transporter, ATP-binding protein	Hydroxyproline transport
**HypR**	hypX	4	3	Putative citrate/isocitrate isomerase or aconitase ## predicted from clustering to proline racemase	TCA cycle
**HypR**	putA	18	2	Proline dehydrogenase (EC 1.5.99.8) (Proline oxidase) / Delta-1-pyrroline-5-carboxylate dehydrogenase (EC 1.5.1.12)	Proline degradation
**HypR**	prdP	8	2	Proline dipeptidase	Proline degradation
**HypR**	ampP	9	2	Xaa-Pro aminopeptidase (EC 3.4.11.9)	Proline degradation
**HypR**	omp	5	2	TonB-dependent receptor, collagen-binding surface protein	Proline transport?
**HypR**	colA2	9	2	Microbial collagenase, secreted (EC 3.4.24.3)	Collagen degradation
**HypR**	COG4663	2	1	TRAP-type C4-dicarboxylate transport system, periplasmic component	
**HypR**	COG3090	2	1	TRAP-type C4-dicarboxylate transport system, small permease component	
**HypR**	COG4664	2	1	TRAP-type C4-dicarboxylate transport system, large permease component	
**HypR**	hypD'	1	1	1-pyrroline-4-hydroxy-2-carboxylate deaminase (EC 3.5.4.22) # predicted	Hydroxyproline/proline degradation
**HypR**	hypH'	1	1	Ketoglutarate semialdehyde dehydrogenase (EC 1.2.1.26) # hydroxy-L-proline-inducible	Hydroxyproline/proline degradation
**HypR**	hypH'-2	1	1	Putative ketoglutarate semialdehyde dehydrogenase (EC 1.2.1.26) # hydroxy-L-proline-inducible	Hydroxyproline/proline degradation
**HypR**	EAM_2484	1	1	Oxidase	
**HypR**	COG446 (HcaD)	1	1	Putative oxidoreductase in 4-hydroxyproline catabolic gene cluster	
**HypR**	COG687 (PotD)	1	1	Probable binding protein component of ABC transporter	
**HypR**	VP1327	1	1	hypothetical protein	
**HypR**	COG2271 (UhpC)	1	1	Transporter, MFS superfamily	

**HypR**	hypT	1	1	Putative hydroxyproline transporter	Hydroxyproline transport
**HypR**	hypZ	1	1	OsmC/Ohr family protein	
**HypR**	COG531 (PotE)	1	1	Putative proline/hydroxyproline permease, PotE family	Hydroxyproline/proline transport
**HypR**	colA1	7	1	Microbial collagenase, secreted (EC 3.4.24.3)	Collagen degradation
**HypR**	pdtP	6	1	Predicted proline dipeptide/tripeptide permease, MFS family	Proline transport
**HypR**	ATW7_13133	1	1	hypothetical protein	
**HypR**	ypdF	1	1	Aminopeptidase YpdF (MP-, MA-, MS-, AP-, NP- specific)	Collagen degradation
**HypR**	hypA	1	1	Predicted hydroxyproline TRAP-type transport system, small permease component	Hydroxyproline transport
**HypR**	hypB	1	1	Predicted hydroxyproline TRAP-type transport system, large permease component	Hydroxyproline transport
**HypR**	hypC	1	1	Predicted hydroxyproline TRAP-type transport system, periplasmic component	Hydroxyproline transport
**HypR**	colA3	4	1	Microbial collagenase, secreted (EC 3.4.24.3)	Collagen degradation
**HypR**	omp2	1	1	TonB-dependent receptor	
**LiuQ**	liuD	14	3	Methylcrotonyl-CoA carboxylase carboxyl transferase subunit (EC 6.4.1.4)	Branched_chain amino acid degradation
**LiuQ**	liuB	14	3	Methylcrotonyl-CoA carboxylase biotin-containing subunit (EC 6.4.1.4)	Branched_chain amino acid degradation
**LiuQ**	liuQ	12	3	Predicted branched-chain amino acid degradation regulator LiuQ, TetR family	Branched_chain amino acid degradation
**LiuQ**	liuA	11	3	Isovaleryl-CoA dehydrogenase (EC 1.3.99.10)	Branched_chain amino acid degradation
**LiuQ**	liuC	11	3	Methylglutaconyl-CoA hydratase (EC 4.2.1.18)	Branched_chain amino acid degradation
**LiuQ**	aacS	4	1	AMP-dependent synthetase and ligase	Branched_chain amino acid degradation
**LiuQ**	liuE	2	2	Hydroxymethylglutaryl-CoA lyase (EC 4.1.3.4)	Branched_chain amino acid degradation
**LiuQ**	ivd2	1	1	Isovaleryl-CoA dehydrogenase (EC 1.3.99.10)	Branched_chain amino acid degradation
**LiuQ**	liuQ2	1	1	Predicted transcriptional regulator LiuQ of leucine degradation pathway, TetR family	Transcription regulation
**LiuQ**	atuB	1	1	Short-chain dehydrogenase/reductase SDR	
**LiuQ**	liuQ1	1	1	Predicted transcriptional regulator LiuQ of leucine degradation pathway, TetR family	Transcription regulation
**LiuQ**	acsA	1	1	Acetyl-coenzyme A synthetase (EC 6.2.1.1)	Acyl-coenzyme A synthetase
**LiuR**	etfA	49	10	Electron transfer flavoprotein, alpha subunit	Electron transfer chain for branched-chain amino acid degradation
**LiuR**	etfB	48	9	Electron transfer flavoprotein, beta subunit	Electron transfer chain for branched-chain amino acid degradation
**LiuR**	ivdA	54	10	Acetyl-CoA C-acyltransferase (EC 2.3.1.16) @ Acetyl-CoA acetyltransferase (EC 2.3.1.9)	Branched-chain amino acid degradation
**LiuR**	ivdC	47	9	Branched-chain acyl-CoA dehydrogenase (EC 1.3.99.12)	Branched-chain amino acid degradation
**LiuR**	liuA	65	11	Isovaleryl-CoA dehydrogenase (EC 1.3.99.10); Butyryl-CoA dehydrogenase (EC 1.3.99.2)	Branched-chain amino acid degradation
**LiuR**	liuB	55	10	Methylcrotonyl-CoA carboxylase biotin-containing subunit (EC 6.4.1.4)	Branched-chain amino acid degradation
**LiuR**	liuC	54	10	Methylglutaconyl-CoA hydratase (EC 4.2.1.18)	Branched-chain amino acid degradation
**LiuR**	liuD	46	9	Methylcrotonyl-CoA carboxylase carboxyl transferase subunit (EC 6.4.1.4)	Branched-chain amino acid degradation
**LiuR**	liuE	49	9	Hydroxymethylglutaryl-CoA lyase (EC 4.1.3.4)	Branched-chain amino acid degradation
**LiuR**	liuR	90	13	Predicted transcriptional regulator LiuR of leucine degradation pathway, MerR family	Transcriptional regulator
**LiuR**	aacS2	1	1	Acetoacetyl-CoA synthetase (EC 6.2.1.16) / Long-chain-fatty-acid--CoA ligase (EC 6.2.1.3)	Branched-chain amino acid degradation
**LiuR**	acdA2	2	2	3-ketoacyl-CoA thiolase (EC 2.3.1.16) @ Acetyl-CoA acetyltransferase (EC 2.3.1.9)	Branched-chain amino acid degradation
**LiuR**	acdA3	6	2	3-ketoacyl-CoA thiolase (EC 2.3.1.16) @ Acetyl-CoA acetyltransferase (EC 2.3.1.9)	Branched-chain amino acid degradation
**LiuR**	acdB2	1	1	Enoyl-CoA hydratase (EC 4.2.1.17)	Branched-chain amino acid degradation
**LiuR**	acdB3	1	1	Enoyl-CoA hydratase (EC 4.2.1.17)	Branched-chain amino acid degradation
**LiuR**	acdB4	2	1	Enoyl-CoA hydratase [valine degradation] (EC 4.2.1.17)	Branched-chain amino acid degradation
**LiuR**	acdB5	1	1	Enoyl-CoA hydratase (EC 4.2.1.17)	Branched-chain amino acid degradation
**LiuR**	acdH3	3	1	Acyl-CoA dehydrogenase, short-chain specific (EC 1.3.99.2)	Branched-chain amino acid degradation
**LiuR**	acdH4	1	1	Acyl-CoA dehydrogenase (EC 1.3.99.3)	Branched-chain amino acid degradation
**LiuR**	acdH5	1	1	Acyl-CoA dehydrogenase, short-chain specific (EC 1.3.99.2)	Branched-chain amino acid degradation

**LiuR**	aceA	1	1	isocitrate lyase	Tricarboxylic acid cycle
**LiuR**	acs	2	1	acyl-CoA synthase	Acyl-coenzyme A synthetase
**LiuR**	acsA	2	1	Acyl-coenzyme A synthetases/AMP-(fatty) acid ligases	Acyl-coenzyme A synthetase
**LiuR**	ahpD	2	1	alkylhydroperoxidase AhpD family core domain protein	
**LiuR**	badI	1	1	2-ketocyclohexanecarboxyl-CoA hydrolase (EC 4.1.3.36)	
**LiuR**	csgA	1	1	Short-chain dehydrogenase/reductase SDR(EC:1.1.1.184)	
**LiuR**	Daci_5984	1	1	Alpha/beta hydrolase fold (EC 3.8.1.5)	
**LiuR**	dcp	2	1	Peptidyl-dipeptidase dcp (EC 3.4.15.5)	
**LiuR**	ebA6516	1	1	Enoyl-CoA hydratase (EC 4.2.1.17)	Enoyl-CoA hydratase
**LiuR**	eftB	1	1	Electron transfer flavoprotein, beta subunit	Electron transfer chain for branched-chain amino acid degradation
**LiuR**	etfD1	1	1	Electron transfer flavoprotein-ubiquinone oxidoreductase (EC 1.5.5.1)	Electron transfer chain for branched-chain amino acid degradation
**LiuR**	etfD2	1	1	Electron transfer flavoprotein-ubiquinone oxidoreductase (EC 1.5.5.1)	Electron transfer chain for branched-chain amino acid degradation
**LiuR**	fabG	2	2	3-oxoacyl-[acyl-carrier protein] reductase (EC 1.1.1.100)	Fatty acid biosynthesis
**LiuR**	fadA	1	1	acetyl-CoA acyltransferase	Fatty acid degradation
**LiuR**	fadB	1	1	3-hydroxyacyl-CoA dehydrogenase	Fatty acid degradation
**LiuR**	fadL	1	1	Long-chain fatty acid transport protein	Fatty acid degradation
**LiuR**	fpr	1	1	Flavodoxin reductases (ferredoxin-NADPH reductases) family 1	
**LiuR**	h16_A0164	1	1	Metallo-beta-lactamase family protein	
**LiuR**	h16_A0171	1	1	Dehydrogenases with different specificities (related to short-chain alcohol dehydrogenases)	
**LiuR**	h16_A0173	1	1	2-Hydroxychromene-2-carboxylate isomerase	
**LiuR**	h16_A0174	1	1	Putative phosphatase YieH	
**LiuR**	h16_A0175	1	1	hypothetical protein	
**LiuR**	h16_A0176	1	1	Maleylacetoacetate isomerase (EC 5.2.1.2) / Glutathione S-transferase	
**LiuR**	h16_A0178	1	1	Dienelactone hydrolase or related enzyme	
**LiuR**	hit	3	1	Bis(5'-nucleosyl)-tetraphosphatase (asymmetrical) (EC 3.6.1.17)	
**LiuR**	IL0880	2	1	Sensory box/GGDEF family protein	
**LiuR**	ivdA1	1	1	3-ketoacyl-CoA thiolase [isoleucine degradation] (EC 2.3.1.16)	Branched-chain amino acid degradation
**LiuR**	ivdA2	1	1	3-ketoacyl-CoA thiolase [isoleucine degradation] (EC 2.3.1.16)	Branched-chain amino acid degradation
**LiuR**	ivdB1	1	1	Methylmalonate-semialdehyde dehydrogenase (EC 1.2.1.27)	Branched-chain amino acid degradation
**LiuR**	ivdB2	1	1	Methylmalonate-semialdehyde dehydrogenase (EC 1.2.1.27)	Branched-chain amino acid degradation
**LiuR**	ivdC1	1	1	Branched-chain acyl-CoA dehydrogenase (EC 1.3.99.12)	Branched-chain amino acid degradation
**LiuR**	ivdC2	1	1	Branched-chain acyl-CoA dehydrogenase (EC 1.3.99.12)	Branched-chain amino acid degradation
**LiuR**	ivdD1	1	1	3-hydroxyisobutyryl-CoA hydrolase (EC 3.1.2.4)	Branched-chain amino acid degradation
**LiuR**	ivdD2	1	1	3-hydroxyisobutyryl-CoA hydrolase (EC 3.1.2.4)	Branched-chain amino acid degradation
**LiuR**	ivdE1	1	1	Enoyl-CoA hydratase [valine degradation] (EC 4.2.1.17) / Enoyl-CoA hydratase [isoleucine degradation] (EC 4.2.1.17)	Branched-chain amino acid degradation
**LiuR**	ivdE2	1	1	Enoyl-CoA hydratase [valine degradation] (EC 4.2.1.17) / Enoyl-CoA hydratase [isoleucine degradation] (EC 4.2.1.17)	Branched-chain amino acid degradation
**LiuR**	ivdG1	1	1	3-hydroxyacyl-CoA dehydrogenase [isoleucine degradation] (EC 1.1.1.35)	Branched-chain amino acid degradation
**LiuR**	ivdG2	1	1	3-hydroxyacyl-CoA dehydrogenase [isoleucine degradation] (EC 1.1.1.35)	Branched-chain amino acid degradation
**LiuR**	lcfA	1	1	Long-chain-fatty-acid--CoA ligase (EC 6.2.1.3)	Fatty acid degradation
**LiuR**	livF	4	4	Branched-chain amino acid transport ATP-binding protein LivF (TC 3.A.1.4.1)	Branched-chain amino acid transport
**LiuR**	livG	4	4	Branched-chain amino acid transport ATP-binding protein LivG (TC 3.A.1.4.1)	Branched-chain amino acid transport
**LiuR**	livH	4	4	Branched-chain amino acid transport permease protein LivH (TC 3.A.1.4.1)	Branched-chain amino acid transport
**LiuR**	livK	6	5	Branched-chain amino acid transport substrate-binding protein LivK (TC 3.A.1.4.1)	Branched-chain amino acid transport
**LiuR**	livM	4	4	Branched-chain amino acid transport system permease protein LivM (TC 3.A.1.4.1)	Branched-chain amino acid transport
**LiuR**	mmgB	1	1	3-hydroxybutyryl-CoA dehydrogenase (EC 1.1.1.157)	Branched-chain amino acid degradation
**LiuR**	mmgC	1	1	Acyl-CoA dehydrogenase, short-chain specific (EC 1.3.99.2)	

**LiuR**	paaG	2	2	Enoyl-CoA hydratase/isomerase	
**LiuR**	paaH1	3	1	3-hydroxybutyryl-CoA dehydrogenase (EC 1.1.1.157)	Branched-chain amino acid degradation
**LiuR**	paaI	10	3	Phenylacetic acid degradation-related protein	
**LiuR**	paaK	2	2	Phenylacetate-coenzyme A ligase (EC 6.2.1.30)	
**LiuR**	Rru_A1944	1	1	2-hydroxychromene-2-carboxylate isomerase (EC 5.3.99.-)	
**LiuR**	RSc0258	1	1	Beta-lactamase domain protein	
**LiuR**	RSc0259	1	1	Protein of unknown function DUF1289	
**LiuR**	RSc0281	1	1	Metallo-beta-lactamase family protein	
**LiuR**	serA	2	1	D-3-phosphoglycerate dehydrogenase (EC 1.1.1.95)	Serine biosynthesis
**LiuR**	SMc00041	5	1	hypothetical protein	
**LiuR**	tyrP	1	1	Tyrosine-specific transport protein (HAAAP family)	
**LiuR**	tyrR	7	1	aromatic amino acid biosynthesis/transport transciptional regulator	Transcriptional regulator
**LiuR**	yciK	3	1	Oxidoreductase, short-chain dehydrogenase/reductase family	
**LiuR**	aacS	12	5	Acetoacetyl-CoA synthetase [leucine] (EC 6.2.1.16)	Branched-chain amino acid degradation
**LiuR**	acdA	28	5	Enoyl-CoA hydratase (EC 4.2.1.17)	Branched-chain amino acid degradation
**LiuR**	acdB	27	5	3-ketoacyl-CoA thiolase (EC 2.3.1.16) @ Acetyl-CoA acetyltransferase (EC 2.3.1.9)	Branched-chain amino acid degradation
**LiuR**	acdH	43	8	Acyl-CoA dehydrogenase (EC 1.3.99.3)	Branched-chain amino acid degradation
**LiuR**	acdH2	14	4	Acyl-CoA dehydrogenase (EC 1.3.99.3)	Branched-chain amino acid degradation
**LiuR**	acdL	7	4	Acyl-CoA dehydrogenase, long-chain specific, mitochondrial precursor (EC 1.3.99.13)	Branched-chain amino acid degradation
**LiuR**	acdL2	6	3	Acyl-CoA dehydrogenase, long-chain specific, mitochondrial precursor (EC 1.3.99.13)	Branched-chain amino acid degradation
**LiuR**	acdP	4	1	Acyl-CoA dehydrogenase family protein	Branched-chain amino acid degradation
**LiuR**	acdQ	4	1	Acyl-CoA dehydrogenases	Branched-chain amino acid degradation
**LiuR**	aceB	16	1	malate synthase A	Tricarboxylic acid cycle
**LiuR**	aceK	17	3	Isocitrate dehydrogenase phosphatase (EC 2.7.11.5)/kinase (EC 3.1.3.-)	Tricarboxylic acid cycle
**LiuR**	atuC	1	1	Geranyl-CoA carboxylase carboxyl transferase subunit	Branched-chain amino acid degradation
**LiuR**	atuD	1	1	Isovaleryl-CoA dehydrogenase (EC 1.3.99.10)	Branched-chain amino acid degradation
**LiuR**	atuE	1	1	Isohexenylglutaconyl-CoA hydratase	Branched-chain amino acid degradation
**LiuR**	atuF	1	1	Methylcrotonyl-CoA carboxylase biotin-containing subunit (EC 6.4.1.4)	Branched-chain amino acid degradation
**LiuR**	bkdA1	25	3	Branched-chain alpha-keto acid dehydrogenase, E1 component, alpha subunit (EC 1.2.4.4)	Branched-chain amino acid degradation
**LiuR**	bkdA2	25	3	Branched-chain alpha-keto acid dehydrogenase, E1 component, beta subunit (EC 1.2.4.4)	Branched-chain amino acid degradation
**LiuR**	bkdB	25	3	Dihydrolipoamide acyltransferase component of branched-chain alpha-keto acid dehydrogenase complex (EC 2.3.1.168)	Branched-chain amino acid degradation
**LiuR**	cah	19	4	Carbonic anhydrase (EC 4.2.1.1)	Carbonic anhydrase
**LiuR**	echH	7	1	Enoyl-CoA hydratase (EC 4.2.1.17)	Branched-chain amino acid degradation
**LiuR**	etfD	23	5	Electron transfer flavoprotein-ubiquinone oxidoreductase (EC 1.5.5.1)	Electron transfer chain for branched-chain amino acid degradation
**LiuR**	fadD	9	4	Long-chain-fatty-acid--CoA ligase (EC 6.2.1.3)	Fatty acid degradation
**LiuR**	gltB	18	2	Glutamate synthase [NADPH] large chain (EC 1.4.1.13)	Glutamate biosynthesis
**LiuR**	gltD	18	2	Glutamate synthase [NADPH] small chain (EC 1.4.1.13)	Glutamate biosynthesis
**LiuR**	hbdA	6	1	3-hydroxybutyryl-CoA dehydrogenase (EC 1.1.1.157)	Branched-chain amino acid degradation
**LiuR**	ivdB	28	5	Methylmalonate-semialdehyde dehydrogenase (EC 1.2.1.27)	Branched-chain amino acid degradation
**LiuR**	ivdD	27	5	3-hydroxyisobutyryl-CoA hydrolase (EC 3.1.2.4)	Branched-chain amino acid degradation
**LiuR**	ivdE	31	6	Enoyl-CoA hydratase [valine degradation] (EC 4.2.1.17)	Branched-chain amino acid degradation
**LiuR**	ivdF	32	6	3-hydroxyisobutyrate dehydrogenase (EC 1.1.1.31)	Branched-chain amino acid degradation
**LiuR**	ivdG	31	6	3-hydroxybutyryl-CoA dehydrogenase (EC 1.1.1.157)	Branched-chain amino acid degradation
**LiuR**	ldh	21	3	Leucine dehydrogenase (EC 1.4.1.9)	Branched-chain amino acid degradation
**LiuR**	liuF	19	2	Succinyl-CoA:3-ketoacid-coenzyme A transferase subunit A (EC 2.8.3.5)	Branched-chain amino acid degradation
**LiuR**	liuG	19	2	Succinyl-CoA:3-ketoacid-coenzyme A transferase subunit B (EC 2.8.3.5)	Branched-chain amino acid degradation

**LiuR**	liuR1	10	2	Predicted transcriptional regulator LiuR of leucine degradation pathway, MerR family	
**LiuR**	liuR2	5	4	Predicted transcriptional regulator LiuR of leucine degradation pathway, MerR family	
**LiuR**	mcm	9	2	Methylmalonyl-CoA mutase (EC 5.4.99.2)	Branched-chain amino acid degradation
**LiuR**	mdh	9	1	Malate dehydrogenase (EC 1.1.1.37)	Tricarboxylic acid cycle
**LiuR**	paaH	17	4	3-hydroxybutyryl-CoA dehydrogenase (EC 1.1.1.157)	Branched-chain amino acid degradation
**LiuR**	PF03060	6	1	Dioxygenases related to 2-nitropropane dioxygenase	
**LiuR**	PF04828	11	1	Glutathione S-transferase, unnamed subgroup (EC 2.5.1.18)	
**LiuR**	PF09917	6	1	Protein of unknown function, PF09917	
**LiuR**	prpB	2	1	Methylisocitrate lyase (EC 4.1.3.30)	Propionate metabolism
**LiuR**	prpC	2	1	2-methylcitrate synthase (EC 2.3.3.5)	Propionate metabolism
**LiuR**	prpD	2	1	2-methylcitrate dehydratase (EC 4.2.1.79)	Propionate metabolism
**LiuR**	Rfer_2814	1	1	protein of unknown function DUF849	
**LiuR**	sucA	9	1	2-oxoglutarate dehydrogenase E1 component (EC 1.2.4.2)	Tricarboxylic acid cycle
**LiuR**	sucB	8	1	Dihydrolipoamide succinyltransferase component (E2) of 2-oxoglutarate dehydrogenase complex (EC 2.3.1.61)	Tricarboxylic acid cycle
**LiuR**	sucC	9	1	Succinyl-CoA ligase [ADP-forming] beta chain (EC 6.2.1.5)	Tricarboxylic acid cycle
**LiuR**	sucD	9	1	Succinyl-CoA ligase [ADP-forming] alpha chain (EC 6.2.1.5)	Tricarboxylic acid cycle
**LiuR**	thrA	15	1	aspartate kinase	Threonine biosynthesis
**LiuR**	thrB	15	1	homoserine kinase	Threonine biosynthesis
**LiuR**	thrC	15	1	threonine synthase	Threonine biosynthesis
**LldR**	lldR	54	13	Lactate-responsive regulator LldR, GntR family	Transcription regulation
**LldR**	lldP	41	12	L-lactate permease	Lactate transport
**LldR**	dld	23	8	D-Lactate dehydrogenase (EC 1.1.2.5)	Lactate utilization
**LldR**	lldG	29	9	L-lactate dehydrogenase, hypothetical protein subunit LldG	Lactate utilization
**LldR**	lldD	21	7	L-lactate dehydrogenase (EC 1.1.2.3)	Lactate utilization
**LldR**	lldE	26	8	L-lactate dehydrogenase, Fe-S oxidoreductase subunit	Lactate utilization
**LldR**	lldF	25	8	L-lactate dehydrogenase, iron-sulfur cluster-binding subunit	Lactate utilization
**LldR**	glcF	5	2	Glycolate dehydrogenase (EC 1.1.99.14), iron-sulfur subunit GlcF	Glycolate utilization
**LldR**	glcD	5	2	Glycolate dehydrogenase (EC 1.1.99.14), subunit GlcD	Glycolate utilization
**LldR**	lldEF	4	1	L-lactate dehydrogenase, Fe-S oxidoreductase subunit LldE / L-lactate dehydrogenase, Iron-sulfur cluster-binding subunit LldF	Lactate utilization
**LldR**	lldX	5	1	predicted lactate permease, DUF81 family	Lactate transport
**LldR**	glcE	1	1	Glycolate dehydrogenase (EC 1.1.99.14), FAD-binding subunit GlcE	Glycolate utilization
**LldR**	glcG	1	1	Hypothetical protein GlcG in glycolate utilization operon	Glycolate utilization
**LldR**	glcB	1	1	Malate synthase G (EC 2.3.3.9)	Tricarboxylic acid cycle
**MetJ**	metA	50	5	Homoserine O-succinyltransferase (EC 2.3.1.46)	Methionine biosynthesis
**MetJ**	metB	46	6	Cystathionine gamma-synthase (EC 2.5.1.48)	Methionine biosynthesis
**MetJ**	metE	44	6	5-methyltetrahydropteroyltriglutamate--homocysteine methyltransferase (EC 2.1.1.14)	Methionine biosynthesis
**MetJ**	metF	43	6	5,10-methylenetetrahydrofolate reductase (EC 1.5.1.20)	Methionine biosynthesis
**MetJ**	metH	36	4	5-methyltetrahydrofolate--homocysteine methyltransferase (EC 2.1.1.13)	Methionine biosynthesis
**MetJ**	metI	36	4	Methionine ABC transporter permease protein	Methionine transport
**MetJ**	metJ	59	6	S-adenosylmethionine-responsive transcriptional repressor MetJ	Transcription regulation
**MetJ**	metK	55	6	S-adenosylmethionine synthetase (EC 2.5.1.6)	Methionine biosynthesis
**MetJ**	metL	48	5	Aspartokinase (EC 2.7.2.4) / Homoserine dehydrogenase (EC 1.1.1.3)	Methionine biosynthesis
**MetJ**	metN	37	4	Methionine ABC transporter ATP-binding protein	Methionine transport
**MetJ**	metQ	38	4	Methionine ABC transporter substrate-binding protein	Methionine transport
**MetJ**	metR	44	5	Homocysteine-responsive transcriptional regulator of methionine metabolism, LysR family	Transcription regulation
**MetJ**	metT	32	4	Methionine transporter MetT, NhaC antiporter family	Methionine transport
**MetJ**	ahpC	5	1	Alkyl hydroperoxide reductase protein C (EC 1.6.4.-)	

**MetJ**	ahpF	3	1	Alkyl hydroperoxide reductase protein F (EC 1.6.4.-)	
**MetJ**	ASA_2534	1	1	Erythronate-4-phosphate dehydrogenase (EC 1.1.1.290)	
**MetJ**	asd	1	1	Aspartate-semialdehyde dehydrogenase (EC 1.2.1.11)	Methionine biosynthesis
**MetJ**	btuB2	1	1	TonB-dependent outer membrane transporter for vitamin B12	Vitamin B12 transport
**MetJ**	btuC	1	1	Vitamin B12 ABC transporter, permease component BtuC	Vitamin B12 transport
**MetJ**	btuD	1	1	Vitamin B12 ABC transporter, ATPase component BtuD	Vitamin B12 transport
**MetJ**	btuF	1	1	Vitamin B12 ABC transporter, B12-binding component BtuF	Vitamin B12 transport
**MetJ**	CKO_03982	1	1	hypothetical protein	
**MetJ**	COG0235	3	1	Ribulose-5-phosphate 4-epimerase and related epimerases and aldolases	
**MetJ**	COG4948	3	1	L-alanine-DL-glutamate epimerase and related enzymes of enolase superfamily	
**MetJ**	COG5276	4	1	Hypothetical protein, COG5276 family	
**MetJ**	folE	3	1	GTP cyclohydrolase I (EC 3.5.4.16) type 1	
**MetJ**	mccA	3	1	Cystathionine beta-synthase (EC 4.2.1.22)	Methionine biosynthesis
**MetJ**	mccB	3	1	Cystathionine gamma-lyase (EC 4.4.1.1)	Methionine biosynthesis
**MetJ**	mdeA2	3	1	Methionine gamma-lyase (EC 4.4.1.11)	Methionine biosynthesis
**MetJ**	metC	12	2	Cystathionine beta-lyase (EC 4.4.1.8)	Methionine biosynthesis
**MetJ**	metE2	8	2	Methionine synthase II, vitamin-B12 independent (EC 2.1.1.14)	Methionine biosynthesis
**MetJ**	metF-II	4	1	5,10-methylenetetrahydrofolate reductase, non-orthologous isozyme (EC 1.5.1.20)	Methionine biosynthesis
**MetJ**	metQ2	5	3	Methionine ABC transporter substrate-binding protein	Methionine transport
**MetJ**	mmuM	11	4	Homocysteine S-methyltransferase (EC 2.1.1.10)	Methionine biosynthesis
**MetJ**	mmuP	10	3	S-methylmethionine transporter	Methionine transport
**MetJ**	mtnA	6	1	Methylthioribose-1-phosphate isomerase (EC 5.3.1.23)	Methylthioribose recycling
**MetJ**	mtnB	8	2	Methylthioribulose-1-phosphate dehydratase (EC 4.2.1.109)	Methylthioribose recycling
**MetJ**	mtnC	7	2	2,3-diketo-5-methylthiopentyl-1-phosphate enolase-phosphatase (EC 3.1.3.77)	Methylthioribose recycling
**MetJ**	mtnD	7	2	1,2-dihydroxy-3-keto-5-methylthiopentene dioxygenase (EC 1.13.11.54)	Methylthioribose recycling
**MetJ**	mtnE	7	1	Methionine aminotransferase	Methylthioribose recycling
**MetJ**	mtnK	7	2	5-methylthioribose kinase (EC 2.7.1.100)	Methylthioribose recycling
**MetJ**	mtnX	5	1	Predicted methylthioribose ABC transporter, ATP-binding protein	Methylthioribose recycling
**MetJ**	mtnY	5	1	Predicted methylthioribose ABC transporter, permease protein	Methylthioribose recycling
**MetJ**	mtnZ	5	1	Predicted methylthioribose ABC transporter, substrate-binding protein	Methylthioribose recycling
**MetJ**	pcbC	3	1	putative 2OG-Fe(II) oxygenase	
**MetJ**	PCNPT3_05874	1	1	Homoserine/homoserine lactone efflux protein	
**MetJ**	pduO	1	1	Cob(I)alamin adenosyltransferase (EC 2.5.1.17)	Vitamin B12 biosynthesis
**MetJ**	PF08908	2	1	Protein of unknown function DUF1852	
**MetJ**	serA	4	1	D-3-phosphoglycerate dehydrogenase (EC 1.1.1.95)	Serine biosynthesis
**MetJ**	thrA	3	1	Aspartokinase (EC 2.7.2.4) / Homoserine dehydrogenase (EC 1.1.1.3)	Threonine biosynthesis
**MetJ**	thrB	3	1	Homoserine kinase (EC 2.7.1.39)	Threonine biosynthesis
**MetJ**	thrC	3	1	Threonine synthase (EC 4.2.3.1)	Threonine biosynthesis
**MetJ**	ybdH	3	1	Uncharacterized oxidoreductase YbdH	
**MetJ**	btuB	30	5	TonB-dependent outer membrane transporter for vitamin B12	Vitamin B12 transport
**MetJ**	COG3126	14	1	Lipoprotein-related protein	
**MetJ**	csd	7	1	Cysteine desulfurase (EC 2.8.1.7)	Methionine metabolism
**MetJ**	metX	10	2	Homoserine O-acetyltransferase (EC 2.3.1.31)	Methionine biosynthesis
**MetJ**	metY	18	3	O-acetylhomoserine sulfhydrylase (EC 2.5.1.49) / O-succinylhomoserine sulfhydrylase (EC 2.5.1.48)	Methionine biosynthesis
**MetJ**	msrA	19	2	Peptide methionine sulfoxide reductase MsrA (EC 1.8.4.11)	Methionine metabolism
**MetJ**	mtsA	8	1	Substrate-specific component MtsA of methionine-regulated ECF transporter	Methionine transport
**MetJ**	mtsB	8	1	Duplicated ATPase component MtsB of energizing module of methionine-regulated ECF transporter	Methionine transport
**MetJ**	mtsC	8	1	Transmembrane component MtsC of energizing module of methionine-regulated ECF transporter	Methionine transport

**MetR**	metR	111	14	Homocysteine-responsive transcriptional regulator of methionine metabolism, LysR family	Transcription regulation
**MetR**	metE	88	12	5-methyltetrahydropteroyltriglutamate--homocysteine methyltransferase (EC 2.1.1.14)	Methionine biosynthesis
**MetR**	metE2	23	6	Methionine synthase II, vitamin-B12 independent (EC 2.1.1.14)	Methionine biosynthesis
**MetR**	metF	43	6	5,10-methylenetetrahydrofolate reductase (EC 1.5.1.20)	Methionine biosynthesis
**MetR**	glyA	43	6	Serine hydroxymethyltransferase (EC 2.1.2.1)	Methionine biosynthesis
**MetR**	PF08908	20	6	Protein of unknown function DUF1852	
**MetR**	metH	26	5	5-methyltetrahydrofolate--homocysteine methyltransferase (EC 2.1.1.13)	Methionine biosynthesis
**MetR**	luxS	21	2	S-ribosylhomocysteine lyase (EC 4.4.1.21) / Autoinducer-2 production protein LuxS	SAM recycling
**MetR**	metA	14	2	Homoserine O-succinyltransferase (EC 2.3.1.46)	Methionine biosynthesis
**MetR**	metE2-2	7	2	Methionine synthase II, vitamin-B12 independent (EC 2.1.1.14)	Methionine biosynthesis
**MetR**	PF03358	3	1	NADPH-dependent FMN reductase	
**MetR**	ilvI	15	1	Acetolactate synthase large subunit (EC 2.2.1.6)	Branched-chain amino acid biosynthesis
**MetR**	ilvH	15	1	Acetolactate synthase small subunit (EC 2.2.1.6)	Branched-chain amino acid biosynthesis
**MetR**	hmp	9	1	Flavohemoprotein (Hemoglobin-like protein) (Flavohemoglobin) (Nitric oxide dioxygenase) (EC 1.14.12.17)	Nitric oxide cell defense
**MetR**	metF-II	3	1	5,10-methylenetetrahydrofolate reductase, non-orthologous isozyme (EC 1.5.1.20)	Methionine biosynthesis
**MetR**	metC	7	1	Cystathionine beta-lyase (EC 4.4.1.8)	Methionine biosynthesis
**MetR**	HI0736	3	1	hypothetical sodium-dependent transporter	
**MetR**	PF01613	3	1	Flavin reductase-like, FMN-binding	
**MetR**	PF02677	1	1	Protein of unknown function DUF208	
**MetR**	pfl	3	1	Pyruvate formate-lyase (EC 2.3.1.54)	
**MetR**	dsbC	2	1	Thiol:disulfide interchange protein DsbC	
**MetR**	metQ2	3	1	Methionine ABC transporter substrate-binding protein	Methionine transport
**MetR**	thrC	2	1	Threonine synthase (EC 4.2.3.1)	Threonine biosynthesis
**MetR**	hom	2	1	Homoserine dehydrogenase (EC 1.1.1.3)	Methionine biosynthesis
**MetR**	metF2	2	1	5,10-methylenetetrahydrofolate reductase (EC 1.5.1.20)	Methionine biosynthesis
**MetR**	metH2	5	2	5-methyltetrahydrofolate--homocysteine methyltransferase (EC 2.1.1.13)	Methionine biosynthesis
**MetR**	metR2	1	1	Homocysteine-responsive transcriptional regulator of methionine metabolism, LysR family	Transcription regulation
**MetR**	gcvP	1	1	Glycine dehydrogenase [decarboxylating] (glycine cleavage system P protein) (EC 1.4.4.2)	Glycine cleavage system
**MetR**	gcvH	1	1	Glycine cleavage system H protein	Glycine cleavage system
**MetR**	mdeA	1	1	Methionine gamma-lyase (EC 4.4.1.11)	Methionine biosynthesis
**MetR**	bhmT	2	1	Betaine--homocysteine S-methyltransferase (EC 2.1.1.5)	Methionine biosynthesis
**NadR**	nadA	10	1	Quinolinate synthetase (EC 4.1.99.-)	NAD biosynthesis
**NadR**	pnuC	11	1	Ribosyl nicotinamide transporter	Niacin or Ribosyl nicotinamide transport
**NadR**	pncB	6	1	Nicotinate phosphoribosyltransferase (EC 2.4.2.11)	NAD metabolism
**NadR**	nadB	5	1	L-aspartate oxidase (EC 1.4.3.16)	NAD biosynthesis
**NadR**	nadR	2	1	Transcriptional regulator of NAD metabolism / Nicotinamide-nucleotide adenylyltransferase, NadR family (EC 2.7.7.1) / Ribosylnicotinamide kinase (EC 2.7.1.22)	NAD metabolism
**NadR**	niaP	1	1	Niacin transporter, MFS family	Niacin or Ribosyl nicotinamide transport
**NadQ**	nadA	30	7	Quinolinate synthetase (EC 4.1.99.-)	NAD biosynthesis
**NadQ**	nadC	28	7	Quinolinate phosphoribosyltransferase [decarboxylating] (EC 2.4.2.19)	NAD biosynthesis
**NadQ**	nadB	27	7	L-aspartate oxidase (EC 1.4.3.16)	NAD biosynthesis
**NadQ**	nadE	6	2	NAD synthetase (EC 6.3.1.5) / Glutamine amidotransferase chain of NAD synthetase	NAD biosynthesis
**NadQ**	nadQ	3	2	Transcriptional regulator of NAD metabolism, COG4111 family	Transcription regulation
**NadQ**	nadD	4	1	Nicotinate-nucleotide adenylyltransferase (EC 2.7.7.18) ## bacterial NadD family	NAD biosynthesis
**NadQ**	proA	4	1	Gamma-glutamyl phosphate reductase (EC 1.2.1.41)	Proline biosynthesis

**NadQ**	HNE_0691	1	1	hypothetical protein	
**NadQ**	nadC2	1	1	Quinolinate phosphoribosyltransferase [decarboxylating] (EC 2.4.2.19)	NAD biosynthesis
**NadQ**	nadA1	1	1	Quinolinate synthetase (EC 4.1.99.-)	NAD biosynthesis
**NadQ**	nadA2	1	1	Quinolinate synthetase (EC 4.1.99.-)	NAD biosynthesis
**NadQ**	nadB2	1	1	L-aspartate oxidase (EC 1.4.3.16)	NAD biosynthesis
**NadQ**	nadB1	1	1	L-aspartate oxidase (EC 1.4.3.16)	NAD biosynthesis
**NadQ**	nadC1	1	1	Quinolinate phosphoribosyltransferase [decarboxylating] (EC 2.4.2.19)	NAD biosynthesis
**NagC**	nagE	21	4	PTS system, N-acetylglucosamine-specific IIB component (EC 2.7.1.69) / PTS system, glucose-specific IIC component (EC 2.7.1.69)	N-acetylglucosamine utilization
**NagC**	nagA	31	4	N-acetylglucosamine-6-phosphate deacetylase (EC 3.5.1.25)	N-acetylglucosamine utilization
**NagC**	nagC	27	4	N-acetylglucosamine-6P-responsive transcriptional repressor NagC, ROK family	Transcription regulation
**NagC**	nagB	29	4	Glucosamine-6-phosphate deaminase (EC 3.5.99.6)	N-acetylglucosamine utilization
**NagC**	ptsI	15	3	Phosphoenolpyruvate-protein phosphotransferase of PTS system (EC 2.7.3.9)	Sugar transport
**NagC**	ptsH	15	3	Phosphocarrier protein of PTS system	Sugar transport
**NagC**	crr	14	3	PTS system, glucose-specific IIA component (EC 2.7.1.69)	Sugar transport
**NagC**	nagF	2	1	PTS system, glucose-specific IIA component (EC 2.7.1.69) / Phosphocarrier protein of PTS system / Phosphoenolpyruvate-protein phosphotransferase of PTS system (EC 2.7.3.9)	N-acetylglucosamine utilization
**NagC**	mcp	7	2	N-acetylglucosamine regulated methyl-accepting chemotaxis protein	Chemotaxis
**NagC**	omp	3	1	Outer membrane receptor protein	N-acetylglucosamine utilization
**NagC**	mcp2	5	2	N-acetylglucosamine regulated methyl-accepting chemotaxis protein	Chemotaxis
**NagC**	glmU	14	2	N-acetylglucosamine-1-phosphate uridyltransferase (EC 2.7.7.23) / Glucosamine-1-phosphate N-acetyltransferase (EC 2.3.1.157)	N-acetylglucosamine utilization
**NagC**	hex	11	2	Beta-hexosaminidase (EC 3.2.1.52)	Chitin degradation
**NagC**	rpmE1	2	1	50S ribosomal protein L31	
**NagC**	znuA	2	1	Zinc ABC transporter, periplasmic-binding protein ZnuA	
**NagC**	pyrG	10	1	CTP synthase (EC 6.3.4.2)	
**NagC**	eno	9	1	Enolase (EC 4.2.1.11)	Glycolysis
**NagC**	tfoX	9	1	DNA transformation protein TfoX	
**NagC**	tfoX1	9	1	DNA transformation protein TfoX1 (Sxy)	
**NagC**	ptsG	8	1	PTS system, glucose-specific IIB component (EC 2.7.1.69) / PTS system, glucose-specific IIC component (EC 2.7.1.69)	Mannose and glucose transport
**NagC**	fbaA	8	1	Fructose-bisphosphate aldolase class II (EC 4.1.2.13)	Glycolysis
**NagC**	pgk	8	1	Phosphoglycerate kinase (EC 2.7.2.3)	Glycolysis
**NagC**	chiA	8	1	Chitinase (EC 3.2.1.14)	Chitin degradation
**NagC**	manX	9	1	PTS system, mannose-specific IIAB component	Mannose and glucose transport
**NagC**	glmS	9	1	Glucosamine--fructose-6-phosphate aminotransferase [isomerizing] (EC 2.6.1.16)	N-acetylglucosamine utilization
**NagC**	manZ	9	1	PTS system, mannose-specific IID component	Mannose and glucose transport
**NagC**	manY	9	1	PTS system, mannose-specific IIc component	Mannose and glucose transport
**NagC**	nagE2	7	1	PTS system, N-acetylglucosamine-specific IIA component (EC 2.7.1.69) / PTS system, N-acetylglucosamine-specific IIB component (EC 2.7.1.69) / PTS system, N-acetylglucosamine-specific IIC component (EC 2.7.1.69)	N-acetylglucosamine utilization
**NagC**	aldE	7	1	Aldose 1-epimerase	
**NagC**	chi	6	2	Chitinase (EC 3.2.1.14)	Chitin degradation
**NagC**	nagD	8	1	Phosphatase NagD predicted to act in N-acetylglucosamine utilization subsystem	N-acetylglucosamine utilization
**NagC**	nagE1	6	1	PTS system, N-acetylglucosamine-specific IIB component (EC 2.7.1.69) / PTS system, N-acetylglucosamine-specific IIC component (EC 2.7.1.69)	N-acetylglucosamine utilization
**NagC**	gapA	5	1	NAD-dependent glyceraldehyde-3-phosphate dehydrogenase (EC 1.2.1.12)	Glycolysis
**NagC**	chbR	6	1	Chitobiose-specific regulator ChbR, AraC family	Transcription regulation
**NagC**	chbB	6	1	PTS system, chitobiose-specific IIB component (EC 2.7.1.69)	Chitobiose utilization
**NagC**	gdhA	1	1	NADP-specific glutamate dehydrogenase (EC 1.4.1.4)	Glutamate degradation
**NagC**	rpmJ1	1	1	ribosomal protein L36	

**NagC**	chbA	6	1	N,N'-diacetylchitobiose-specific PTS system, EIIA component	Chitobiose utilization
**NagC**	exbD	1	1	Biopolymer transport protein ExbD/TolR	
**NagC**	tonB	1	1	Periplasmic binding protein TonB	
**NagC**	chbC	6	1	PTS system, chitobiose-specific IIC component (EC 2.7.1.69)	Chitobiose utilization
**NagC**	exbB	1	1	Biopolymer transport protein ExbB	
**NagC**	chbG	6	1	Cellobiose phosphotransferase system YdjC-like protein	
**NagC**	chbF	5	1	Chitobiose-specific 6-phospho-beta-glucosidase ChbF (EC 3.2.1.86)	Chitobiose utilization
**NagC**	ybfM	4	1	N-acetylglucosamine-regulated outer membrane porin	Chitobiose utilization
**NagC**	cbp	4	1	Chitin binding protein	Chitin degradation
**NagC**	glgA	3	1	Glycogen synthase, ADP-glucose transglucosylase (EC 2.4.1.21)	Glycogen metabolism
**NagC**	grcA	3	1	autonomous glycyl radical cofactor GrcA	
**NagC**	chiP	3	1	Outer membrane chitoporin	Chitobiose transport
**NagC**	chiD	3	1	Chitodextrinase precursor (EC 3.2.1.14)	Chitin degradation
**NagC**	glgC	3	1	Glucose-1-phosphate adenylyltransferase (EC 2.7.7.27)	Glycogen metabolism
**NagC**	gapB	3	1	NADPH-dependent glyceraldehyde-3-phosphate dehydrogenase (EC 1.2.1.13)	Glycolysis
**NagC**	ompC	3	1	Predicted OmpC-like chitoporin	Chitobiose utilization
**NagC**	adh	2	1	Alcohol dehydrogenase (EC 1.1.1.1); Acetaldehyde dehydrogenase (EC 1.2.1.10)	
**NagC**	gltA	2	1	Citrate synthase (si) (EC 2.3.3.1)	Tricarboxylic acid cycle
**NagC**	chiS	2	1	Chitin catabolic cascade sensor histidine kinase ChiS	Transcription regulation
**NagC**	epd	2	1	D-erythrose-4-phosphate dehydrogenase (EC 1.2.1.72)	
**NagC**	galP	2	1	D-galactose transporter	Galactose transport
**NagC**	chi1	1	1	Chitinase (EC 3.2.1.14)	Chitin degradation
**NagC**	VP1029	1	1	Hypothetical protein	
**NagC**	ompU	1	1	Outer membrane protein OmpU	
**NagC**	VC1591	1	1	Oxidoreductase, short-chain dehydrogenase/reductase family	
**NagC**	aldC	1	1	Alpha-acetolactate decarboxylase (EC 4.1.1.5)	
**NagC**	budB	1	1	Acetolactate synthase, catabolic (EC 2.2.1.6)	
**NagC**	alsR	1	1	Transcriptional regulator of alpha-acetolactate operon alsR	Transcription regulation
**NagC**	nanM	1	1	N-acetylneuraminic acid-induced hypothetical transmembrane protein	N-acetylneuraminic acid transport
**NagC**	fimB	1	1	type 1 fimbriae regulatory protein FimB	
**NagC**	nanC	1	1	N-acetylneuraminic acid outer membrane channel protein NanC	N-acetylneuraminic acid transport
**NagQ**	nagQ	33	10	Predicted transcriptional regulator of N-Acetylglucosamine utilization, GntR family	Transcription regulation
**NagQ**	nagA	30	10	N-acetylglucosamine-6-phosphate deacetylase (EC 3.5.1.25)	N-acetylglucosamine utilization
**NagQ**	nagB2	26	9	Glucosamine-6-phosphate deaminase [isomerizing], alternative (EC 3.5.99.6)	N-acetylglucosamine utilization
**NagQ**	nagE	15	6	PTS system, N-acetylglucosamine-specific IIB component (EC 2.7.1.69) / PTS system, N-acetylglucosamine-specific IIC component (EC 2.7.1.69)	N-acetylglucosamine utilization
**NagQ**	ptsI	15	5	PTS system, glucose-specific IIA component (EC 2.7.1.69) / Phosphocarrier protein of PTS system / Phosphoenolpyruvate-protein phosphotransferase of PTS system (EC 2.7.3.9)	Sugar transport
**NagQ**	nagK	13	3	N-acetylglucosamine kinase of eukaryotic type (EC 2.7.1.59)	N-acetylglucosamine utilization
**NagQ**	murQ	8	3	N-acetylmuramic acid 6-phosphate etherase (EC 4.2.-.-)	N-acetylmuramic acid utilization
**NagQ**	nagT	11	3	N-Acetyl-D-glucosamine ABC transport system, sugar-binding protein	N-acetylglucosamine utilization
**NagQ**	nagV	10	3	N-Acetyl-D-glucosamine ABC transport system, permease protein 2	N-acetylglucosamine utilization
**NagQ**	nagU	10	3	N-Acetyl-D-glucosamine ABC transport system, permease protein 1	N-acetylglucosamine utilization
**NagQ**	nagW	10	3	N-Acetyl-D-glucosamine ABC transport system, ATP-binding component	N-acetylglucosamine utilization
**NagQ**	hex	3	2	Beta-hexosaminidase (EC 3.2.1.52)	N-acetylglucosamine utilization
**NagQ**	chiA	2	2	Chitinase (EC 3.2.1.14)	Chitin degradation
**NagQ**	cdxA	2	2	Chitodextrinase precursor (EC 3.2.1.14)	Chitin degradation
**NagQ**	omp_nag	4	1	N-acetylglucosamine-regulated TonB-dependent outer membrane receptor	Chitobiose utilization
**NagQ**	nagD	6	2	Hypothetical oxidoreductase related to N-acetylglucosamine utilization	
**NagQ**	nagZ	1	1	Beta N-acetyl-glucosaminidase (EC 3.2.1.52)	Chitin degradation

**NagQ**	nagB	3	1	Glucosamine-6-phosphate deaminase (EC 3.5.99.6)	N-acetylglucosamine utilization
**NagQ**	nagR	2	1	Transcriptional regulator of N-acetylglucosamine utilization, LacI family	Transcription regulation
**NagQ**	wecA	1	1	Undecaprenyl-phosphate N-acetylglucosaminyl 1-phosphate transferase (EC 2.7.8.-)	
**NagQ**	hex2	1	1	N-Acetyl-D-glucosamine ABC transport system ATP-binding protein	Chitin degradation
**NagQ**	cga	1	1	Glucoamylase (EC 3.2.1.3)	
**NagQ**	ybfM	1	1	N-acetylglucosamine-regulated outer membrane porin	Chitobiose utilization
**NagQ**	cbp21	1	1	Chitin binding protein	Chitin degradation
**NagQ**	chiC	1	1	Chitinase (EC 3.2.1.14)	Chitin degradation
**NagQ**	chi	1	1	Chitinase (EC 3.2.1.14)	Chitin degradation
**NagQ**	nagP	2	1	N-acetyl\glucosamine transporter, NagP	N-acetylmuramic acid utilization
**NagQ**	omp1	1	1	Outer membrane protein (porin)	Chitobiose utilization
**NagQ**	CC0542	1	1	Predicted periplasmic phosphohydrolase	
**NagQ**	MED297_05914	1	1	Hypothetical protein	
**NagQ**	nagX	1	1	N-acetylglucosamine related transporter, NagX	N-acetylmuramic acid utilization
**NagQ**	anaG	1	1	Alpha-N-acetylglucosaminidase (EC 3.2.1.50)	Alpha-N-acetylglucosaminidase
**NagQ**	mlr4776	1	1	Hypothetical protein	
**NagQ**	nagM	1	1	Predicted N-Acetylglucosamine ABC transporter, inner membrane subunit	N-acetylmuramic acid utilization
**NagQ**	nagO	1	1	Predicted N-Acetylglucosamine ABC transporter, periplasmic sugar-binding protein	N-acetylmuramic acid utilization
**NagQ**	nagN	1	1	Predicted N-Acetylglucosamine ABC transporter, permease protein	N-acetylmuramic acid utilization
**NagQ**	nagK2	1	1	N-acetylglucosamine kinase (EC 2.7.1.59), ROK family	N-acetylglucosamine utilization
**NagQ**	nagD1	1	1	Probable oxidoreductase	
**NagQ**	nagL	1	1	Predicted N-Acetylglucosamine ABC transporter, ATP-binding protein	N-acetylmuramic acid utilization
**NagR**	omp_nag	24	3	N-acetylglucosamine-regulated TonB-dependent outer membrane receptor	Chitobiose utilization
**NagR**	trpX	24	3	Tryptophan halogenase	
**NagR**	nagA	23	4	N-acetylglucosamine-6-phosphate deacetylase (EC 3.5.1.25)	N-acetylglucosamine utilization
**NagR**	nagP	23	4	PTS system, N-acetylglucosamine-specific IIB component (EC 2.7.1.69) / PTS system, N-acetylglucosamine-specific IIC component (EC 2.7.1.69)	N-acetylglucosamine utilization
**NagR**	nagK	21	3	N-acetylglucosamine kinase of eukaryotic type (EC 2.7.1.59)	N-acetylglucosamine utilization
**NagR**	nagX	19	3	N-acetylglucosamine related transporter, NagX	N-acetylglucosamine utilization
**NagR**	hex	18	4	Beta-hexosaminidase (EC 3.2.1.52)	Chitobiose utilization
**NagR**	nagB2	14	4	Glucosamine-6-phosphate deaminase [isomerizing], alternative (EC 3.5.99.6)	N-acetylglucosamine utilization
**NagR**	nagK2	6	1	Predicted N-acetylglucosamine kinase, glucokinase-like (EC 2.7.1.59)	N-acetylglucosamine utilization
**NagR**	nagR	8	3	Transcriptional regulator of N-acetylglucosamine utilization, LacI family	Transcription regulation
**NagR**	nagB	9	3	Glucosamine-6-phosphate deaminase (EC 3.5.99.6)	N-acetylglucosamine utilization
**NagR**	chiA	10	3	Chitinase (EC 3.2.1.14)	Chitin degradation
**NagR**	nixC	3	1	N-acetylglucosamine-regulated TonB-dependent outer membrane receptor	Chitobiose utilization
**NagR**	naxA	3	1	N-acetylglucosamine-regulated TonB-dependent outer membrane receptor	Chitobiose utilization
**NagR**	cbp	7	2	Chitn and N-acetylglucosamine-binding protein A	Chitin degradation
**NagR**	pckA	2	1	Phosphoenolpyruvate carboxykinase [ATP] (EC 4.1.1.49)	Gluconeogenesis
**NagR**	chiD	2	1	Chitodextrinase (EC 3.2.1.14)	Chitin degradation
**NagR**	pgi2	2	1	Glucose-6-phosphate isomerase (EC 5.3.1.9)	Glycolysis
**NagR**	sapC	10	1	Peptide transport system permease protein sapC (TC 3.A.1.5.5)	
**NagR**	mcp_nag	8	1	N-acetylglucosamine regulated methyl-accepting chemotaxis protein	Chemotaxis
**NagR**	nixD	2	1	N-acetylglucosamine-regulated TonB-dependent outer membrane receptor	Chitobiose utilization
**NagR**	nixB	2	1	N-acetylglucosamine-regulated TonB-dependent outer membrane receptor	Chitobiose utilization
**NagR**	pilM	7	1	homolog of type IV pilus assembly protein PilM	
**NagR**	duf81	6	1	protein of unknown function DUF81	
**NagR**	nagS2	1	1	Putative sulfatase (EC 3.1.6.-)	
**NagR**	SO0851	5	1	prepilin-type cleavage/methylation-like protein	

**NagR**	CPS_2383	1	1	Putative surface protein	
**NagR**	SO0852	5	1	Type IV fimbrial biogenesis protein PilV	Fimbriae biogenesis
**NagR**	ATW7_01305	1	1	Pass1-related protein	
**NagR**	bglX	1	1	Beta-glucosidase (EC 3.2.1.21)	Glucosides utilization
**NagR**	SO0850	5	1	Type IV fimbrial biogenesis protein PilX	Fimbriae biogenesis
**NagR**	SO0854	4	1	Type IV pilus biogenesis protein PilE	Fimbriae biogenesis
**NagR**	SO0853	4	1	Type IV fimbrial biogenesis protein FimT	Fimbriae biogenesis
**NagR**	nixA	1	1	N-acetylglucosamine-regulated TonB-dependent outer membrane receptor	Chitobiose utilization
**NagR**	cbp2	2	1	putative chitn-binding protein, exported	Chitin degradation
**NagR**	cdxA	2	1	Chitodextrinase precursor (EC 3.2.1.14)	Chitin degradation
**NagR**	pdaA	1	1	Peptidoglycan N-acetylglucosamine deacetylase	N-acetylglucosamine utilization
**NagR**	omp_nag2	1	1	N-acetylglucosamine-regulated TonB-dependent outer membrane receptor	Chitobiose utilization
**NagR**	chiA3	1	1	chitodextrinase	Chitin degradation
**NrdR**	nrdA	126	19	Ribonucleotide reductase of class Ia (aerobic), alpha subunit (EC 1.17.4.1)	Deoxyribonucleotide biosynthesis
**NrdR**	nrdB	117	19	Ribonucleotide reductase of class Ia (aerobic), beta subunit (EC 1.17.4.1)	Deoxyribonucleotide biosynthesis
**NrdR**	nrdD	70	12	Ribonucleotide reductase of class III (anaerobic), large subunit (EC 1.17.4.2)	Deoxyribonucleotide biosynthesis
**NrdR**	nrdG	63	12	Ribonucleotide reductase of class III (anaerobic), activating protein (EC 1.97.1.4)	Deoxyribonucleotide biosynthesis
**NrdR**	nrdJ	46	5	Ribonucleotide reductase of class II (coenzyme B12-dependent) (EC 1.17.4.1) @ intein-containing	Deoxyribonucleotide biosynthesis
**NrdR**	yfaE	40	7	Ferredoxin	Oxidoreductase
**NrdR**	nrdJa	21	4	Ribonucleotide reductase of class II (coenzyme B12-dependent) (EC 1.17.4.1)	Deoxyribonucleotide biosynthesis
**NrdR**	nrdH	16	2	Glutaredoxin-like protein NrdH, required for reduction of Ribonucleotide reductase class Ib	Deoxyribonucleotide biosynthesis
**NrdR**	nrdF	15	2	Ribonucleotide reductase of class Ib (aerobic), beta subunit (EC 1.17.4.1)	Deoxyribonucleotide biosynthesis
**NrdR**	nrdI	15	2	Ribonucleotide reductase stimulatory protein NrdI	Deoxyribonucleotide biosynthesis
**NrdR**	nrdE	15	2	Ribonucleotide reductase of class Ib (aerobic), alpha subunit (EC 1.17.4.1)	Deoxyribonucleotide biosynthesis
**NrdR**	SO2417	16	1	Ferredoxin	Oxidoreductase
**NrdR**	trxA	4	1	Thioredoxin	Oxidoreductase
**NrdR**	topA	8	1	DNA topoisomerase I (EC 5.99.1.2)	Replication
**NrdR**	nrdJb	5	3	Ribonucleotide reductase of class II (coenzyme B12-dependent), alpha subunit (EC 1.17.4.1)	Deoxyribonucleotide biosynthesis
**NrdR**	nrdA2	2	2	Ribonucleotide reductase of class Ia (aerobic), alpha subunit (EC 1.17.4.1)	Deoxyribonucleotide biosynthesis
**NrdR**	nrdB2	2	2	Ribonucleotide reductase of class Ia (aerobic), beta subunit (EC 1.17.4.1)	Deoxyribonucleotide biosynthesis
**NrdR**	Sala_1774	2	1	protein of unknown function DUF559	
**NrdR**	Ajs_0086	3	1	hypothetical protein	
**NrdR**	nrdA1	1	1	Ribonucleotide reductase of class Ia (aerobic), alpha subunit (EC 1.17.4.1)	Deoxyribonucleotide biosynthesis
**NrdR**	nrdB1	1	1	Ribonucleotide reductase of class Ia (aerobic), beta subunit (EC 1.17.4.1)	Deoxyribonucleotide biosynthesis
**NrdR**	RSp0964	1	1	hypothetical protein	
**NrdR**	nrdJ1	1	1	Ribonucleotide reductase of class II (coenzyme B12-dependent) (EC 1.17.4.1)	Deoxyribonucleotide biosynthesis
**NrdR**	nrdD fragment	1	1	Ribonucleotide reductase of class III (anaerobic), large subunit (EC 1.17.4.2)	Deoxyribonucleotide biosynthesis
**NrtR**	nrtR	24	10	Nudix-related transcriptional regulator NrtR	Transcription regulation
**NrtR**	nrtX	7	6	NrtR-regulated hypothetical OrfX, Band 7 protein domain	Putative NAD metabolism genes
**NrtR**	pncB	15	5	Nicotinate phosphoribosyltransferase (EC 2.4.2.11)	NAD metabolism
**NrtR**	nrtY	6	5	NrtR-regulated hypothetical OrfY, PpnK-type ATP-NAD kinase domain	Putative NAD metabolism genes
**NrtR**	pncA	13	5	Nicotinamidase (EC 3.5.1.19)	NAD metabolism
**NrtR**	nadV	7	4	Nicotinamide phosphoribosyltransferase (EC 2.4.2.12)	NAD metabolism
**NrtR**	prs	6	3	Ribose-phosphate pyrophosphokinase (EC 2.7.6.1)	NAD metabolism
**NrtR**	nadE	8	4	NAD synthetase (EC 6.3.1.5)	NAD metabolism
**NrtR**	nadD	4	3	Nicotinate-nucleotide adenylyltransferase (EC 2.7.7.18)	NAD metabolism
**NrtR**	nadM	4	2	Nicotinamide-nucleotide adenylyltransferase, NadM family (EC 2.7.7.1) / ADP-ribose pyrophosphatase (EC 3.6.1.13)	NAD metabolism

**NrtR**	pnuC	1	1	Ribosyl nicotinamide transporter, PnuC-like	NAD metabolism
**NrtR**	nadR	1	1	Nicotinamide-nucleotide adenylyltransferase, NadR family (EC 2.7.7.1) / Ribosylnicotinamide kinase (EC 2.7.1.22)	NAD metabolism
**NtrC**	glnA	163	19	Glutamine synthetase type I (EC 6.3.1.2)	Glutamine biosynthesis
**NtrC**	ntrC	157	19	nitrogen regulation protein NR(I)	Transcription regulation
**NtrC**	ntrB	157	19	Nitrogen regulation protein NR(II) (EC 2.7.3.-)	Nitrogen metabolism regulation proteins
**NtrC**	amtB	134	17	ammonium transporter	Nitrogen source transport
**NtrC**	glnK	107	13	nitrogen regulatory protein P-II	Nitrogen metabolism regulation proteins
**NtrC**	glnB	66	7	Nitrogen regulatory protein P-II	Nitrogen metabolism regulation proteins
**NtrC**	nifR3	41	5	Nitrogen assimilation transcriptional regulator NtrX, Fis family	
**NtrC**	nasD	34	8	Nitrite reductase, large subunit (EC 1.7.1.4)	Nitrogen metabolism
**NtrC**	nasE	33	8	Nitrite reductase, small subunit (EC 1.7.1.4)	Nitrogen metabolism
**NtrC**	ntrY	30	4	Nitrogen regulation protein NtrY, sensor kinase (EC 2.7.3.-)	Nitrogen metabolism
**NtrC**	amtB2	30	8	ammonium transporter	Nitrogen source transport
**NtrC**	narK	23	6	Nitrate/nitrite antiporter	Nitrogen source transport
**NtrC**	ntrX	24	3	Nitrogen assimilation transcriptional regulator NtrX, Fis family	Nitrogen metabolism
**NtrC**	ntrZ	23	3	Conserved hypothetical signal peptide protein	
**NtrC**	nasA	23	7	Assimilatory nitrate reductase, large subunit (EC:1.7.99.4)	Nitrogen metabolism
**NtrC**	nrtC	22	4	Nitrate ABC transporter, ATP-binding component	Nitrogen source transport
**NtrC**	nrtB	21	4	Nitrate ABC transporter, permease component	Nitrogen source transport
**NtrC**	nrtA	21	4	Nitrate ABC transporter, substrate-binding component	Nitrogen source transport
**NtrC**	urtB	22	3	Urea ABC transporter, permease component 2	Nitrogen source transport
**NtrC**	gdhA	13	3	NADP-specific glutamate dehydrogenase (EC 1.4.1.4)	Nitrogen metabolism
**NtrC**	ureD	20	3	Urease accessory protein, UreD	Nitrogen metabolism
**NtrC**	ureA	20	3	Urease, gamma subunit (EC 3.5.1.5)	Nitrogen metabolism
**NtrC**	ureB	19	3	Urease, beta subunit (EC 3.5.1.5)	Nitrogen metabolism
**NtrC**	ureC	19	3	Urease, alpha subunit (EC 3.5.1.5)	Nitrogen metabolism
**NtrC**	urtA	18	3	Urea ABC transporter, substrate-binding component	Nitrogen source transport
**NtrC**	urtD	16	3	Urea ABC transporter, ATP-binding component 1	Nitrogen source transport
**NtrC**	CHP02001	12	2	Conserved hypothetical protein CHP02001	
**NtrC**	dppC	10	2	Dipeptide ABC transporter, permease component 2	Nitrogen source transport
**NtrC**	dppA	10	2	Dipeptide ABC transporter, substrate-binding component	Nitrogen source transport
**NtrC**	dppB	10	2	Dipeptide ABC transporter, permease component 1	Nitrogen source transport
**NtrC**	ureE	15	2	Urease accessory protein, UreE	Nitrogen metabolism
**NtrC**	ureG	15	2	Urease accessory protein, UreG	Nitrogen metabolism
**NtrC**	ureF	15	2	Urease accessory protein, UreF	Nitrogen metabolism
**NtrC**	cysG	10	4	Uroporphyrinogen-III methyltransferase (EC 2.1.1.107)	Porphyrin biosynthesis
**NtrC**	urtE	15	3	Urea ABC transporter, ATP-binding component 1	Nitrogen source transport
**NtrC**	glnK2	12	3	nitrogen regulatory protein P-II	Nitrogen metabolism
**NtrC**	nasBA	8	1	Assimilatory nitrate reductase, large and small subunits protein fusion (EC:1.7.99.4)	Nitrogen metabolism
**NtrC**	dat	4	1	D-alanine aminotransferase (EC 2.6.1.21)	Proline degradation
**NtrC**	nasB	8	2	Assimilatory nitrate reductase, small subunit (EC:1.7.99.4)	Nitrogen metabolism
**NtrC**	gltJ	11	1	Glutamate-aspartate ABC transporter, transmembrane component 1	Nitrogen source transport
**NtrC**	gltK	11	1	Glutamate-aspartate ABC transporter, transmembrane component 2	Nitrogen source transport
**NtrC**	gltL	11	1	Glutamate-aspartate ABC transporter, ATP-binding component	Nitrogen source transport
**NtrC**	gltI	11	1	Glutamate-aspartate ABC transporter, substrate-binding component	Nitrogen source transport
**NtrC**	dppD	5	1	Dipeptide ABC transporter, ATP-binding component 1	Nitrogen source transport
**NtrC**	dppF	5	1	Dipeptide ABC transporter, ATP-binding component 2	Nitrogen source transport
**NtrC**	ntrXY	5	1	Nitrogen assimilation transcriptional regulator NtrX, Fis family	Transcription regulation
**NtrC**	nifR	7	1	Predicted oxidoreductase, FAD binding	

**NtrC**	urtC	10	2	Urea ABC transporter, permease component 2	Nitrogen source transport
**NtrC**	ybdK	6	1	Carboxylate-amine ligase	
**NtrC**	PF04168	6	1	Conserved hypothetical protein	
**NtrC**	gltB	4	2	Glutamate synthase, large chain (EC 1.4.1.13)	Glutamate biosynthesis
**NtrC**	gltD	4	2	Glutamate synthase, small chain (EC 1.4.1.13)	Glutamate biosynthesis
**NtrC**	nrtA2	8	2	Nitrate ABC transporter, substrate-binding component	Nitrogen source transport
**NtrC**	nifEN	6	1	Nitrogenase FeMo-cofactor scaffold and assembly protein	Nitrogen metabolism
**NtrC**	Gmet_0693	6	1	Conserved hypothetical protein	
**NtrC**	PF01841	5	1	Transglutaminase-like protein	
**NtrC**	rutG	5	2	Uracil permease	Nitrogen source transport
**NtrC**	hisM	7	1	Histidine ABC transporter, transmembrane component 2	Nitrogen source transport
**NtrC**	rutC	5	2	Aminoacrylate peracid reductase	Pyrimidine Degradation
**NtrC**	rutA	5	2	Pyrimidine oxygenase	Pyrimidine Degradation
**NtrC**	glnP	7	1	Glutamine ABC transporter, transmembrane component	Nitrogen source transport
**NtrC**	hisP	7	1	Histidine ABC transporter, ATP-binding component	Nitrogen source transport
**NtrC**	glnH	7	1	Glutamine ABC transporter, substrate-binding component	Nitrogen source transport
**NtrC**	hisQ	7	1	Histidine ABC transporter, transmembrane component 1	Nitrogen source transport
**NtrC**	rutB	5	2	Peroxyureidoacrylate / ureidoacrylate amido hydrolase	
**NtrC**	rutF	5	2	Flavin reductase	Pyrimidine Degradation
**NtrC**	hisJ	7	1	Histidine ABC transporter, substrate-binding component	Nitrogen source transport
**NtrC**	astD	6	1	Succinylglutamic semialdehyde dehydrogenase (EC 1.2.1.71)	Arginine degradation
**NtrC**	glnQ	6	1	Glutamine ABC transporter, ATP-binding component	Nitrogen source transport
**NtrC**	astB	6	1	Succinylarginine dihydrolase (EC 3.5.3.23)	Arginine degradation
**NtrC**	astA	6	1	Arginine N-succinyltransferase (EC 2.3.1.109)	Arginine degradation
**NtrC**	nac	6	1	Nitrogen assimilation transcriptional regulator, LysR family	Transcription regulation
**NtrC**	astC	6	1	Succinylornithine transaminase (EC 2.6.1.81)	Arginine degradation
**NtrC**	astE	6	1	Succinylglutamate desuccinylase (EC 3.5.1.96)	Arginine degradation
**NtrC**	uctA	7	1	Urea carboxylase-related ABC transporter, substrate-binding component	Nitrogen source transport
**NtrC**	COG0733	2	1	Predicted sodium dependent transporter	
**NtrC**	glnK1	4	1	Nitrogen regulatory protein P-II	Nitrogen metabolism
**NtrC**	uctB	6	1	Urea carboxylase-related ABC transporter, permease component	Nitrogen source transport
**NtrC**	nasT	6	1	Nitrogen assimilation attenuator protein NasT	Transcription regulation
**NtrC**	uctC	6	1	Urea carboxylase-related ABC transporter, ATP-binding component	Nitrogen source transport
**NtrC**	ureJ	5	1	Urease accessory protein, UreJ	Nitrogen metabolism
**NtrC**	alsT	3	1	Predicted alanin/sodium symporter	Nitrogen source transport
**NtrC**	ansA	3	1	L-asparaginase I (EC 3.5.1.1)	Asparagine degradation
**NtrC**	dppDE	5	1	Dipeptide ABC transporter, ATP-binding component	Nitrogen source transport
**NtrC**	hmp	5	2	Nitric oxide dioxygenase	Nitrogen stress response
**NtrC**	uahA	5	1	Urea carboxylase (EC 6.3.4.6)	Nitrogen metabolism
**NtrC**	uahB	5	1	Urea carboxylase-related aminomethyltransferase (EC 2.1.2.10)	Nitrogen metabolism
**NtrC**	uahC	5	1	Urea carboxylase-related aminomethyltransferase (EC 2.1.2.10)	Nitrogen metabolism
**NtrC**	EAM_0873	4	1	Predicted ABC transporter, permease component 1	
**NtrC**	EAM_0875	4	1	Predicted ABC transporter, ATP-binding component	
**NtrC**	potG	4	1	Putrescine ABC transporter, ATP-binding component	Nitrogen source transport
**NtrC**	potI	4	1	Putrescine ABC transporter, transmembrane component 1	Nitrogen source transport
**NtrC**	rutD	4	1	Aminoacrylate hydrolase	Pyrimidine Degradation
**NtrC**	PF09694	4	1	Conserved hypothetical protein, nitrogen assimilation associated	
**NtrC**	ygjG	4	1	Putrescine aminotransferase (EC 2.6.1.82)	Putrescine metabolism
**NtrC**	EAM_0872	4	1	Predicted ABC transporter, substrate-binding component	
**NtrC**	EAM_0874	4	1	Predicted ABC transporter, permease component 2	

**NtrC**	potH	4	1	Putrescine ABC transporter, transmembrane component 2	Nitrogen source transport
**NtrC**	nasB2	4	1	Assimilatory nitrate reductase, large subunit (EC:1.7.99.4)	Nitrogen metabolism
**NtrC**	nirA	4	1	Ferredoxin--nitrite reductase (EC 1.7.7.1)	Nitrogen metabolism
**NtrC**	ddpC	3	1	Dipeptide ABC transporter, transmembrane component 2 (TC 3.A.1.5.2)	Nitrogen source transport
**NtrC**	amaB	3	1	N-carbamoyl-L-amino acid hydrolase	
**NtrC**	potC	2	1	Putrescine ABC transporter, transmembrane component 2 (TC_3.A.1.11.1)	Nitrogen source transport
**NtrC**	pucG	3	1	Serine--pyruvate aminotransferase (EC 2.6.1.51) / L-alanine:glyoxylate aminotransferase (EC 2.6.1.44)	Amino acid degradation
**NtrC**	rutE	3	1	3-hydroxy propionic acid dehydrogenase	Pyrimidine Degradation
**NtrC**	ddpA	3	1	Dipeptide ABC transporter, substrate-binding component (TC 3.A.1.5.2)	Nitrogen source transport
**NtrC**	potA	2	1	Putrescine ABC transporter, ATP-binding component (TC_3.A.1.11.1)	Nitrogen source transport
**NtrC**	ddpX	3	1	D-alanyl-D-alanine dipeptidase (EC 3.4.13.-)	
**NtrC**	ddpB	3	1	Dipeptide ABC transporter, transmembrane component 1 (TC 3.A.1.5.2)	Nitrogen source transport
**NtrC**	rutE2	1	1	3-hydroxy propionic acid dehydrogenase	Pyrimidine Degradation
**NtrC**	potB	2	1	Putrescine ABC transporter, transmembrane component 1 (TC_3.A.1.11.1)	Nitrogen source transport
**NtrC**	rutR	1	1	Pyrimidine catabolism transcriptional regulator RutR, TetR family	Transcription regulation
**NtrC**	potD	2	1	Putrescine ABC transporter, substrate-binding component (TC_3.A.1.11.1)	Nitrogen source transport
**NtrC**	ddpD	3	1	Dipeptide ABC transporter, transmembrane component 3 (TC 3.A.1.5.2)	Nitrogen source transport
**NtrC**	ddpF	3	1	Dipeptide ABC transporter, ABC-binding component (TC 3.A.1.5.2)	Nitrogen source transport
**NtrC**	ybiB	2	1	Conserved hypothetical protein	
**NtrC**	CKO_01526	2	1	Predicted transcriptional regulator, RpiR family	Transcription regulation
**NtrC**	TM1040_0383	2	1	Conserved hypothetical protein	
**NtrC**	COG0547	2	1	Glycosyl transferase, family 3	
**NtrC**	atzF	2	1	Allophanate hydrolase (EC 3.5.1.54)	Nitrogen metabolism
**NtrC**	atzF2	2	1	Allophanate hydrolase (EC 3.5.1.54)	Nitrogen metabolism
**NtrC**	Jann_1753	2	1	Conserved hypothetical protein	
**NtrC**	ISBma2	1	1	Transposase, IS4	
**NtrC**	nrtB3	1	1	Nitrate ABC transporter, permease component	Nitrogen source transport
**NtrC**	nrtC3	1	1	Nitrate ABC transporter, ATP-binding component	Nitrogen source transport
**NtrC**	nrtA3	1	1	Nitrate ABC transporter, substrate-binding component	Nitrogen source transport
**NtrC**	Daro_0818	1	1	Hypothetical protein	
**NtrC**	pkn	1	1	Probable serine/threonine-protein kinase SCO3848	
**NtrC**	PF02627	1	1	Putative alkylhydroperoxidase	
**NtrC**	OB2597_07045	1	1	Conserved hypothetical protein	
**NtrC**	OB2597_07055	1	1	Hypothetical protein	
**NtrC**	Xaut_1081	1	1	Hypothetical protein	
**NtrC**	NGR_b03860	1	1	Hypothetical protein	
**NtrC**	speB	1	1	Agmatinase (EC 3.5.3.11)	Putrescine metabolism
**NtrC**	Jann_1751	1	1	Hypothetical protein	
**PdhR**	aceE	55	6	Pyruvate dehydrogenase E1 component (EC 1.2.4.1)	Pyruvate metabolism
**PdhR**	pdhR	55	6	Transcriptional repressor for pyruvate dehydrogenase complex	Transcription regulation
**PdhR**	aceF	54	6	Dihydrolipoamide acetyltransferase component of pyruvate dehydrogenase complex (EC 2.3.1.12)	Pyruvate metabolism
**PdhR**	lpdA	49	5	Dihydrolipoamide dehydrogenase of pyruvate dehydrogenase complex (EC 1.8.1.4)	Pyruvate metabolism
**PdhR**	oadB	19	2	Oxaloacetate decarboxylase beta chain (EC 4.1.1.3)	Pyruvate metabolism
**PdhR**	oadA	19	2	Oxaloacetate decarboxylase alpha chain (EC 4.1.1.3)	Pyruvate metabolism
**PdhR**	oadG	18	2	Oxaloacetate decarboxylase gamma chain (EC 4.1.1.3)	Pyruvate metabolism
**PdhR**	pflA	16	1	Pyruvate formate-lyase activating enzyme (EC 1.97.1.4)	Formate metabolism
**PdhR**	pflB	16	1	Pyruvate formate-lyase (EC 2.3.1.54)	Formate metabolism
**PdhR**	aceB	14	1	Malate synthase (EC 2.3.3.9)	Tricarboxylic acid cycle

**PdhR**	aceA	12	1	Isocitrate lyase (EC 4.1.3.1)	Tricarboxylic acid cycle
**PdhR**	pflX	11	1	pyruvate formate lyase-related hypothetical transporter	
**PdhR**	ndh	8	1	NADH dehydrogenase	NAD metabolism
**PdhR**	hemL	6	1	Glutamate-1-semialdehyde aminotransferase (EC 5.4.3.8)	Porphyrin biosynthesis
**PdhR**	focA	6	1	formate transporter	
**PdhR**	yfiD	4	1	stress-induced alternate pyruvate formate-lyase subunit	
**PdhR**	deaD	5	1	Cold-shock DEAD-box protein A	
**PdhR**	cyoC	3	2	Cytochrome O ubiquinol oxidase subunit III (EC 1.10.3.-)	Electron transfer chain
**PdhR**	cyoE	3	2	Heme O synthase, protoheme IX farnesyltransferase (EC 2.5.1.-) COX10-CtaB	Porphyrin biosynthesis
**PdhR**	cyoD	3	2	Cytochrome O ubiquinol oxidase subunit IV (EC 1.10.3.-)	Electron transfer chain
**PdhR**	cyoB	3	2	Cytochrome O ubiquinol oxidase subunit I (EC 1.10.3.-)	Electron transfer chain
**PdhR**	cyoA	3	2	Cytochrome O ubiquinol oxidase subunit II (EC 1.10.3.-)	Electron transfer chain
**PdhR**	dld	2	1	Predicted D-lactate dehydrogenase, Fe-S protein, FAD/FMN-containing	Lactate metabolism
**PdhR**	lldP	2	1	L-lactate permease	Lactate metabolism
**PdhR**	grcA	1	1	stress-induced alternate pyruvate formate-lyase subunit	Pyruvate metabolism
**PdhR**	glcB	1	1	Malate synthase G (EC 2.3.3.9)	Glycolate utilization
**PdhR**	glcD	1	1	Glycolate dehydrogenase (EC 1.1.99.14), subunit GlcD	Glycolate utilization
**PdhR**	glcG	1	1	Hypothetical protein GlcG in glycolate utilization operon	Glycolate utilization
**PdhR**	glcE	1	1	Glycolate dehydrogenase (EC 1.1.99.14), FAD-binding subunit GlcE	Glycolate utilization
**PdhR**	glcF	1	1	Glycolate dehydrogenase (EC 1.1.99.14), iron-sulfur subunit GlcF	Glycolate utilization
**PdhR**	SO0273	1	1	protein of unknown function DUF1439	
**PdhR**	sdhC	1	1	succinate dehydrogenase, cytochrome b556 subunit	Tricarboxylic acid cycle
**PdhR**	gltA	1	1	citrate synthase	Tricarboxylic acid cycle
**PdhR**	ppc	1	1	Phosphoenolpyruvate carboxylase (EC 4.1.1.31)	Pyruvate metabolism
**PdhR**	sdhA	1	1	succinate dehydrogenase, flavoprotein subunit	Tricarboxylic acid cycle
**PdhR**	sdhD	1	1	succinate dehydrogenase, hydrophobic membrane anchor protein	Tricarboxylic acid cycle
**PdhR**	sdhB	1	1	succinate dehydrogenase, iron-sulfur protein	Tricarboxylic acid cycle
**PsrA**	psrA	69	12	Predicted transcriptional regulator for fatty acid degradation PsrA, TetR family	Transcription regulation
**PsrA**	fadA	56	10	3-ketoacyl-CoA thiolase (EC 2.3.1.16)	Fatty acid degradation
**PsrA**	fadB	55	9	Enoyl-CoA hydratase (EC 4.2.1.17)	Fatty acid degradation
**PsrA**	fadD	25	5	Long-chain-fatty-acid--CoA ligase (EC 6.2.1.3)	Fatty acid degradation
**PsrA**	acdH	26	4	Acyl-CoA dehydrogenase (EC 1.3.99.3)	Fatty acid degradation
**PsrA**	fadH	31	6	2,4-dienoyl-CoA reductase [NADPH] (EC 1.3.1.34)	Fatty acid degradation
**PsrA**	etfD	31	6	Electron transfer flavoprotein-ubiquinone oxidoreductase (EC 1.5.5.1)	Electron transfer chain for fatty acid degradation
**PsrA**	fadJ	27	3	Enoyl-CoA hydratase (EC 4.2.1.17) / Delta(3)-cis-delta(2)-trans-enoyl-CoA isomerase (EC 5.3.3.8) / 3-hydroxyacyl-CoA dehydrogenase (EC 1.1.1.35) / 3-hydroxybutyryl-CoA epimerase (EC 5.1.2.3)	Fatty acid degradation
**PsrA**	fadI	27	3	3-ketoacyl-CoA thiolase (EC 2.3.1.16) @ Acetyl-CoA acetyltransferase (EC 2.3.1.9)	Fatty acid degradation
**PsrA**	etfA	27	5	electron transfer flavoprotein, alpha subunit	Electron transfer chain for fatty acid degradation
**PsrA**	etfB	27	5	electron transfer flavoprotein, beta subunit	Electron transfer chain for fatty acid degradation
**PsrA**	fadE	17	5	Acyl-CoA dehydrogenase, short-chain specific (EC 1.3.99.2)	Fatty acid degradation
**PsrA**	fadE1	21	3	Acyl-CoA dehydrogenase, short-chain specific (EC 1.3.99.2)	Fatty acid degradation
**PsrA**	fabG	16	2	3-oxoacyl-[acyl-carrier protein] reductase (EC 1.1.1.100)	Fatty acid biosynthesis
**PsrA**	fabF	14	3	3-oxoacyl-[acyl-carrier-protein] synthase, KASII (EC 2.3.1.41)	Fatty acid biosynthesis
**PsrA**	fadD2	18	4	Long-chain-fatty-acid--CoA ligase (EC 6.2.1.3)	Fatty acid degradation
**PsrA**	echH	20	3	Enoyl-CoA hydratase [valine degradation] (EC 4.2.1.17)	Fatty acid degradation
**PsrA**	fadL	9	3	Long-chain fatty acid transport protein	Fatty acid degradation
**PsrA**	scp	13	3	Sterol-binding domain protein	
**PsrA**	fabH	12	2	3-oxoacyl-[acyl-carrier-protein] synthase, KASIII (EC 2.3.1.41)	Fatty acid biosynthesis
**PsrA**	fabD	12	2	Malonyl CoA-acyl carrier protein transacylase (EC 2.3.1.39)	Fatty acid biosynthesis

**PsrA**	acpP	12	2	Acyl carrier protein	
**PsrA**	acdH1	12	2	Acyl-CoA dehydrogenase (EC 1.3.99.3)	Fatty acid degradation
**PsrA**	fadE2	18	2	Acyl-CoA dehydrogenase, short-chain specific (EC 1.3.99.2)	Fatty acid degradation
**PsrA**	fadD1	17	2	Long-chain-fatty-acid--CoA ligase (EC 6.2.1.3)	Fatty acid degradation
**PsrA**	sdhC	16	1	succinate dehydrogenase, cytochrome b556 subunit	Tricarboxylic acid cycle
**PsrA**	gltA	16	1	citrate synthase	Tricarboxylic acid cycle
**PsrA**	aroQ	3	1	3-dehydroquinate dehydratase II (EC 4.2.1.10)	Aromatic amino acid biosynthesis
**PsrA**	SO2935	16	1	oxidoreductase, short-chain dehydrogenase/reductase family	
**PsrA**	sdhA	16	1	succinate dehydrogenase, flavoprotein subunit	Tricarboxylic acid cycle
**PsrA**	rpoS	8	1	RNA polymerase sigma factor RpoS	Transcription
**PsrA**	aceB	16	1	malate synthase A	Tricarboxylic acid cycle
**PsrA**	sdhD	16	1	succinate dehydrogenase subunit D	Tricarboxylic acid cycle
**PsrA**	acdB	5	1	Enoyl-CoA hydratase (EC 4.2.1.17) / 3,2-trans-enoyl-CoA isomerase (EC 5.3.3.8) / 3-hydroxyacyl-CoA dehydrogenase (EC 1.1.1.35)	Fatty acid degradation
**PsrA**	sdhB	16	1	succinate dehydrogenase, iron-sulfur protein	Tricarboxylic acid cycle
**PsrA**	bccP	3	1	Biotin carboxyl carrier protein of acetyl-CoA carboxylase	
**PsrA**	accC	3	1	Biotin carboxylase of acetyl-CoA carboxylase (EC 6.3.4.14)	
**PsrA**	acdA	5	1	3-ketoacyl-CoA thiolase (EC 2.3.1.16) @ Acetyl-CoA acetyltransferase (EC 2.3.1.9)	Fatty acid degradation
**PsrA**	acdH2	5	4	Acyl-CoA dehydrogenase (EC 1.3.99.3)	Fatty acid degradation
**PsrA**	SO0881	14	1	conserved hypothetical protein	
**PsrA**	SO0882	14	1	oxidoreductase, GMC family	
**PsrA**	paaI	3	1	Phenylacetic acid degradation protein paaI	
**PsrA**	algQ	6	1	Regulator of RNA polymerase sigma(70) subunit, Rsd/AlgQ	Transcription
**PsrA**	phhB	3	1	Pterin-4-alpha-carbinolamine dehydratase (EC 4.2.1.96)	
**PsrA**	fabL	3	1	ENOYL-[ACYL-CARRIER-PROTEIN] REDUCTASE (fabL) (NADPH) (EC 1.3.1.9)	Fatty acid biosynthesis
**PsrA**	aceA	12	1	isocitrate lyase	Tricarboxylic acid cycle
**PsrA**	SO0080	9	1	thioesterase superfamily protein	
**PsrA**	mdh	4	1	Malate synthase (EC 2.3.3.9)	Tricarboxylic acid cycle
**PsrA**	ldh	2	1	Leucine dehydrogenase (EC 1.4.1.9)	Branched_chain amino acid biosynthesis
**PsrA**	fadH1	2	1	2,4-dienoyl-CoA reductase [NADPH] (EC 1.3.1.34)	Fatty acid degradation
**PsrA**	fadE3	5	1	acyl-CoA dehydrogenase	Fatty acid degradation
**PsrA**	acdH3	2	2	Acyl-CoA dehydrogenase (EC 1.3.99.3)	Fatty acid degradation
**PsrA**	paaI2	1	1	Phenylacetic acid degradation protein paaI	
**PsrA**	Sbal_0657	4	1	hypothetical protein	
**PsrA**	fabK	1	1	Enoyl-[acyl-carrier-protein] reductase [FMN] (EC 1.3.1.9)	Fatty acid biosynthesis
**PsrA**	SO3908	1	1	Enoyl-CoA hydratase (EC 4.2.1.17)	Fatty acid degradation
**PsrA**	acdH4	1	1	Acyl-CoA dehydrogenase (EC 1.3.99.3)	Fatty acid degradation
**PsrA**	fadL2	1	1	Long-chain fatty acid transport protein	Fatty acid degradation
**PsrA**	fadD3	1	1	long-chain-fatty-acid--CoA ligase	Fatty acid degradation
**RutR**	rutR	52	11	Transcriptional regulator RutR of pyrimidine catabolism, TetR family	Transcription regulation
**RutR**	rutB	13	7	Peroxyureidoacrylate / ureidoacrylate amido hydrolase	Pyrimidine degradation
**RutR**	rutA	12	7	Pyrimidine oxygenase	Pyrimidine degradation
**RutR**	xdhC	22	5	XdhC protein (assists in molybdopterin insertion into xanthine dehydrogenase)	Purine degradation
**RutR**	xdhA	22	5	Xanthine dehydrogenase, iron-sulfur cluster and FAD-binding subunit A (1.17.1.4)	Purine degradation
**RutR**	xdhB	22	5	Xanthine dehydrogenase, molybdenum binding subunit (EC 1.17.1.4)	Purine degradation
**RutR**	guaD	21	5	Guanine deaminase (EC 3.5.4.3)	Purine degradation
**RutR**	rutC	11	6	Aminoacrylate peracid reductase	Pyrimidine degradation
**RutR**	rutD	11	6	Aminoacrylate hydrolase	Pyrimidine degradation
**RutR**	pydC	31	4	Beta-ureidopropionase (EC 3.5.1.6)	Pyrimidine degradation

**RutR**	pydX	29	4	Pyridine nucleotide-disulphide oxidoreductase associated with reductive pyrimidine catabolism	Pyrimidine degradation
**RutR**	pydA	27	4	Dihydropyrimidine dehydrogenase [NADP+] (EC 1.3.1.2)	Pyrimidine degradation
**RutR**	rutF	9	5	Flavin reductase	Pyrimidine degradation
**RutR**	pydB	26	4	Dihydropyrimidinase (EC 3.5.2.2)	Pyrimidine degradation
**RutR**	pbuT	14	4	Xanthine/uracil permease	Nucleoside transport
**RutR**	pucL	15	3	Uricase (EC 1.7.3.3)	Pyrimidine degradation
**RutR**	COG3748	14	3	hypothetical protein, COG3748	
**RutR**	rutE	6	4	3-hydroxy propionic acid dehydrogenase	Pyrimidine degradation
**RutR**	rutR2	3	2	Transcriptional regulator RutR of pyrimidine catabolism, TetR family	Transcription regulation
**RutR**	codA	13	4	Cytosine deaminase (EC 3.5.4.1)	Pyrimidine degradation
**RutR**	pydP	10	3	Pyrimidine permease in reductive pathway	Pyrimidine transport
**RutR**	pucM	9	2	Hydroxyisourate hydrolase (EC 3.5.2.17)	Pyrimidine degradation
**RutR**	upp	9	3	Uracil phosphoribosyltransferase (EC 2.4.2.9)	Pyrimidine degradation
**RutR**	ppuD	10	3	Predicted ABC transporter, inner membrane protein precursor	Nucleoside transport
**RutR**	ppuC	10	3	Predicted ABC transporter, permease protein	Nucleoside transport
**RutR**	ppuA	10	3	Predicted ABC transporter, ATP-binding protein	
**RutR**	allA	9	3	Ureidoglycolate hydrolase (EC 3.5.3.19)	Purine degradation
**RutR**	COG0726	8	2	putative polysaccharide deacetylase family protein	
**RutR**	tsx	9	1	Nucleoside-binding outer membrane protein	Nucleoside transport
**RutR**	cdd	11	3	Cytidine deaminase (EC 3.5.4.5)	Pyrimidine degradation
**RutR**	pbuT2	7	2	Xanthine/uracil permease	Nucleoside transport
**RutR**	deoA	10	3	Thymidine phosphorylase (EC 2.4.2.4)	Pyrimidine degradation
**RutR**	pntB	11	2	Predicted nucleoside ABC transporter, permease protein 1	Nucleoside transport
**RutR**	pntC	11	2	Predicted nucleoside ABC transporter, permease protein 2	Nucleoside transport
**RutR**	pntA	11	2	Predicted nucleoside ABC transporter, ATP-binding protein	Nucleoside transport
**RutR**	rutG	5	2	Uracil permease	Pyrimidine transport
**RutR**	rutG2	2	1	Uracil permease	Pyrimidine transport
**RutR**	gpt	1	1	Xanthine-guanine phosphoribosyltransferase (EC 2.4.2.22)	Purine degradation
**RutR**	xpt	1	1	Xanthine phosphoribosyltransferase (EC 2.4.2.22)	Purine degradation
**RutR**	PF07958	4	1	Conserved hypothetical protein	
**RutR**	ribA2	4	1	GTP cyclohydrolase II (EC 3.5.4.25) homolog	
**RutR**	pntD	10	2	Predicted nucleoside ABC transporter, substrate-binding protein	Nucleoside transport
**RutR**	pytO	10	2	Predicted pyrimidine ABC transporter, permease protein 1	Pyrimidine transport
**RutR**	pytM	10	2	Predicted pyrimidine ABC transporter, substrate-binding protein	Pyrimidine transport
**RutR**	pytN	10	2	Predicted pyrimidine ABC transporter, ATP-binding protein	Pyrimidine transport
**RutR**	pytQ	10	2	Predicted pyrimidine ABC transporter, permease protein 2	Pyrimidine transport
**RutR**	allC	6	3	Allantoicase (EC 3.5.3.4)	Purine degradation
**RutR**	ppuB	6	2	Predicted ABC transporter, substrate-binding protein precursor	
**RutR**	pytC	10	2	Pyrimidine ABC transporter, permease protein 2	Pyrimidine transport
**RutR**	pytB	10	2	Pyrimidine ABC transporter, permease protein 1	Pyrimidine transport
**RutR**	deoC	10	2	Deoxyribose-phosphate aldolase (EC 4.1.2.4)	Pyrimidine degradation
**RutR**	pytA	10	2	Pyrimidine ABC transporter, ATP-binding protein	Pyrimidine transport
**RutR**	carA	6	1	Carbamoyl-phosphate synthase small chain (EC 6.3.5.5)	Arginine and pyrimidine biosynthesis
**RutR**	carB	6	1	Carbamoyl-phosphate synthase large chain (EC 6.3.5.5)	Arginine and pyrimidine biosynthesis
**RutR**	pytD	9	2	Pyrimidine ABC transporter, substrate-binding protein	Pyrimidine transport
**RutR**	deoD	7	1	Purine nucleoside phosphorylase (EC 2.4.2.1)	Purine degradation
**RutR**	nupX	3	1	Nucleoside permease	Nucleoside transport
**RutR**	udk	3	1	Uridine kinase (EC 2.7.1.48)	Pyrimidine metabolism
**RutR**	COG1739	4	1	hypothetical protein, COG1739	

**RutR**	ald	7	1	Aldehyde dehydrogenase (EC 1.2.1.3)	
**RutR**	add	5	2	Adenosine deaminase (EC 3.5.4.4)	Purine degradation
**RutR**	allB	3	2	Allantoinase (EC 3.5.2.5)	Purine degradation
**RutR**	omp1	2	1	putative TonB-dependent outer membrane transporter	Nucleoside transport
**RutR**	omp2	2	1	putative TonB-dependent outer membrane transporter	Nucleoside transport
**RutR**	pbuT3	3	1	Xanthine/uracil permease	Nucleoside transport
**RutR**	ssnA	3	1	Predicted chlorohydrolase/aminohydrolase	
**RutR**	pytH	3	1	Predicted hydrolase	
**RutR**	praX	3	2	Omega-amino acid--pyruvate aminotransferase (EC 2.6.1.18)	
**RutR**	tsx2	2	1	Nucleoside-binding outer membrane protein	Nucleoside transport
**RutR**	codB	1	1	Cytosine permease	Nucleoside transport
**RutR**	nupP	2	1	Predicted purine nucleoside permease	
**RutR**	add2	1	1	Adenosine deaminase (EC 3.5.4.4)	Purine degradation
**RutR**	hpt	2	2	Hypoxanthine-guanine phosphoribosyltransferase (EC 2.4.2.8)	Purine degradation
**RutR**	deoB	3	1	Phosphopentomutase (EC 5.4.2.7)	Purine degradation
**RutR**	RL3717	2	1	Hypothetical protein	
**RutR**	RSP_1242	2	1	Predicted lyase	
**RutR**	tsx3	1	1	Nucleoside-binding outer membrane protein	Nucleoside transport
**RutR**	pbuT4	1	1	Xanthine/uracil permease	Nucleoside transport
**RutR**	uraA	1	1	Uracil permease	Nucleoside transport
**RutR**	rutR3	1	1	Transcriptional regulator RutR of pyrimidine catabolism, TetR family	Transcription regulation
**RutR**	Meso_2056	1	1	Hypothetical protein	
**RutR**	Atu2387	1	1	NTP pyrophosphohydrolase, MutT family	
**RutR**	mll1644	1	1	Predicted methyltransferase	
**RutR**	OG2516_07987	1	1	Conserved hypothetical protein	
**RutR**	Jann_2708	1	1	Hypothetical protein	
**RutR**	Jann_2706	1	1	hypothetical protein	
**RutR**	MED193_05504	1	1	Hypothetical protein	
**RutR**	MED193_05494	1	1	Hypothetical protein	
**RutR**	Jann_0788	1	1	Hypothetical protein	
**RutR**	Jann_0787	1	1	Hypothetical protein	
**RutR**	Jann_2704	1	1	Hypothetical protein	
**RutR**	RSP_0188	1	1	DedA family integral membrane protein	
**RutR**	OB2597_04350	1	1	Hypothetical protein	
**RutR**	Jann_2702	1	1	Predicted N-acetyltransferase	
**RutR**	SKA53_10669	1	1	Hypothetical protein	
**RutR**	RB2654_14945	1	1	Hypothetical protein	
**RutR**	OG2516_10896	1	1	Hypothetical protein	
**RutR**	amiC	1	1	Predicted amidase	
**SahR**	sahR	61	9	Predicted regulator of methionine metabolism, ArsR family	Transcription regulation
**SahR**	ahcY	50	7	Adenosylhomocysteinase (EC 3.3.1.1)	Methionine metabolism
**SahR**	metF	43	7	5,10-methylenetetrahydrofolate reductase (EC 1.5.1.20)	Methionine biosynthesis
**SahR**	metK	41	7	S-adenosylmethionine synthetase (EC 2.5.1.6)	Methionine metabolism
**SahR**	metH	32	6	5-methyltetrahydrofolate--homocysteine methyltransferase (EC 2.1.1.13)	Methionine biosynthesis
**SahR**	metH2	14	4	5-methyltetrahydrofolate--homocysteine methyltransferase (EC 2.1.1.13)	Methionine biosynthesis
**SahR**	metE	8	4	5-methyltetrahydropteroyltriglutamate--homocysteine methyltransferase (EC 2.1.1.14)	Methionine biosynthesis
**SahR**	bhmT	4	2	Betaine--homocysteine S-methyltransferase (EC 2.1.1.5)	Methionine biosynthesis
**SahR**	panC	5	1	Pantoate--beta-alanine ligase (EC 6.3.2.1)	Alanine metabolism

**SahR**	metB	1	1	Cystathionine gamma-synthase (EC 2.5.1.48)	Methionine biosynthesis
**SahR**	metX2	1	1	Homoserine O-acetyltransferase (EC 2.3.1.31)	Methionine biosynthesis
**SahR**	hom	1	1	Homoserine dehydrogenase (EC 1.1.1.3)	Methionine biosynthesis
**SahR**	metE2	1	1	methionine synthase	Methionine biosynthesis
**SahR**	DUF1852	1	1	Protein of unknown function DUF1852	
**SahR**	ddl	1	1	D-alanine--D-alanine ligase B (EC 6.3.2.4)	Alanine metabolism
**SahR**	Caul_3406	1	1	PIN domain protein	
**SahR**	metT	1	1	Methionine transporter MetT	Methionine transport
**SamR**	metE	1	1	5-methyltetrahydropteroyltriglutamate--homocysteine methyltransferase (EC 2.1.1.14)	Methionine biosynthesis
**SamR**	metF2	4	1	5,10-methylenetetrahydrofolate reductase (EC 1.5.1.20)	Methionine biosynthesis
**SamR**	metX2	4	1	Homoserine O-acetyltransferase (EC 2.3.1.31)	Methionine biosynthesis
**SamR**	metB	4	1	Cystathionine gamma-synthase (EC 2.5.1.48)	Methionine biosynthesis
**SamR**	hom	3	1	Homoserine dehydrogenase (EC 1.1.1.3)	Methionine biosynthesis
**SamR**	metK	4	1	S-adenosylmethionine synthetase (EC 2.5.1.6)	SAM biosynthesis
**SamR**	samR	4	1	Transcriptional regulator of methionine metabolism, ArsR family	Transcription regulation
**SamR**	metH1	3	1	5-methyltetrahydrofolate--homocysteine methyltransferase (EC 2.1.1.13)	Methionine biosynthesis
**SamR**	metH2	3	1	5-methyltetrahydrofolate--homocysteine methyltransferase (EC 2.1.1.13)	Methionine biosynthesis
**TrpR**	trpE	36	6	Anthranilate synthase, aminase component (EC 4.1.3.27)	Tryptophan biosynthesis
**TrpR**	trpR	37	6	Trp operon repressor	Transcription regulation
**TrpR**	trpG	12	4	Anthranilate synthase, amidotransferase component (EC 4.1.3.27)	Tryptophan biosynthesis
**TrpR**	trpB	30	4	Tryptophan synthase beta chain (EC 4.2.1.20)	Tryptophan biosynthesis
**TrpR**	trpC	27	3	Indole-3-glycerol phosphate synthase (EC 4.1.1.48)	Tryptophan biosynthesis
**TrpR**	trpA	29	3	Tryptophan synthase alpha chain (EC 4.2.1.20)	Tryptophan biosynthesis
**TrpR**	mtr	17	3	Tryptophan-specific transport protein	Tryptophan transport
**TrpR**	trpD	24	2	Anthranilate phosphoribosyltransferase (EC 2.4.2.18)	Tryptophan biosynthesis
**TrpR**	trpD_a	10	1	Anthranilate synthase, amidotransferase component (EC 4.1.3.27)	Tryptophan biosynthesis
**TrpR**	trpD_b	10	1	Anthranilate phosphoribosyltransferase (EC 2.4.2.18)	Tryptophan biosynthesis
**TrpR**	tyrA	16	1	Chorismate mutase I (EC 5.4.99.5) / Prephenate dehydrogenase [EC:5.4.99.5 1.3.1.12]	Tyrosine & Phenylanaline biosynthesis
**TrpR**	COG1541	1	1	Coenzyme F390 synthetase	
**TrpR**	aroF	16	1	2-keto-3-deoxy-D-arabino-heptulosonate-7-phosphate synthase (EC 2.5.1.54)	Aromatic amino acid biosynthesis
**TrpR**	SSF55729	1	1	Acyl-CoA N-acyltransferase	
**TrpR**	COG0733(Trp)	8	1	Predicted tryptophan transporter, SNF family	Tryptophan transport
**TrpR**	aroG	6	1	2-keto-3-deoxy-D-arabino-heptulosonate-7-phosphate synthase (EC 2.5.1.54)	Aromatic amino acid biosynthesis
**TrpR**	aroH	6	1	2-keto-3-deoxy-D-arabino-heptulosonate-7-phosphate synthase (EC 2.5.1.54)	Aromatic amino acid biosynthesis
**TrpR**	aroM	4	1	AroM family protein	Aromatic amino acid biosynthesis
**TrpR**	aroL	4	1	Shikimate kinase III (EC 2.7.1.71)	Aromatic amino acid biosynthesis
**TrpR**	yaiA	4	1	putative cytoplasmic protein	
**TrpR**	aroA	3	1	5-Enolpyruvylshikimate-3-phosphate synthase (EC 2.5.1.19)	Aromatic amino acid biosynthesis
**TrpR**	COG4221	2	1	Short-chain alcohol dehydrogenase of unknown specificity	
**TrpR**	HI1388	2	1	Anthranilate synthase, amidotransferase component (EC 4.1.3.27)	Tryptophan biosynthesis
**TrpR**	tnaB	2	1	Tryptophan-specific transport protein	Tryptophan transport
**TrpR**	aroF2	2	1	2-keto-3-deoxy-D-arabino-heptulosonate-7-phosphate synthase (EC 2.5.1.54)	Aromatic amino acid biosynthesis
**TrpR**	HAPS_1139	1	1	ABC transporter, inner-membrane component	
**TrpR**	HAPS_0395	1	1	ABC transporter, substrate binding component	
**TrpR**	HAPS_1138	1	1	ABC transporter, ATP-binding protein	
**TrpR**	tnaA	1	1	Tryptophanase (EC 4.1.99.1)	Tryptophan utilization
**TrpR**	trpB2	1	1	Tryptophan synthase beta chain like (EC 4.2.1.20)	Tryptophan utilization
**TyrR**	phhA	41	5	Phenylalanine-4-hydroxylase (EC 1.14.16.1)	Phenylalanine degradation
**TyrR**	phhB	38	5	Pterin-4-alpha-carbinolamine dehydratase (EC 4.2.1.96)	Phenylalanine degradation

**TyrR**	tyrA	39	5	Chorismate mutase I (EC 5.4.99.5) / Prephenate dehydrogenase [EC:5.4.99.5 1.3.1.12]	Tyrosine & Phenylanaline biosynthesis
**TyrR**	tyrR	45	6	Transcriptional regulator of aromatic amino acid biosynthesis	Transcription regulation
**TyrR**	aroF	35	5	2-keto-3-deoxy-D-arabino-heptulosonate-7-phosphate synthase (EC 2.5.1.54)	Aromatic amino acid biosynthesis
**TyrR**	COG2814	7	1	Predicted tyrosine transporter, COG2814 family	Tyrosine transport
**TyrR**	emrD	9	1	multidrug resistance protein D	
**TyrR**	pepD	9	1	Aminoacyl-histidine dipeptidase (Peptidase D) (EC 3.4.13.3)	
**TyrR**	putA	9	1	Proline dehydrogenase (EC 1.5.99.8) (Proline oxidase) / Delta-1-pyrroline-5-carboxylate dehydrogenase (EC 1.5.1.12)	Proline degradation
**TyrR**	kyn	8	1	kynureninase (tryptophan degradation)	Tryptophan degradation
**TyrR**	aroM	6	1	AroM family protein	Aromatic amino acid biosynthesis
**TyrR**	SO1117	8	1	peptidase M17, leucyl aminopeptidase	
**TyrR**	tdo	8	1	tryptophan 2,3-dioxygenase	Tryptophan degradation
**TyrR**	mtr	5	1	Tryptophan-specific transport protein	Tryptophan transport
**TyrR**	yaiA	5	1	hypothetical protein	
**TyrR**	aroG	5	2	2-keto-3-deoxy-D-arabino-heptulosonate-7-phosphate synthase I alpha (EC 2.5.1.54)	Aromatic amino acid biosynthesis
**TyrR**	COG1284	4	1	Hypothetical protein	
**TyrR**	Sfri_3409	6	1	Hypothetical protein	
**TyrR**	aprE	6	1	Alkaline serine protease	
**TyrR**	Sfri_3410	6	1	transcriptional regulator, XRE family protein	
**TyrR**	ompF	4	1	outer membrane porin F	
**TyrR**	ipdC	7	2	Indole-3-pyruvate decarboxylase (EC 4.1.1.74)	Tryptophan degradation
**TyrR**	prlC	4	1	Oligopeptidase A (EC 3.4.24.70)	
**TyrR**	pep1	3	1	Alkaline serine exoprotease A precursor (EC 3.4.21.-)	
**TyrR**	aprE2	3	1	Cold-active alkaline serine protease (EC 3.4.21.62)	
**TyrR**	omp2	3	1	putative TonB-dependent outer membrane receptor	
**TyrR**	tpl	3	2	Tyrosine phenol-lyase (EC 4.1.99.2)	Tyrosine degradation
**TyrR**	folA	1	1	Dihydrofolate reductase (EC 1.5.1.3)	Tetrahydrofolate biosynthesis
**TyrR**	pep2	1	1	peptidase M4 thermolysin	
**TyrR**	omp1	1	1	TonB-dependent receptor	
**TyrR**	pep4	1	1	prolyl oligopeptidase family protein	
**TyrR**	tyrP	32	5	Tyrosine-specific transport protein	Tyrosine transporter
**TyrR**	hmgB	29	5	Maleylacetoacetate isomerase (EC 5.2.1.2)	Tyrosine degradation
**TyrR**	hmgC	29	5	Fumarylacetoacetase (EC 3.7.1.2)	Tyrosine degradation
**TyrR**	hpd	14	4	4-hydroxyphenylpyruvate dioxygenase (EC 1.13.11.27)	Tyrosine degradation
**TyrR**	hmgA	13	4	Homogentisate 1,2-dioxygenase (EC 1.13.11.5)	Tyrosine degradation
**TyrR**	aceA	12	1	Isocitrate lyase (EC 4.1.3.1)	Tricarboxylic acid cycle
**TyrR**	aceB	16	1	Malate synthase (EC 2.3.3.9)	Tricarboxylic acid cycle
**TyrR**	acsA	8	1	Acetoacetyl-CoA synthetase (EC 6.2.1.16)	Acetyl-coenzyme A synthetase
**TyrR**	aroA	14	1	5-Enolpyruvylshikimate-3-phosphate synthase (EC 2.5.1.19)	Aromatic amino acid biosynthesis
**TyrR**	aroL	10	1	Shikimate kinase III (EC 2.7.1.71)	Aromatic amino acid biosynthesis
**TyrR**	aroP	11	1	Aromatic amino acid transport protein AroP	Aromatic amino acid transport
**TyrR**	bkdA1	16	1	Branched-chain alpha-keto acid dehydrogenase, E1 component, alpha subunit (EC 1.2.4.4)	Branched_chain amino acid degradation
**TyrR**	bkdA2	16	1	Branched-chain alpha-keto acid dehydrogenase, E1 component, beta subunit (EC 1.2.4.4)	Branched_chain amino acid degradation
**TyrR**	bkdB	16	1	Dihydrolipoamide acyltransferase component of branched-chain alpha-keto acid dehydrogenase complex (EC 2.3.1.168)	Branched_chain amino acid degradation
**TyrR**	brnQ	16	1	Branched-chain amino acid transport system carrier protein	Branched_chain amino acid transport
**TyrR**	COG0733(Tyr)	13	2	Predicted tyrosine transporter, SNF family	
**TyrR**	tyrR2	2	1	Tyrosine and phenylalanine degradation transcriptional activator, TyrR family	Transcription regulation
**TyrR**	ivdA	16	1	3-ketoacyl-CoA thiolase [isoleucine degradation] (EC 2.3.1.16)	Branched_chain amino acid degradation

**TyrR**	ivdB	16	1	Methylmalonate-semialdehyde dehydrogenase (EC 1.2.1.27)	Branched_chain amino acid degradation
**TyrR**	ivdC	16	1	Branched-chain acyl-CoA dehydrogenase (EC 1.3.99.12)	Branched_chain amino acid degradation
**TyrR**	ivdD	16	1	Enoyl-CoA hydratase [valine degradation] (EC 4.2.1.17) / Enoyl-CoA hydratase [isoleucine degradation] (EC 4.2.1.17)	Branched_chain amino acid degradation
**TyrR**	ivdE	16	1	3-hydroxyisobutyryl-CoA hydrolase (EC 3.1.2.4)	Branched_chain amino acid degradation
**TyrR**	ivdF	16	1	3-hydroxyisobutyrate dehydrogenase (EC 1.1.1.31)	Branched_chain amino acid degradation
**TyrR**	ivdG	16	1	3-hydroxyacyl-CoA dehydrogenase [isoleucine degradation] (EC 1.1.1.35)	Branched_chain amino acid degradation
**TyrR**	ldh	16	1	Leucine dehydrogenase (EC 1.4.1.9)	Branched_chain amino acid degradation
**TyrR**	liuA	14	1	Isovaleryl-CoA dehydrogenase (EC 1.3.99.10)	Branched_chain amino acid degradation
**TyrR**	liuB	14	1	Methylcrotonyl-CoA carboxylase carboxyl transferase subunit (EC 6.4.1.4)	Branched_chain amino acid degradation
**TyrR**	liuC	14	1	Methylglutaconyl-CoA hydratase (EC 4.2.1.18)	Branched_chain amino acid degradation
**TyrR**	liuD	16	1	Methylcrotonyl-CoA carboxylase biotin-containing subunit (EC 6.4.1.4)	Branched_chain amino acid degradation
**TyrR**	liuE	14	1	Hydroxymethylglutaryl-CoA lyase (EC 4.1.3.4)	Branched_chain amino acid degradation
**TyrR**	liuF	13	1	Succinyl-CoA:3-ketoacid-coenzyme A transferase subunit A (EC 2.8.3.5)	Branched_chain amino acid degradation
**TyrR**	liuG	13	1	Succinyl-CoA:3-ketoacid-coenzyme A transferase subunit B (EC 2.8.3.5)	Branched_chain amino acid degradation
**TyrR**	liuR	14	1	Predicted transcriptional regulator LiuR of leucine degradation pathway, MerR family	Transcription regulation
**TyrR**	mdeA	14	2	Methionine gamma-lyase (EC 4.4.1.11)	Methionine degradation
**TyrR**	pep3	14	1	peptidase, M13 family	
**TyrR**	phhC	5	1	Aromatic-amino-acid aminotransferase (EC 2.6.1.57)	Phenylalanine degradation
**TyrR**	phhR	7	1	Phenylalanine degradation transcriptional activator, TyrR family	Transcription regulation
**TyrR**	tyrB	16	1	Tyrosine aminotransferase (EC 2.6.1.42)	Tyrosine biosynthesis / Tyrosine degradation

[1] Number of regulatory interactions

[2] Number of taxa with regulation

Table S4. Metabolic and gene content of reconstructed TF regulons in Proteobacteria classified by conservation of regulatory interactions.

TF name	TF regulon members	Assigned metabolic pathway or process [1]	Major function	TF effector
**ArgR**	Core		**Arginine metabolism**	**Arginine**
	argH, argB, argC, argG, argF, argA, argE	Arginine biosynthesis		
	argR	Transcription regulation		
	artI, artQ, artM, artP	Arginine transport		
	astD, astA	Arginine degradation		
	carA, carB	Arginine and pyrimidine biosynthesis		
	Taxonomy-specific			
	argD	Arginine biosynthesis		
	argW, artJ, omp	Arginine transport		
	gltB, gltD	Glutamate biosynthesis		
	ilvM, ilvG, ilvD, ilvA	Branched-chain amino acid biosynthesis		
	potF, potG, potH, potI	Putrescine transport		
	recN	DNA repair		
	oadA, oadB, oadG	Pyruvate metabolism		
	astC, astB	Arginine degradation		
	Genome-specific			
	ilvE	Branched-chain amino acid biosynthesis		
	hisJ, hisM, hisP, hisQ	Histidine transport		
	arcA, arcB, arcC, arcD, astE	Arginine degradation		
	hisA, hisB, hisC, hisD, hisF, hisG, hisH, hisI	Histidine biosynthesis		
	proV, proW, proX	Proline transport		
	speF, potE	Putrescine metabolism		
**BioR**	All target genes		**Biotin biosynthesis**	**Unknown**
	bioY, bioM, bioN	Biotin transport		
	bioB, bioF, bioD, bioA, bioZ, bioG, bioC	Biotin biosynthesis		
	bioR	Transcription regulation		
**BirA**	Core		**Biotin biosynthesis**	**Biotin**
	bioB, bioF, bioD, bioC, bioA	Biotin biosynthesis		
	Taxonomy-specific			
	bioH	Biotin biosynthesis		
	birA	Transcription regulation		
	fabF, fabG	Fatty acid biosynthesis		
	Genome-specific			
	yigM	Biotin transport		
	bioW	Biotin biosynthesis		
**FabR**	Core		**Fatty acid biosynthesis**	**Unsaturated acyl-ACP**
	OLE1 (desA)	Unsaturated fatty acid biosynthesis		
	fabA, fabB, lcfH	Fatty acid biosynthesis		
	plsC	Glycerolipid metabolism		
	hylII	Fatty acid metabolism		
	Taxonomy-specific			
	desB, desC	Unsaturated fatty acid biosynthesis		
	fadL, lcfE	Fatty acid biosynthesis		
	fabR, fabR2	Transcription regulation		
	Genome-specific			
	pfaA, pfaB, pfaC, pfaD	Unsaturated fatty acid biosynthesis		
	pfaR, psrA	Transcription regulation		
	fadE	Fatty acid degradation		

**FadP**	Core		**Fatty acid degradation**	**Unknown**
	fadA, fadB, acdA, acdB, acdH, echH, acdP, acdQ, fadD	Fatty acid degradation		
	etfA, etfB, etfD	Electron transfer chain for fatty acid degradation		
	pncA	Nicotinate biosynthesis		
	fadP	Transcription regulation		
	Taxonomy-specific and Genome-specific			
	acsA	Acetyl-coenzyme A synthetase		
	BPSL1236	Glycolysis		
	liuR	Transcription regulation		
	paaI, paaG4, paaH1, bktB, alkK	Fatty acid degradation		
**FadR**	Core		**Fatty acid degradation**	**Palmitoyl-CoA; Oleoyl-CoA**
	fadL, fadI, fadJ, fadE	Fatty acid degradation		
	Taxonomy-specific			
	fabA, fabB	Fatty acid biosynthesis		
	plsB	Glycerolipid metabolism		
	fadA, fadD, fadH, fadB, fadM, SO0572	Fatty acid degradation		
	fadR, iclR	Transcription regulation		
	Genome-specific			
	acdB, tesB	Fatty acid degradation		
**GlcC**	Core		**Glycolate utilization**	**Glycolate**
	glcE, glcF, glcD, glcG	Glycolate utilization		
	glcC	Transcription regulation		
	Taxonomy-specific			
	glcB	Tricarboxylic acid cycle		
	glcA	Glycolate transport		
	lldD	Lactate utilization		
	Genome-specific			
	glcQ, glcM, glcP	Glycolate transport		
	lldG, lldE, lldF, ykgE, ykgF, ykgG, lldP	Lactate utilization		
	lysR	Transcription regulation		
**HexR**	Core		**Central carbohydrate metabolism**	**2-keto-3-deoxy-6-phosphogluconate**
	glk, pykA	Glycolysis		
	edd, eda	Entner-Doudoroff pathway		
	zwf, pgl	Pentose phosphate pathway		
	hexR	Transcription regulation		
	Taxonomy-specific			
	tal, phk, gnd	Pentose phosphate pathway		
	nqrD, nqrC, nqrF, nqrA, nqrE, nqrB	Electron transport chain		
	adhE, pflA, pflB, ackA, pta, focA	Fermentation		
	ppsA	Gluconeogenesis		
	ptsI, ptsH, ptsG, crr	Glucose transport		
	gltD, gltB	Glutamate biosynthesis		
	gcvT, gcvP, gcvH	Glycine cleavage system		
	gapA, pgi, ppc, tpiA, gapB, gpmM	Glycolysis		
	mtlA, mtlD	Mannitol utilization		
	pntB, pntA	NAD metabolism		
	nirB, nirD	Nitrogen metabolism		
	deoD, deoA, deoB, nupC	Nucleoside metabolism		
	mtlR, gltR, gltS	Transcription regulation		

	aceB, aceA	Tricarboxylic acid cycle		
	Genome-specific			
	bkdA2, bkdB, bkdA1	Branched-chain amino acid degradation		
	adhB, ldhA	Fermentation		
	mglA, mglB, mglC	Galactose transport		
	gntU, gntK	Gluconate utilization		
	pckA	Gluconeogenesis		
	ptsHI	Glucose transport		
	glpT	Glycerol-3-phosphate transport		
	glgX, glgA, glgC, glgP	Glycogen utilization		
	gapN, eno, pgk, fba, aldE	Glycolysis		
	lctP, dld	L-lactate utilization		
	manC	Mannose utilization		
	cdd	Nucleoside metabolism		
**HmgQ**	All target genes		**Tyrosine degradation**	**Unknown**
	hmgA, hmgB, hmgC, hpd, gloA	Tyrosine degradation		
	hmgQ	Transcription regulation		
**HmgR**	All target genes		**Tyrosine degradation**	**Homogentisate**
	hmgA, hmgB, hmgC, hpd	Tyrosine degradation		
	COG2814	Tyrosine transport		
	hmgR	Transcription regulation		
**HmgS**	All target genes		**Tyrosine degradation**	**Unknown**
	hmgA, hmgB	Tyrosine degradation		
	hmgS	Transcription regulation		
**HutC**	Core		**Histidine utilization**	**Urocanate**
	hutU, hutH, hutI, hutD, hutF, hutG, hutG2	Histidine degradation		
	hutC	Transcription regulation		
	Taxonomy-specific			
	hisT, hisX, hisY, hisZ, hisP, hisQ, hisM, hisJ, COG2814, omp	Histidine transport		
	Genome-specific			
	hutV, hutW, hutX, COG3314	Histidine transport		
	hutH2	Histidine degradation		
	hisA, hisB, hisC, hisD, hisF, hisG, hisH, hisI	Histidine biosynthesis		
**HypR**	Core		**Proline and 4-hydrohyproline utilization**	**Proline; 4-hydroxyproline**
	hypD, hypE, hypH, hypO	Hydroxyproline/proline degradation		
	hypR	Transcription regulation		
	Taxonomy-specific			
	hypY, hypH'-2	Hydroxyproline/proline degradation		
	putA, prdP, ampP	Proline degradation		
	hypP, hypM, hypN, hypQ	Hydroxyproline transport		
	COG531, omp	Hydroxyproline/proline transport		
	hypX, hypS	TCA cycle		
	colA2	Collagen degradation		
	Genome-specific			
	pdtP	Proline transport		
	hypA, hypB, hypC, hypT	Hydroxyproline transport		

	hypD', hypH'	Hydroxyproline/proline degradation		
	colA1, ypdF, colA3	Collagen degradation		
**LiuQ**	Core		**Branched-chain amino acid degradation**	**Unknown**
	liuA, liuB, liuC, liuD	Branched-chain amino acid degradation		
	liuQ	Transcription regulation		
	Taxonomy-specific and Genome-specific			
	liuE, aacS, ivd2	Branched-chain amino acid degradation		
	acsA	Acyl-coenzyme A synthetase		
**LiuR**	Core		**Branched-chain amino acid degradation**	**Unknown**
	liuA, liuB, liuC, liuD, liuE, ivdA, ivdC, acdH	Branched-chain amino acid degradation		
	liuR	Transcription regulation		
	etfA, etfB	Electron transfer chain for branched-chain amino acid degradation		
	Taxonomy-specific			
	aacS, acdA, acdB, acdL, acdP, acdQ, bkdA, bkdB, hbdA, ivdB, ivdD, ivdE, ivdF, ivdG, ldh, liuF, liuG, mcm, paaH, echH	Branched-chain amino acid degradation		
	etfD	Electron transfer chain for branched-chain amino acid degradation		
	atuC, atuD, atuE, atuF	Acyclic terpenes degradation		
	gltB, gltD	Glutamate biosynthesis		
	prpB, prpC, prpD	Propionate metabolism		
	fadD	Fatty acid degradation		
	aceB, mdh, sucA, sucB, sucC, sucD, aceK	Tricarboxylic acid cycle		
	thrA, thrB, thrC	Threonine biosynthesis		
	cah	Carbonic anhydrase		
	Genome-specific			
	livF, livG, livH, livK, livM	Branched-chain amino acid transport		
	mmgB	Branched-chain amino acid degradation		
	fadA, fadB, fadL, lcfA	Fatty acid degradation		
	fabG	Fatty acid biosynthesis		
	serA	Serine biosynthesis		
	acs, acsA	Acyl-coenzyme A synthetase		
	aceA	Tricarboxylic acid cycle		
	tyrR	Transcription regulation		
**LldR**	Core		**Lactate utilization**	**Lactate**
	dld, lldD, lldE, lldF, lldG	Lactate utilization		
	lldP	Lactate transport		
	lldR	Transcription regulation		
	Taxonomy-specific			
	lldX	Lactate transport		
	glcF, glcD	Glycolate utilization		
	Genome-specific			
	glcE, glcG	Glycolate utilization		
	glcB	Tricarboxylic acid cycle		
**MetJ**	Core		**Methionine metabolism**	**S-adenosyl-methionine**
	metI, metN, metQ, metT	Methionine transport		
	metJ, metR	Transcription regulation		
	metA, metB, metE, metF, metH, metK, metL	Methionine biosynthesis		

	btuB	Vitamin B12 transport		
	Taxonomy-specific			
	mtsA, mtsB, mtsC	Methionine transport		
	metX, metY	Methionine biosynthesis		
	msrA, csd	Methionine metabolism		
	Genome-specific			
	btuC, btuD, btuF	Vitamin B12 transport		
	asd, mccA, mccB, mdeA2, metC, metF-II, mmuM	Methionine biosynthesis		
	mmuP	Methionine transport		
	mtnA, mtnB, mtnC, mtnD, mtnE, mtnK, mtnX, mtnY, mtnZ	Methylthioribose recycling		
	pduO	Vitamin B12 biosynthesis		
	serA	Serine biosynthesis		
	thrA, thrB, thrC	Threonine biosynthesis		
**MetR**	Core		**Methionine metabolism**	**Homocysteine**
	metE	Methionine biosynthesis		
	metR	Transcription regulation		
	Taxonomy-specific			
	metF, glyA, metH, metA, metF-II	Methionine biosynthesis		
	luxS	SAM recycling		
	hmp	Nitric oxide cell defense		
	ilvI, ilvH	Branched-chain amino acid biosynthesis		
	Genome-specific			
	gcvP, gcvH	Glycine cleavage system		
	metQ2	Methionine transport		
	thrC	Threonine biosynthesis		
	metC, hom, mdeA, bhmT	Methionine biosynthesis		
**NadR**	All target genes		**NAD metabolism**	**NAD**
	pnuC, niaP	Niacin or Ribosyl nicotinamide transport (NAD salvage)		
	nadA, nadB	NAD biosynthesis		
	nadR, pncB	NAD salvage		
**NadQ**	Core		**NAD metabolism**	**Unknown**
	nadA, nadC, nadB	NAD biosynthesis		
	Taxonomy-specific			
	nadE, nadD	NAD biosynthesis		
	proA	Proline biosynthesis		
	nadQ	Transcription regulation		
**NagC**	Core		**N-acetylglucosamine utilization**	**N-acetylglucosamine**
	nagA, nagB, nagE	N-acetylglucosamine utilization		
	ptsI, ptsH, crr	Sugar transport		
	nagC	Transcription regulation		
	Taxonomy-specific			
	eno, pgk, fbaA	Glycolysis		
	omp, glmU, glmS, nagD, nagF	N-acetylglucosamine utilization		
	manX, manZ, manY, ptsG	Mannose and glucose transport		
	chiA, hex	Chitin degradation		
	mcp	Chemotaxis protein (toward chitin?)		
	Genome-specific			
	chbB, chbA, chbC, chbF, chiP, ompC, ybfM	Chitobiose utilization		
	chi, cbp, chiD, chi1	Chitin degradation		

	galP	Galactose transport		
	gapA, gapB	Glycolysis		
	chbR, chiS, alsR	Transcription regulation		
	glgA, glgC	Glycogen metabolism		
	gdhA	Glutamate degradation		
	gltA	Tricarboxylic acid cycle		
	nanM, nanC	N-acetylneuraminic acid transport		
**NagQ**	Core		**N-acetylglucos-amine utilization**	**Unknown**
	nagA, nagB2, nagE	N-acetylglucosamine utilization		
	nagQ	Transcription regulation		
	Taxonomy-specific			
	chiA, cdxA, cbp21, chiC, chi, hex	Chitin degradation		
	nagB, nagK, nagZ, nagT, nagV, nagU, nagW, nagP	N-acetylglucosamine utilization		
	ybfM, omp_nag, omp1	Chitobiose utilization		
	murQ	N-acetylmuramic acid utilization		
	ptsI	Sugar transport		
	nagR	Transcription regulation		
	Genome-specific			
	nagX, nagM, nagO, nagN, nagL, nagK2	N-acetylglucosamine utilization		
	anaG	Alpha-N-acetylglucosaminidase		
**NagR**	Core		**N-acetylglucos-amine utilization**	**N-acetylglucos-amine-6-phosphate**
	nagA, nagK, nagB, nagB2, nagP, nagX	N-acetylglucosamine utilization		
	hex, omp_nag	Chitobiose utilization		
	chiA	Chitin degradation		
	nagR	Transcription regulation		
	Taxonomy-specific			
	nagK2	N-acetylglucosamine utilization		
	chiD	Chitin degradation		
	nixC, naxA	Chitobiose utilization		
	pgi2	Glycolysis		
	pckA	Gluconeogenesis		
	Genome-specific			
	cbp, cbp2, cdxA, chiA3	Chitin degradation		
	nixD, nixB, nixA, omp_nag2	Chitobiose utilization		
	bglX	Glucosides utilization		
	SO0852, SO0850, SO0854, SO0853	Fimbriae biogenesis		
	pdaA	N-acetylglucosamine utilization		
	mcp	Chemotaxis protein (toward chitin?)		
**NrdR**	Core		**Deoxyribonucleotid e biosynthesis**	**Deoxyribonucleotide s**
	nrdA, nrdB, nrdD, nrdG	Deoxyribonucleotide biosynthesis		
	Taxonomy-specific			
	nrdJ, nrdH, nrdF, nrdI, nrdE	Deoxyribonucleotide biosynthesis		
	topA	Replication		
	yfaE, SO2417, trxA	Oxidoreductase		
**NrtR**	Core		**NAD metabolism**	**Adenosine diphosphate ribose**
	nrtR	Transcription regulation		
	Taxonomy- and Genome-specific			
	pncB, pncA, nadV, nadE, nadD, nadM, nadR, pnuC, prs	NAD biosynthesis; NAD salvage		

**NtrC**	Core		**Nitrogen assimilation**	**Phosphorylated NtrB**
	glnA	Glutamine biosynthesis		
	amtB	Nitrogen source transport		
	ntrB, glnB, glnK	Nitrogen metabolism regulation proteins		
	ntrC	Transcription regulation		
	Taxonomy-specific			
	dat	Proline degradation		
	narK, nrtC, nrtB, nrtA, gltJ, gltK, gltL, gltI, dppC, dppA, dppB, dppD, dppF	Nitrogen source transport		
	ntrXY	Transcription regulation		
	nasD, nasE, ntrY, ntrX, nasA, gdhA, nasBA, nasB, nifEN	Nitrogen metabolism		
	Genome-specific			
	ureD, ureA, ureB, ureC, ureE, ureG, ureF, ureJ, hmp, uahA, uahB, uahC, nirA, atzF	Nitrogen metabolism		
	hisQ, hisJ, hisM, glnH, glnQ, uctA, uctB, uctC, alsT, potG, potI, potH, potA, potB, potC, potD,	Nitrogen source transport		
	gltB, gltD	Glutamate biosynthesis		
	speB, ygjG	Putrescine metabolism		
	astD, astB, astA, astC, astE	Arginine degradation		
	rutC, rutA, rutF, rutD, rutE, rutE2	Pyrimidine degradation		
	ansA	Asparagine degradation		
	hmp	Nitrogen stress response		
	nac, nasT, rutR	Transcription regulation		
**PdhR**	Core		**Pyruvate metabolism**	**Pyruvate**
	aceE, aceF, lpdA	Pyruvate utilization		
	pdhR	Transcription regulation		
	Taxonomy-specific			
	ndh	NAD metabolism		
	aceB, aceA	Tricarboxylic acid cycle		
	oadB, oadA, oadG	Pyruvate metabolism		
	pflA, pflB	Formate metabolism		
	Genome-specific			
	sdhC, gltA, sdhA, sdhD, sdhB	Tricarboxylic acid cycle		
	cyoC, cyoD, cyoB, cyoA	Electron transfer chain		
	lldP, dld	Lactate metabolism		
	ppc, grcA	Pyruvate metabolism		
	hemL, cyoE	Porphyrin biosynthesis		
	glcB, glcD, glcG, glcE, glcF	Glycolate utilization		
**PsrA**	Core		**Fatty acid degradation**	**Oleate**
	fadA, fadB	Fatty acid degradation		
	psrA	Transcription regulation		
	Taxonomy-specific			
	fadD, fadE, fadH, fadJ, fadI, fadL, acdH, echH, acdB, acdA	Fatty acid degradation		
	fabG, fabF, fabH, fabD, fabL	Fatty acid biosynthesis		
	aceA, aceB, gltA, sdhA, sdhB, sdhC, sdhD	Tricarboxylic acid cycle		
	etfD, etfA, etfB	Electron transfer chain for fatty acid degradation		
	rpoS, algQ	Transcription		
	aroQ	Aromatic amino acid biosynthesis		
	Genome-specific			
	ldh	Branched-chain amino acid biosynthesis		

	mdh	Tricarboxylic acid cycle		
	SO3908	Fatty acid degradation		
	fabK	Fatty acid biosynthesis		
**RutR**	Core		**Pyrimidine utilization**	**Uracil**
	rutB, rutA, rutC, rutD	Pyrimidine degradation		
	rutR	Transcription regulation		
	Taxonomy-specific			
	carA, carB	Arginine and pyrimidine biosynthesis		
	rutG, pydP	Pyrimidine transport		
	xdhC, xdhA, xdhB, guaD, gpt, xpt, deoD	Purine degradation		
	rutF, pydC, pydX, pydA, pydB, rutE, codA, upp, cdd, deoA, pucM, pucL	Pyrimidine degradation		
	pbuT, pntB, pntC, pntA, ppuD, ppuC, pntD	Nucleoside transport		
	Genome-specific			
	pytO, pytM, pytN, pytQ, pytC, pytB, pytA, pytD	Pyrimidine transport		
	add, allB, allA, allC, hpt, deoB	Purine degradation		
	deoC	Pyrimidine degradation		
**SahR**	Core		**Methionine metabolism**	**S-adenosyl-homocysteine**
	ahcY	Methionine metabolism		
	metF, metH	Methionine biosynthesis		
	sahR	Transcription regulation		
	metK	Methionine metabolism		
	Taxonomy-specific			
	metE, metH2	Methionine biosynthesis		
	Genome-specific			
	metT	Methionine transport		
	bhmT, metB, metX, hom	Methionine biosynthesis		
**SamR**	All target genes		**Methionine metabolism**	**Unknown**
	metE, metF2, metX2, metB, metH, hom	Methionine biosynthesis		
	samR	Transcription regulation		
	metK	SAM biosynthesis		
**TyrR**	Core		**Tyrosine metabolism**	**Tyrosine**
	aroF	Chorismate biosynthesis		
	tyrA	Tyrosine & Phenylanaline biosynthesis		
	phhA, phhB	Phenylalanine degradation		
	hmgB, hmgC, hpd, hmgA	Tyrosine degradation		
	tyrP	Tyrosine transporter		
	tyrR (phhR)	Transcription regulation		
	Taxonomy-specific			
	brnQ	Branched-chain amino acid transport		
	mdeA	Methionine degradation		
	putA	Proline degradation		
	bkdA, bkdB, ivdA, ivdB, ivdC, ivdD, ivdE, ivdF, ivdG, ldh, liuA, liuB, liuC, liuD, liuE, liuF, liuG	Branched-chain amino acid degradation		
	hmgR, liuR	Transcription regulation		
	tyrB	Tyrosine biosynthesis / Tyrosine degradation		
	aceA, aceB	Tricarboxylic acid cycle		
	aroA, aroL	Chorismate biosynthesis		

	aroP	Aromatic amino acid transport		
	acsA	Acetyl-coenzyme A synthetase		
	phhC	Phenylalanine degradation		
	COG0733	Tyrosine transport		
	Genome-specific			
	aroM, aroG	Chorismate biosynthesis		
	kyn, tdo, ipdC	Tryptophan degradation		
	mtr	Tryptophan transport		
	tpl	Tyrosine degradation		
	COG2814	Tyrosine transport		
	folA	Tetrahydrofolate biosynthesis		
**TrpR**	Core		**Tryptophan biosynthesis**	**Tryptophan**
	trpE	Tryptophan biosynthesis		
	trpR	Transcription regulation		
	Taxonomy-specific			
	aroF, aroG	Chorismate biosynthesis		
	trpG, trpB, trpC, trpA, trpD	Tryptophan biosynthesis		
	tyrA	Tyrosine biosynthesis		
	mtr, COG0733	Tryptophan transport		
	Genome-specific			
	aroH, aroM, aroL, aroA	Chorismate biosynthesis		
	tnaA, tnaB	Tryptophan transport and degradation		

[1] **annotated gene functions and metabolic pathways are listed in Table S3. This table excludes functionally unassigned genes.**

Table S5. (A) Content of reconstructed TyrR (PhhR), HmgR, HmgQ, HmgS regulons for aromatic amino acid metabolism in gamma-proteobacteria.

Enterobacteriales	TyrR	AroL	AroH	AroG	AroF	TyrA	TyrB	AroP	TyrP	Mtr	COG2814	Tpl					IpdC	TyrR regulon
Escherichia coli K-12	+	+	+	+	+	+	+	+	+	+	-	-					-	aroF-tyrA; tyrP; tyrR; aroP; aroL-yaiA-aroM; mtr
Salmonella typhimurium LT2	+	+	+	+	+	+	+	+	+	+	-	-					+	aroF-tyrA; tyrP; tyrR; aroP; aroL-yaiA-aroM; mtr; ipdC
Citrobacter koseri ATCC BAA-895	+	+	+	+	+	+	+	+	+	+	+	+					+	aroF-tyrA; tyrP; tyrR; aroP; aroL-yaiA-aroM; mtr; COG2814; tpl; ipdC
Klebsiella pneumoniae MGH 78578	+	+	+	+	+	+	+	+	+	+	+	-					+	aroF-tyrA; tyrP; tyrR; aroP; aroL-yaiA-aroM; mtr; COG2814; ipdC
Enterobacter sp. 638	+	+	+	+	+	+	+	+	+	+	+	-					-	aroF-tyrA; tyrP; tyrR; aroP; aroL-yaiA-aroM; mtr; COG2814
Erwinia amylovora ATCC 49946	+	+	-	+	+	+	+	+	-	+	-	-					+	aroF-tyrA; tyrR; aroP; aroL-yaiA-aroM
Yersinia pestis KIM	+	+	+	+	+	+	+	+	+	+	+	-					-	aroF-tyrA; tyrP; aroP; aroL; COG2814
Serratia proteamaculans 568	+	+	+	+	+	+	+	+	+	+	+	-					+	aroF-tyrA; tyrP; tyrR; aroP; aroL; COG2814
Erwinia carotovora SCRI1043	+	+	+	+	+	+	+	+	+	+	+	-					-	aroF-tyrA; tyrP; aroP; COG2814
Edwardsiella tarda EIB202	+	-	+	+	+	+	+	+	+	+	+	-					-	aroF-tyrA; tyrP; aroP; COG2814
Proteus mirabilis HI4320	+	+	+	+	+	+	+	+	+	+	-	-					-	aroF-tyrA; tyrP; tyrR; aroP; aroL
Photorhabdus luminescens TTO1	+	+	+	+	+	+	+	+	+	+	-	-					-	aroF-tyrA; tyrP; tyrR; aroL

Vibrionales	TyrR	AroA	AroH	AroG	AroF	TyrA	TyrB	TyrP	COG0733			Hpd	HmgA	HmgB	HmgC	PhhAB		TyrR regulon
Vibrio cholerae N16961	+	+	+	-	+	+	+	+	+			+	+	+	+	+		aroF-tyrA; tyrP; COG733; phhAB; hpd-hmgACB
Vibrio vulnificus CMCP6	+	+	+	+	+	+	+	+	+			+	+	+	+	+		aroF-tyrA; tyrP; COG733; phhAB; hpd-hmgACB; aroG
Vibrio harveyi ATCC BAA-1116	+	+	+	+	+	+	+	+	+			+	+	+	+	+		aroF-tyrA; tyrP; COG733; phhAB; hpd-hmgACB
Vibrio parahaemolyticus RIMD 2210633	+	+	+	+	+	+	+	+	+			+	+	+	+	+		aroF-tyrA; tyrP; COG733; phhAB; hpd-hmgACB; aroG
Vibrio shilonii AK1	+	+	+	+	+	+	+	+	++			+	+	+	+	+		aroF-tyrA; tyrP; COG733-1, COG733-2; phhAB;hpd-hmgACB
Vibrio splendidus LGP32	+	+	+	+	+	+	+	+	+			+	+	+	+	+		aroF-tyrA; tyrP; COG733; phhAB; hpd-hmgACB; tyrR; aroG
Vibrio fischeri ES114	+	+	+	+	+	+	+	+	+			-	-	-	-	-		aroF-tyrA; tyrP; COG733; tyrR
Vibrio salmonicida LFI1238	+	+	+	+	+	+	+	+	+			-	-	-	-	-		aroF-tyrA; tyrP; COG733; tyrR
Vibrio angustum S14	+	+	+	+	+	+	+	+	+			+	+	+	+	+		aroF-tyrA; tyrP; COG733; phhAB; tyrR; hmgACB
Photobacterium profundum SS9	+	+	+	+	+	+	+	+	+			+	+	+	+	+		aroF-tyrA; tyrP; COG733; phhAB; hmgACB

Pasteurellales	TyrR	AroL	AroH	AroG	AroF	TyrA	TyrB	TyrP	Mtr			Tpl	Hpd	HmgA	HmgB	HmgC	PhhAB	TyrR regulon
Haemophilus influenzae Rd KW20	+		-	+	-	+	+	+	+			-						tyrA; tyrR
Aggregatibacter aphrophilus NJ8700	+		-	-	-	+	+	+	+			+						tyrR; tpl
Pasteurella multocida Pm70	+		-	+	+	+	+	+	+			+						aroF-tyrA; tyrP2; tyrR; tpl
Mannheimia succiniciproducens MBEL55E	+		-	+	+	+	+	+	+			-						aroF-tyrA; tyrP2; tyrR
Actinobacillus succinogenes 130Z	+		-	+	+	+	+	+	+			-						aroF-tyrA; tyrR
Haemophilus somnus 2336	+		-	+	-	+	+	+	+			-						tyrA; tyrP1
Actinobacillus pleuropneumoniae AP76	+		-	+	+	+	+	++	+			-						aroF-tyrA; tyrP1-tyrP2; tyrR
Haemophilus ducreyi 35000HP	+		-	-	+	-	-	+	-			-						aroF; tyrR
Haemophilus parasuis SH0165	+		-	-	+	+	+	++	-			-						aroF-tyrA; tyrP1; tyrP2; tyrR

Shewanellaceae	TyrR	AroA	AroH	AroG	AroF	TyrA	TyrB				IpdC	Hpd	HmgA	HmgB	HmgC	PhhAB	Tdo-kyn	TyrR; HmgQ regulon
Shewanella oneidensis MR-1	+	+	+	+	+	+	+				-	+	+	+	+	+	+	tyrR; phhAB; hmgCB; aroA; tyrB; tdo-kyn; hmgA-hpd; hmgQ
Shewanella putrefaciens CN-32	+	+	+	+	+	+	+				-	+	+	+	+	+	+	tyrR; phhAB; hmgCB; aroA; tyrB; tdo-kyn; hmgA-hpd; hmgQ
Shewanella sp W3-18-1	+	+	+	+	+	+	+				-	+	+	+	+	+	+	tyrR; phhAB; hmgCB; aroA; tyrB; tdo-kyn; hmgA-hpd; hmgQ
Shewanella sp ANA-3	+	+	+	+	+	+	+				-	+	+	+	+	+	+	tyrR; phhAB; hmgCB; aroA; tyrB; tdo-kyn; hmgA-hpd; hmgQ
Shewanella sp MR-4	+	+	+	+	+	+	+				-	+	+	+	+	+	+	tyrR; phhAB; hmgCB; aroA; tyrB; tdo-kyn; hmgA-hpd; hmgQ
Shewanella sp MR-7	+	+	+	+	+	+	+				-	+	+	+	+	+	+	tyrR; phhAB; hmgCB; aroA; tyrB; tdo-kyn; hmgA-hpd; hmgQ
Shewanella baltica OS155	+	+	+	+	+	+	+				-	+	+	+	+	+	+	tyrR; phhAB; hmgCB; aroA; tyrB; tdo-kyn; hmgA-hpd; hmgQ
Shewanella denitrificans OS217	+	+	+	+	+	+	+				+	+	+	+	+	+	+	tyrR; phhAB; hmgCB; aroA; tyrB; tdo-kyn; ipdC; hmgA-hpd; hmgQ
Shewanella frigidimarina NCIMB 400	+	+	+	+	+	+	+				-	+	+	+	+	+	-	tyrR; phhAB; hmgCB; aroA; tyrB; hmgA-hpd; hmgQ
Shewanella amazonensis SB2B	+	+	+	+	+	+	+				+	+	+	+	+	+	+	tyrR; phhAB; hmgCB; aroA; tyrB; ipdC; hmgA-hpd; hmgQ
Shewanella loihica PV-4	+	+	+	+	+	+	+				+	+	+	+	+	+	+	tyrR; phhAB; hmgCB; aroA; tyrB; ipdC; hmgA-hpd; hmgQ
Shewanella pealeana ATCC 700345	+	+	+	+	+	+	+				-	+	+	+	+	+	+	tyrR; phhAB; hmgCB; aroA; tyrB; hmgA-hpd; hmgQ
Shewanella halifaxensis HAW-EB4	+	+	+	+	+	+	+				-	+	+	+	+	+	-	tyrR; phhAB; hmgCB; tyrB; hmgA-hpd; hmgQ
Shewanella piezotolerans WP3	+	+	+	+	+	+	+				-	+	+	+	+	+	+	tyrR; phhAB; hmgCB; tyrB; hmgA-hpd; hmgQ
Shewanella sediminis HAW-EB3	+	+	+	+	+	+	+				+	+	-	+	+	+	-	tyrR; phhAB; hmgCB; aroA; tyrB; ipdC; hpd; hmgQ
Shewanella woodyi ATCC 51908	+	+	+	+	+	+	+				-	+	-	+	+	+	-	tyrR; phhAB; hmgCB; aroA; tyrB; hpd; hmgQ

Alteromonadales	TyrR	AroL	AroH	AroG	AroF	TyrA	TyrB	TyrP				Hpd	HmgA	HmgB	HmgC	PhhAB		TyrR1 regulon; HmgS regulon
Pseudoalteromonas atlantica T6c	+				+	+		-				+		+		+		tyrR; phhA; aroF-tyrA; hpd
Alteromonas macleodii 'Deep ecotype'	+				+	+		-				-		+	+	+		tyrR; phhA; aroF-tyrA
Glaciecola sp. HTCC2999	+				+	+		-				-						tyrR; aroF-tyrA
Colwellia psychrerythraea 34H	+				+	+		-				-		+	+	+		tyrR; phhA; aroF-tyrA
Alteromonadales bacterium TW-7	+				-	+		+				-	+	+		+		tyrR; phhAB; tyrA; tyrP; hmgAB
Pseudoalteromonas haloplanktis TAC125	+				-	+		+				-	+	+		+		tyrR; phhAB; tyrA; tyrP; hmgAB
Pseudoalteromonas tunicata D2	+				-	+		+				-	+	+		+		tyrR; phhAB; tyrA; tyrP; hmgAB
Idiomarina baltica OS145	+				-	+						+	+	+	+	+		tyrR2; mdeA-phhAB; hpd-hmgACB
Idiomarina loihiensis L2TR	+				-	-						+	+	+	+	+		mdeA-phhAB-tyrR2; hpd-hmgACB

Psychromonadaceae/Aeromonadales	TyrR	AroL	AroH	AroG	AroF	TyrA	TyrB	TyrP				Hpd	HmgA	HmgB	HmgC	PhhAB		TyrR regulon

	TyrR	AroL	AroH	AroG	AroF	TyrA	TyrB		COG2814	COG0733	Hpd	HmgA	HmgB	HmgC	PhhAB	
Psychromonas spp. (2 genomes)	-					+	+									
Moritella sp. PE36	+				+	+	+	+			+	+	+		+	tyrR; tyrP
Aeromonas hydrophila ATCC 7966	+			+	+	+	+				+	+	+	+	+	aroF-tyrA; phhAB; aroG; hpd-hmgACB
Aeromonas salmonicida A449	+			+	+	+	+				+	+	+	+	+	aroF-tyrA; phhAB; aroG; hpd-hmgACB
Tolumonas auensis DSM 9187	+			+	+	+	+									aroF-tyrA
**Oceanospirillales/Alteromonadales**	**TyrR**	**AroL**	**AroH**	**AroG**	**AroF**	**TyrA**	**TyrB**				**Hpd**	**HmgA**	**HmgB**	**HmgC**	**PhhAB**	No TyrR regulon
Hahella chejuensis KCTC 2396	-															
Marinobacter aqueolei	-															
Marinobacter sp. ELB17	-															
Oceanobacter sp. RED65	-															
Oceanospirillum sp. MED92	-															
Marinomonas sp. MWYL1	-															
Saccharophagus degradans 2-40	-															
Teredinibacter turnerae T7901	-															
Cellvibrio japonicus Ueda107	-															
Chromohalobacter salexigens DSM 3043	-															
Reinekea sp. MED297	-															
Alcanivorax borkumensis SK2	-															
**Pseudomonadaceae**	**PhhR**	**AroL**	**AroH**	**AroG**	**AroF**	**TyrA**	**TyrB**		**COG2814**	**COG0733**	**Hpd**	**HmgA**	**HmgB**	**HmgC**	**PhhAB**	**PhhR (TyrR) regulon; HmgR regulon; HmgQ regulon**
Pseudomonas aeruginosa PAO1	+								+		+	+	+	+	+	phhR; phhABC; hpd; hmgACB-COG2814; hmgR
Pseudomonas entomophila L48	+								+			+	+	+	+	phhR; phhABC; hmgACB-COG2814; hmgR
Pseudomonas putida KT2440	+											+	+	+	+	phhR; phhAB; hmgACB; hmgR
Pseudomonas syringae DC3000	+										+	+	+	+	+	phhR; phhAB; hmgAC; hmgB-hpd; hmgR
Pseudomonas fluorescens Pf-5	+								+			+	+	+	+	phhR; phhABC; hmgACB-COG2814; hmgR
Pseudomonas mendocina ymp	+									+	+	+	+	+	+	phhR; phhABC; hpd-hmgACB-COG733
Pseudomonas stutzeri A1501	+									+	+	+	+	+	+	phhR; phhABC; hpd-hmgACB-COG733
Azotobacter vinelandii AvOP	-										+	+*'	+	+	+	hmgQ; hpd-gloA-hmgCB
**Moraxellaceae**	**TyrR**	**AroL**	**AroH**	**AroG**	**AroF**	**TyrA**	**TyrB**				**Hpd**	**HmgA**	**HmgB**	**HmgC**	**PhhAB**	No TyrR regulon
Acinetobacter sp. ADP1	-															
Acinetobacter baumannii AB0057	-															
Psychrobacter arcticum 273-4	-															
Psychrobacter sp. PRwf-1	-															
**Xanthomonadales**	**TyrR**	**AroL**	**AroH**	**AroG**	**AroF**	**TyrA**	**TyrB**				**Hpd**	**HmgA**	**HmgB**	**HmgC**	**PhhAB**	No TyrR regulon
Xylella fastidiosa 9a5c	-															
Xanthomonas axonopodis 306	-															
Xanthomonas campestris ATCC 33913	-															
Stenotrophomonas maltophilia K279a	-															

The presence or absence of gene orthologs is shown by '+/-' signs. Colored '+' signs (red, purple, green, blue) inidcate that the gene belong to TyrR(PhhR), HmgR, HmgQ, HmgS regulons, respectively.

Last column shows the list of all corresponding operons from recontructed regulons. Additional member of TyrR regulons that are not involved in aromatic amino acid metabolism are not included.

Color code for genes in the table:

- TyrR (PhhR) regulator gene
- Chorismate biosynthesis genes
- Tyr biosynthesis pathway genes
- Tyr transporter genes
- Tyr degradation genes
